# Supplementary material for: Prevalence of non-tuberculous mycobacteria among people with acid-fast positive presumptive tuberculosis in Mali
Source: PLoS One. 2024 Dec 18;19(12):e0301731. doi: 10.1371/journal.pone.0301731 (PMC11654988; doi:10.1371/journal.pone.0301731)
Supplement: Attachment — Submitted filename: PONE-D-24-10019_R1.pdf [file pone.0301731.s003.pdf]

PLOS ONE

Prevalence of non-tuberculous mycobacteria among people with acid-fast positive presumptive tuberculosis in Mali

--Manuscript Draft--

**Manuscript Number:**	PONE-D-24-10019R1
**Article Type:**	Research Article
**Full Title:**	Prevalence of non-tuberculous mycobacteria among people with acid-fast positive presumptive tuberculosis in Mali
**Short Title:**	Prevalence of non-tuberculous mycobacteria among people with presumptive tuberculosis in Mali
**Corresponding Author:**	Aissata Boubakar CISSE, PharmD,Mcs, doctoral student<br>National Institute of Public Health: Institut National de Sante Publique<br>Bamako, MALI
**Keywords:**	Acid-fast bacilli; Mali; non-tuberculous mycobacteria; Drug resistance survey.
**Abstract:**	Background: Non-tuberculous mycobacteria (NTM) are environmental agents that can cause opportunistic pulmonary disease in humans and animals which is often misdiagnosed as tuberculosis (TB). In this study, we describe the cases of NTM identified during the first national anti-TB drug resistance survey conducted in Mali and explore associated risk factors.<br>Methods: Sputum was collected from people presenting for pulmonary TB diagnosis, from April to December 2019, regardless of age. Microscopy-positive patients were enrolled and were tested by the Xpert MTB/RIF assay. Patients who tested negative for the Mycobacterium tuberculosis complex (MTBc) were tested for the presence of mycobacteria by amplification of the IS6110 and 16SrRNA (16S) genes through double quantitative real-time PCR, followed by nested PCR and Sanger sequencing of the IS6110-negative samples for NTM species identification.<br>Results: A total of 1,418 sputum smear-positive patients were enrolled, including 1,199 new cases, 211 previously treated cases and 8 whose previous treatment history was unknown. Based on the results of Xpert MTB/RIF assay and in-house PCR methods, 1,331 (93.9%) patients were positive for MTBc, 48 (3.4%) for NTM and no species identification was possible for 39 (2.7%). Advanced age of 65 and over with an OR 8.8 (95% CI 2.3-33.2 and p=0.001) and previous TB treatment with an OR 3.4 (95% CI 1.2-9.6 and p=0.016) were the risk factors statistically associated with NTM detection. M. avium complex (MAC) was the predominant NTM species, detected in 20 cases.<br>Conclusion: Detection of NTM in people presumed to have TB is an ongoing challenge, confounding correct TB diagnosis. Concomitant use of microscopy and GeneXpert testing among at-risk individuals could improve patient management.
**Order of Authors:**	Aissata Boubakar CISSE, PharmD,Mcs, doctoral student
	Anna S. Dean, PhD
	Armand Van Deun, PhD
	Jelle Keysers
	Willem-Bram de Rijk, Bachelor
	Mourad Gumusboga, Bachelor
	Hawa SAMAKE, PhD
	Seydou ARAMA, PharmD
	Bassirou Diarra, MD, Msc, PhD
	Fatoumata N'Dji Coulibaly, Bachelor
	Ibrahim Djilla, Bachelor
	Hawa Simpara, Msc
	Mamadou Berthé, MD, Msc, PhD

Powered by Editorial Manager® and ProduXion Manager® from Aries Systems Corporation

	Khadidia Ouattara, MD
	Yacouba Toloba, MD
	Ibrehima Guindo, PharmD, Msc, PhD
	Bouke Catherine de Jong, MD PhD
	Leen Rigouts, PhD
**Response to Reviewers:**	The drug resistance survey protocol in which a secondary analysis of samples is described in the informed consent was accepted by the ethics committee of the National Institute of Public Health Research by decision No. 08/2018/CE-INRSP on March 21, 2018. The objectives and expected results of the study were clearly explained in the language understood by the patient. Patients were asked for permission to use their samples for additional analyses, including outside Mali. Oral and written consent were obtained from patients over 18 years of age. For patients under the age of 18, after obtaining the prior oral and written consent of the parent or companion, the child's assent to participate in the study was obtained in the presence of a witness other than the parent or companion.
**Additional Information:**	
**Question**	**Response**
**Financial Disclosure**<br><br>Enter a financial disclosure statement that describes the sources of funding for the work included in this submission. Review the submission guidelines for detailed requirements. View published research articles from *PLOS ONE* for specific examples.<br><br>This statement is required for submission and **will appear in the published article** if the submission is accepted. Please make sure it is accurate.<br><br>**Funded studies**<br>Enter a statement with the following details:<br>• Initials of the authors who received each award<br>• Grant numbers awarded to each author<br>• The full name of each funder<br>• URL of each funder website<br>• Did the sponsors or funders play any role in the study design, data collection and analysis, decision to publish, or preparation of the manuscript?<br><br>Did you receive funding for this work?	Yes
Please add funding details.<br>as follow-up to "**Financial Disclosure**	1st tuberculosis Drug resistance survey financed by the Global Fund

Enter a financial disclosure statement that describes the sources of funding for the work included in this submission. Review the submission guidelines for detailed requirements. View published research articles from *PLOS ONE* for specific examples.<br><br>This statement is required for submission and **will appear in the published article** if the submission is accepted. Please make sure it is accurate.<br><br>**Funded studies**<br>Enter a statement with the following details:<br>• Initials of the authors who received each award<br>• Grant numbers awarded to each author<br>• The full name of each funder<br>• URL of each funder website<br>• Did the sponsors or funders play any role in the study design, data collection and analysis, decision to publish, or preparation of the manuscript?<br><br>Did you receive funding for this work?"	
Please select the country of your main research funder (please select carefully as in some cases this is used in fee calculation).<br>as follow-up to "**Financial Disclosure**<br><br>Enter a financial disclosure statement that describes the sources of funding for the work included in this submission. Review the submission guidelines for detailed requirements. View published research articles from *PLOS ONE* for specific examples.<br><br>This statement is required for submission and **will appear in the published article** if the submission is accepted. Please make sure it is accurate.	MALI - ML

**Funded studies**<br>Enter a statement with the following details:<br>• Initials of the authors who received each award<br>• Grant numbers awarded to each author<br>• The full name of each funder<br>• URL of each funder website<br>• Did the sponsors or funders play any role in the study design, data collection and analysis, decision to publish, or preparation of the manuscript?<br><br>Did you receive funding for this work?"	
**Competing Interests**<br><br>Use the instructions below to enter a competing interest statement for this submission. On behalf of all authors, disclose any competing interests that could be perceived to bias this work—acknowledging all financial support and any other relevant financial or non-financial competing interests.<br><br>This statement is required for submission and **will appear in the published article** if the submission is accepted. Please make sure it is accurate and that any funding sources listed in your Funding Information later in the submission form are also declared in your Financial Disclosure statement.<br><br>View published research articles from *PLOS ONE* for specific examples.	The authors have declared that no competing interests exist.

**NO authors have competing interests**<br><br>Enter: *The authors have declared that no competing interests exist.*<br><br>**Authors with competing interests**<br><br>Enter competing interest details beginning with this statement:<br><br>*I have read the journal's policy and the authors of this manuscript have the following competing interests: [insert competing interests here]*<br><br>* typeset	
**Ethics Statement**<br><br>Enter an ethics statement for this submission. This statement is required if the study involved:<br><br>• Human participants<br>• Human specimens or tissue<br>• Vertebrate animals or cephalopods<br>• Vertebrate embryos or tissues<br>• Field research<br><br>Write "N/A" if the submission does not require an ethics statement.<br><br>General guidance is provided below. Consult the submission guidelines for detailed instructions. **Make sure that all information entered here is included in the Methods section of the manuscript.**	The drug resistance survey protocol in which a secondary analysis of samples is described in the informed consent was accepted by the ethics committee of the National Institute of Public Health Research by decision No. 08/2018/CE-INRSP on March 21, 2018. The objectives and expected results of the study were clearly explained in the language understood by the patient. Patients were asked for permission to use their samples for additional analyses, including outside Mali. Oral and written consent were obtained from patients over 18 years of age. For patients under the age of 18, after obtaining the prior oral and written consent of the parent or companion, the child's assent to participate in the study was obtained in the presence of a witness other than the parent or companion.

**Format for specific study types**<br><br>**Human Subject Research (involving human participants and/or tissue)**<br>• Give the name of the institutional review board or ethics committee that approved the study<br>• Include the approval number and/or a statement indicating approval of this research<br>• Indicate the form of consent obtained (written/oral) or the reason that consent was not obtained (e.g. the data were analyzed anonymously)<br><br>**Animal Research (involving vertebrate animals, embryos or tissues)**<br>• Provide the name of the Institutional Animal Care and Use Committee (IACUC) or other relevant ethics board that reviewed the study protocol, and indicate whether they approved this research or granted a formal waiver of ethical approval<br>• Include an approval number if one was obtained<br>• If the study involved *non-human primates*, add *additional details* about animal welfare and steps taken to ameliorate suffering<br>• If anesthesia, euthanasia, or any kind of animal sacrifice is part of the study, include briefly which substances and/or methods were applied<br><br>**Field Research**<br><br>Include the following details if this study involves the collection of plant, animal, or other materials from a natural setting:<br>• Field permit number<br>• Name of the institution or relevant body that granted permission	
**Data Availability**<br><br>Authors are required to make all data underlying the findings described fully available, without restriction, and from the time of publication. PLOS allows rare exceptions to address legal and ethical concerns. See the PLOS Data Policy and FAQ for detailed information.	Yes - all data are fully available without restriction

A Data Availability Statement describing where the data can be found is required at submission. Your answers to this question constitute the Data Availability Statement and **will be published in the article**, if accepted.<br><br>**Important:** Stating 'data available on request from the author' is not sufficient. If your data are only available upon request, select 'No' for the first question and explain your exceptional situation in the text box.<br><br>Do the authors confirm that all data underlying the findings described in their manuscript are fully available without restriction?	
**Describe where the data may be found in full sentences. If you are copying our sample text, replace any instances of XXX with the appropriate details.**<br><br>• If the data are **held or will be held in a public repository**, include URLs, accession numbers or DOIs. If this information will only be available after acceptance, indicate this by ticking the box below. For example: *All XXX files are available from the XXX database (accession number(s) XXX, XXX.).*<br>• If the data are all contained **within the manuscript and/or Supporting Information files**, enter the following: *All relevant data are within the manuscript and its Supporting Information files.*<br>• If neither of these applies but you are able to provide **details of access elsewhere**, with or without limitations, please do so. For example:<br><br>*Data cannot be shared publicly because of [XXX]. Data are available from the XXX Institutional Data Access / Ethics Committee (contact via XXX) for researchers who meet the criteria for access to confidential data.*<br><br>*The data underlying the results presented in the study are available from (include the name of the third party*	All relevant data are within the manuscript and its Supporting Information files.

and contact information or URL).<br>• This text is appropriate if the data are owned by a third party and authors do not have permission to share the data.<br><br>* typeset	
Additional data availability information:	Tick here if your circumstances are not covered by the questions above and you need the journal's help to make your data available.

Powered by Editorial Manager® and ProduXion Manager® from Aries Systems Corporation

Manuscript Click here to access/download;Manuscript;Manuscrit.rtf

Prevalence of non-tuberculous mycobacteria among people with acid-fast positive presumptive tuberculosis in Mali

Prevalence of non-tuberculous mycobacteria among people with presumptive tuberculosis in Mali

Aissata Boubakar CISSE[1]*, Anna S. DEAN[2], Armand Van DEUN[3†], Jelle KEYSERS[3], Willem-Bram De RIJK[3], Mourad GUMUSBOGA[3], Hawa SAMAKE[1], Seydou ARAMA[1], Bassirou DIARRA[5], Ibrahim DJILLA[1], Fatoumata N. COULIBALY[1], Hawa SIMPARA[1], Mamadou BERTHE[1], Khadidia OUATTARA[6], Yacouba TOLOBA[6], Ibrehima GUINDO[1], Bouke de JONG[3], Leen RIGOUTS[3,4].

1- National Institute of Public Health, Bamako, Mali
2- Word Health Organization, Geneva
3- Mycobacteriology Unit, Institute of Tropical Medicine, Antwerp, Belgium
4- Belgian Coordinated Collections of Microorganisms, Antwerp, Belgium
5- University Clinical Research Center, Bamako, Mali
6- University Teaching hospital of Point G, Bamako, Mali

† Deceased in September 2023

* Corresponding author

E-mail: aissatacisse@yahoo.fr (ABC)

Abstract

Background: Non-tuberculous mycobacteria (NTM) are environmental agents that can cause opportunistic pulmonary disease in humans and animals which is often misdiagnosed as tuberculosis (TB). In this study, we describe the cases of NTM identified during the first national anti-TB drug resistance survey conducted in Mali and explore associated risk factors.

Methods: Sputum was collected from people presenting for pulmonary TB diagnosis, from April to December 2019, regardless of age. Microscopy-positive patients were enrolled and were tested by the Xpert MTB/RIF assay. Patients who tested negative for the *Mycobacterium tuberculosis* complex (MTBc) were tested for the presence of mycobacteria by amplification of the IS*6110* and 16SrRNA (16S) genes through double quantitative real-time PCR, followed by nested PCR and Sanger sequencing of the IS*6110*-negative samples for NTM species identification.

Results: A total of 1,418 sputum smear-positive patients were enrolled, including 1,199 new cases, 211 previously treated cases and 8 whose previous treatment history was unknown. Based on the results of Xpert MTB/RIF assay and in-house PCR methods, 1,331 (93.9%) patients were positive for MTBc, 48 (3.4%) for NTM and no species identification was possible for 39 (2.7%). Advanced age of 65 and over with an OR 8.8 (95% CI 2.3-33.2 and p=0.001) and previous TB treatment with an OR 3.4 (95% CI 1.2-9.6 and p=0.016) were the risk factors statistically associated with NTM detection. *M. avium complex* (MAC) was the predominant NTM species, detected in 20 cases.

Conclusion: Detection of NTM in people presumed to have TB is an ongoing challenge, confounding correct TB diagnosis. Concomitant use of microscopy and GeneXpert testing among at-risk individuals could improve patient management.

1. Introduction

The *Mycobacterium* genus groups together species of the *Mycobacterium tuberculosis* complex (MTBc) responsible for tuberculosis (TB) in humans and animals, *M. leprae*, the causative agent of leprosy, and the species of mycobacteria commonly known as atypical or non-tuberculous mycobacteria (NTM), including *M. ulcerans* responsible for Buruli ulcer. Unlike members of the MTBc group, NTM are not obligatory parasites of humans, yet normal residents of soil and water, and can be found in natural and treated water sources [1]. More than 200 species of NTM have been officially recognised [2], of which around 25 are known to be strongly associated with disease in humans. Some species have been associated with pulmonary diseases causing symptoms similar to TB [1]. Because of their habitat, humans are exposed to these bacteria on a daily basis, and NTM disease must therefore be distinguished from a simple colonisation or contamination of clinical samples, for example from tap water [1,3]. Unlike for TB, the global epidemiology of disease caused by NTM is not well known. NTM isolation from clinical specimens is mostly reported in industrialised countries, with variable prevalence and incidence. Studies based on pulmonary specimen isolates, reported a prevalence of 1.4 to 6.6 per 100,000 persons in the USA from 2004 to 2006 [4], 9.8 in 2010 in Ontario, Canada [5] and 5.8 per 100,000 in Germany [6] in 2020. Incidence of 6.1 per 100,000 in 2012 in England [7] and 5.3 per 100, 000 in Germany in 2020 [6] have also reported. In TB endemic countries, NTM are being reported less frequently and mainly in populations at-risk, particularly people with predisposing

conditions or compromised immunity [8]. However, experiences in industrialised countries have shown that a declining TB burden has also resulted in an increase in the number of NTM cases detected. With the strengthening of TB programmes in other setting, we may also anticipate a similar scenario, posing a growing diagnosis and clinical challenge in low-and middle-income countries [9]. The diagnosis of NTM lung disease is based on clinical, radiological and microbiological criteria [1]. Laboratory based detection of NTM, with differentiation from MTBc and identification to the species level, is largely inaccessible in most resource-limited countries. Microscopy, the most accessible technique, identifies both MTBc and NTM as acid-fast bacilli (AFB), but fails to distinguish them. Since 2010, the World Health Organization (WHO) recommends rapid molecular tests such as GeneXpert MTB/RIF (Xpert) as the initial test for TB diagnosis, exhibiting a higher sensitivity and specificity [10]. This assay only identifies the presence of MTBc species in the sample. A negative result for MTBc by Xpert obtained on AFB-positive sputum could therefore be indicative of NTM [11]. In Mali, cases of NTM infection have been described, particularly in patients under investigation for failure of anti-TB treatment or who have relapsed after TB cure [12]. Following the introduction of Xpert in the country, plausible cases of NTM infection with AFB-positive smear and a negative Xpert have been reported more frequently [13].

In this study, we describe the proportion of MTBc and NTM detected in patients presenting for initial or previously treated TB diagnosis in Mali, in the context of a nationwide drug resistance survey [14]. We also explored risk factors associated with NTM detection.

2. Methods

2.1. Design of the study

From April to December 2019 consecutive eligible patients were recruited from 78 of 86 TB diagnostic laboratories in the country, representing 91% of the country's TB laboratory network. Eight laboratories notified less than 10 new bacteriologically confirmed pulmonary TB cases in 2017 and were not included for logistic reasons. Eligible participants were defined as people undergoing evaluation for pulmonary TB, both with and without a history of prior treatment, regardless of age. The sample size of 994 new cases was calculated to meet the primary objective of the survey for measuring the percentage of new pulmonary TB cases resistant to rifampicin, for which a recruitment period of 4 months was estimated to be required [15].

2.2. Sample collection and patient enrolment

Two sputum samples, each of approximately 5 mL, were collected from each patient in the participating laboratories. Smears were made directly from freshly collected sputum, stained according to the acid-fast staining technique used at the center (auramine for most sites and Ziehl-Neelsen for several small centers), and in line with international guidelines for diagnosing TB by microscopy [16]. In case one of these two sputum samples was AFB positive, the patient was included in the drug resistance survey after giving informed consent, and an additional sample was collected.

The nurse completed the study questionnaire with the recruited patient. The questionnaire covered socio-demographic data, and information on previous TB treatment and pulmonary disease history (duration and chronicity of respiratory signs). Relevant clinical and treatment-

related information was also explained to the patient. All enrolled participants received anti-TB treatment. New patients were started on first-line TB treatment on the basis of an AFB microscopy. For patients with previously treated TB, in accordance with national guidelines, treatment was initiated based on the result of Xpert MTB/RIF testing.

2.3. Initial GeneXpert testing and specimen storage

The first two sputum samples collected were preserved in 5 mL of OMNIgene® SPUTUM (OM-S) decontamination reagent (DNA Genotek Inc, Canada) according to the manufacturer's recommendations [17], while the third sample was preserved in 10 mL of 96% ethanol. The conditioned samples were transported by public transport at ambient temperature in triple packaging to the National TB Reference Laboratory (NTRL) in Bamako.

The Xpert MTB/RIF assay (Cepheid, CA, USA) was performed at the NTRL using 2 mL of one OM-S-preserved sample, in line with the manufacturer's recommendations (Cepheid and Genotek).

Leftovers of the OM-S-preserved samples were then stored in cryotubes in the form of sediment obtained after centrifugation at 4000 g for 15 minutes, and mixed with 96% alcohol at an equal volume (0.5 mL sediment and 0.5 mL ethanol).

2.4. Molecular detection and identification of NTM species

For molecular detection and identification of NTM species, according to the study protocol, samples from the following patients were transported at ambient temperature to the WHO Supranational TB Reference Laboratory at the Institute of Tropical Medicine (ITM) in Antwerp, Belgium:

- cases with rifampicin resistance by Xpert MTB/RIF, regardless of anti-TB treatment history;
- all previously treated cases, regardless of Xpert MTB/RIF test result;
- and cases for whom MTBc was not detected by Xpert MTB/RIF.

In this article, we described only the employed molecular methods and obtained results for species identification of probable NTM.

Upon arrival at ITM, ethanol-preserved samples were centrifuged at 4000 g for 15 minutes, digested by overnight proteinase K incubation, followed by lysis using an internal lysis buffer, after which DNA was extracted using the Modified Maxwell-LEV® automated extraction method (Promega, USA) [18]. A duplex quantitative real-time polymerase chain reaction (qPCR) was performed, targeting the IS*6110* gene for the detection of the MTBc and the 16S gene to detect all species of the *Mycobacterium* genus. The primers and probes used are presented in Table 1, while interpretation of results is depicted in Table 2.

Table 1. Primers and probes for qPCR to simultaneously detect *Mycobacterium tuberculosis* complex and all mycobacterial species.

Target	Name	Sequences
**IS*6110* gene**	IS*6110*_S<br>IS*6110*_AS	5'-CCTGAAAGACGTTATCCACCATAC-3'<br>5'-GGC-TAG-TGC-ATT-GTC-ATA-GGA-G-3'
	IS6110_P_DFO	DFO-TCT-CAG-TA(lnC)-AC(lnA)-TC(lnG)-ATC-CGG-T-BHQ2
**16S gene**	Myco_Genus_S_v3<br>Myco_Genus_AS_v2	5'-ATGCAAGTCGAACGGAAAGG-3'<br>5'-TCTGCCCGTATCGCC-C-3'
	Myco_Genus_P_v2	FAM-CAA-AC(lnC)-AC(lnC)-TA(lnC)-GA(lnG)-CTC-EDQ

Table 2. Interpretation of duplex IS*6110*-16S qPCR results

**16S qPCR Cycle threshold (Ct)**	**IS*6110* qPCR result**	
	**Positive**	**Negative**
**Positive with any Ct value**	-	No MTBc
**Positive and ≥IS*6110* Ct**	MTBc	
**Positive and <IS*6110* Ct**	MTBc + NTM	
**Negative**	MTBc (low bacillary load)	No mycobacteria species identification possible

MTBc = *Mycobacterium tuberculosis* complex; NTM = non-tuberculous mycobacteria: Ct = Cycle threshold

Subsequently, a nested conventional PCR targeting the 16S gene was performed on samples identified as NTM by the duplex qPCR. The primers and probes used were as follows:

- P1 (first PCR): 5'-TGCTTAACACATGCAAGTCG-3'
- P2 new (first PCR): 5'-TCTCTAGACGCGTCCTGTGC-3'
- P7 (nested PCR): 5'-CATGCAAGTCGAACGGAAAGG-3'
- P16 new (nested PCR): 5'-AAGCCGTGAGATTTCACGAACA3'

Amplicons that were clearly positive on the agarose gel underwent Sanger sequencing. A negative PCR result was interpreted as the absence of MTBc or NTM.

Sequencing was subcontracted to BaseClear (BE Leiden, The Netherlands). The results obtained after entering the sequence into the Basic Local Alignment Search Tool (BLAST) of the National Centre for Biotechnology Information (NCBI) were interpreted as follows:

- If a sequence match was found on the NCBI, the NTM species was identified.
    - In case of 100% similarity (no mismatches), the species name was assigned.

    - In case 1-3 bp mismatches with the closest species, the “species-like” name was assigned.

- If no match or >3 bp difference with the closest species was found on NCBI, the result was interpreted as *Mycobacterium* species.
- If there were overlapping peaks in the sequence, indicating the presence of several NTM, the result was interpreted as "Mixture of NTM ".

2.5. Data analysis

The data collected using the survey forms and sample submission forms were entered into EpiInfo version 7.2.5.0. The variables analysed were age, sex, region of residence, history of illness and history of anti-TB treatment. Percentages, medians and means were calculated by crosstab, and p-values and odds ratios with 95% confidence intervals were generated using regression analysis in IBM SPSS Statistics version 21X64 (IBM Corp., NY, USA).

2.6. Ethical considerations

The drug resistance survey protocol in which a secondary analysis of samples is described in the informed consent was accepted by the ethics committee of the National Institute of Public Health Research by decision No. 08/2018/CE-INRSP on March 21, 2018. The objectives and expected results of the study were clearly explained in the language understood by the patient. Patients were asked for permission to use their samples for additional analyses, including outside Mali. Oral and written consent were obtained from patients over 18 years of age. For patients under the age of 18, after obtaining the prior oral and written consent of the parent or companion, the child's assent to participate in the study was obtained in the presence of a witness other than the parent or companion.

3. Results

3.1. Mycobacteria detected

A total of 1,418 AFB-positive people with presumed pulmonary TB were enrolled in the drug resistance survey, including 1,199 new cases, 211 previously treated cases and 8 whose previous treatment history was unknown (Figure 1). Based on Xpert MTB/RIF results, 1,296 (91.4%) patients were positive for MTBc, while 1MTBc was not detected for 22 (8.6%). The duplex IS6110-16S qPCR performed for 119 of these 122 patients, identified another 35 MTBc, bringing the total number of patients with MTBc to 1,331 (93.9%). Of these 35 additional cases, MTBc alone was detected for six, while 29 carried probable mixtures of MTBc and NTM.

Fig 1. Sample and analysis flow with summary of results, MTBc=*Mycobacterium tuberculosis* complex; n= number; NTM = non-tuberculous mycobacteria.

For 81 (68,1%) of 119 patients tested, only the 16S target reacted positive (presumed NTM), while the PCR remained negative for both targets in 3 (2,5%) patients. The nested conventional 16S PCR performed for the 81 presumed NTM detected by IS*6110*-16S qPCR, was positive for 48 or 3.4% (48/1418) of all enrolled patients, of which 33 could be identified to the species level by sequencing. The remaining 33 of 81 presumed NTM, had a negative nested PCR result, thus no sequencing could be performed (Fig 1).

Thus, eleven species or groups of species could be identified, with predominance of the *M. avium* complex (MAC) representing 20 cases or 42% of all species detected, *M. intracellulare* complex in 13 patients (27%), *M. avium* in five (10%) and *M. colombiense* in two (4 %) (Fig 2).

Fig 2. Sequencing results for the identification of NTM species

3.2. Detection of NTM in relation to smear-microscopy results

The quantification of AFB was notified for 1414 of 1418 patients by the participating laboratories. A significantly higher proportion of NTM was detected in patients with lower AFB-grades: 7% for 1+ and scanty (Table 3), compared to 2% among 3+ patients (respectively OR 4.065, 95% CI: 1.876-8.808, p=0.000 and OR 4.3, 95% CI: 2.012-9.189, p=0.000). The difference was not significant compared to grade 2+ samples (p=0.462).

Table 3. Proportion of NTM and MTB detection in relation to quantitative sputum-smear microscopy results

Final sputum smear result	MTBc n (%)	NTM n (%)	No species identification possible n (%)	Total n (%)
**Scanty**	167 (83)	13 (7)	21 (10)	201
**1+**	170 (89)	14 (7)	8 (4)	192
**2+**	259 (94)	7 (3)	8 (3)	274
**3+**	731 (98)	14 (2)	2 (0)	747
**Not quantified**	0	0	4 (100)	4
**Total**	**1327 (93.6)**	**48 (3.4)**	**43 (3)**	**1418 (100)**

MTBc = *M. tuberculosis* complex; NTM = non-tuberculous mycobacteria; n =number

3.3. Socio-demographic, clinical characteristics and risk factors for NTM infection

Male patients were more common among study participants and among those with identified NTM, with a sex ratio of respectively 2.1 and 1.5 when compared to women, yet NTM presence was not associated with gender (Table 4). Contrary, older age was significantly associated with NTM detection. The median age was 37 years for all patients and 59.5 years for those with NTM detected. Compared to the under-34 age group, the 35-64 age group had a greater odds (OR 3.4, 95% CI: 1.1-10.3, p=0.03) of NTM detection, which further increased for the over-65 age group (OR 8.8, 95% CI: 2.3-33.2 p=0.001 (Table 4). Also, previously treated patients were more likely than new cases to have NTM in their sputum (OR 3.4, 95% CI: 1.2-9.6, p=0.016) (Table 4). We found no significant associations between NTM detection and HIV status or chronic respiratory signs. Regarding the patient's residence, patients from the Mopti region were more likely to have NTM detected compared to Bamako residents (OR 4.1, 95% CI: 1.0-16.4, p=0.04). (Table 4).

Table 4. Multivariate logistic regression analyses of demographic and clinical variables as risk factors of NTM infection

Variables		Total patients n (%)	NTM detected n (%)	OR	p-value
**Gender n=1379**	Female	434 (31.5)	19 (39.6%)	0.8 [0.3-2.04]	0.7
	Male	945 (68.5)	29 (60.4%)		
**Age n=1379**	Median	37	**59,5**		
**Age group**	0-34 years (Reference)	603 (43.7)	4 (8.3)		
	35-64 years	659 (47.8)	29 (60.4)	**3.4 [1.1-10.3]**	**0.03**
	≥65 years	117 (8.5)	15 (31.3)	**8.8 [2.3-33.2]**	**0.001**
**History of treatment n=1371**	New	1165 (84.5)	30 (62.5)		
	Previously treatment	206 (14.9)	18 (37.5)	**3.4 [1.2-9.6]**	**0.016**
**HIV status n=1092**	Positive	77 (5.6)	4 (8.3)	2.8 [0.8-8.9]	0.07
	Negative	1015 (73.6)	29 (60.4)		
**Chronic clinical signs n=1174**	Yes	442 (37.6)	24 (60)	0.7 [0.3-1.8]	0.5
	No	752 (62.4)	16 (40)		
**Region of residence at the time of diagnosis n=1348**	Bamako (Reference)	451 (33.5)	5 (10.9)		
	Kayes	101 (7.5)	4 (8.7)	2.7 [0.5-12.7]	0.2
	Koulikoro	217 (16.1)	7 (15.2)	1.5 [0.3-7.4]	0.5
	Sikasso	155 (11.5)	12 (26.1)	2.9 [0.7-10.8]	0.10
	Ségou	147 (10.9)	7 (15.2)	3.2 [0.8-12.7]	0.09
	Mopti	191 (14.2)	7 (15.2)	**4.1 [1.02-16.4]**	**0.04**
	Northern regions	86 (6.4)	4 (8.7)	4.2 [0.7-25.2]	0.1

NTM = non-tuberculous mycobacteria; n = number; OR = odds ratio

4. Discussion

The proportion of sputum-AFB-positive patients and MTBc detected by Xpert MTB/RIF among people presumed to have TB in our study was slightly lower than the rate reported in other drug resistance surveys, ranging from 95.8%-97.7% [19–22]. The confirmed proportion with NTM in our study was higher than in TB drug-resistance surveys in Pakistan (0.7%, 13/1972) [19] and in India (1.2%, 35/2,938) [23], yet lower than the 6.4% (317/4917) observed in China [24]. This result may reflect regional differences in these mycobacteria occurrence and diseases they cause around the world [25,26].

NTM detection was more likely among paucibacillary patients as determined by smear microscopy, corroborating findings from Nigeria where the AFB-smear grading was relatively lower for patients with NTM detected than for those with MTBc [27], and higher AFB grading was predictive for MTBc detection [28]. With age, the risk of developing NTM infection increased, but we did not find a significant association between gender and NTM infection, contrary to other studies, where females have been more frequently associated with NTM infections, especially at older ages [11,29,30]. Previous anti-TB treatment was another risk factor for NTM infection in our study, confirming findings from NTM pulmonary disease in China and South Africa [31,32]. Nevertheless, we did not find an association between NTM detection and chronic respiratory signs in our study, in contrast with the described association between NTM infection and the presence of lung disease or chronic respiratory symptoms in studies from China and Denmark [33,34].

HIV infection has frequently been identified as a risk factor for NTM infections and disease, especially in situations of advanced immunosuppression [27,35,36]. In our study, the trend towards an association between HIV-positivity and NTM detection was not significant,

corroborating previous observation in Mali [37]. In the context of the drug resistance survey, no information on anti-retroviral treatment was collected, but such treatment is to be started upon HIV diagnosis as per national guidelines in Mali. While patients from the Mopti region had a higher odds for having NTM detected in their sputum than those from the Bamako region, further investigation of the environmental conditions in these regions would be needed, especially as NTM are environmental bacteria and have often been isolated at different frequencies in different geographical areas [25,30,38]. MAC species were the most commonly NTM detected in our survey. The predominance of MAC has been reported in pulmonary NTM infections in many countries around the world [25,26,39,40]. In Mali, between 2004 and 2013, *M. avium* was the most frequently identified species of the MAC complex [12,37].

Employment of an additional in-house qPCR for IS*6110* in our study has allowed to increase the detection of MTBc. The MTBc-specific repetitive element IS*6110* is a suitable target to employ in MTBc detection assays [41]. The applied qPCR IS*6110* has shown good sensitivity and specificity for the detection of MTBc compared to culture [42] and higher performance when compared to the Xpert MTB/RIF assay [43]. The observed high Ct values in our study indicates, however, a low quantity of MTB DNA in samples from these patients, that may have been positive upon repeated Xpert testing [44] or by using the Xpert Ultra test, which is more sensitive by targeting both IS*6110* and another repetitive element IS*1081* for MTBc detection, in addition to the *rpoB* gene for assessing rifampicin susceptibility [45]. The higher positivity rate by qPCR compared to the conventional nested PCR we observed in our study, could be partially attributable to the non-specificity of the 16S primers employed for the in-house qPCR which also detects species belonging to closely related genera like *Rhodococcus* and *Nocardia*, hence potentially overestimating the NTM rate. These closely related species indeed can be detected in

smear-positive samples not containing tubercle bacilli, such as documented in the USA with 78.4% (134/171) of AFB-positive specimens yielding a positive culture with NTM or *Nocardia* spp, while 14.6% (25/171) of AFB-positive specimens grew MTBc and another 7% (12/171) remained negative in culture [46]. In another study in Nigeria, out of 32 smear-positive samples identified as not containing TB bacilli, six were NTM (18.75%), 14 were corynebacteria (43.75%) and 12 rhodococci (37.5%) [28]. In our study, we did not further identify the non-MTBc/non-NTM samples. In addition, the lower sensitivity of the conventional nested PCR may have hampered confirmation by sequencing in case of negative results or low amplicon yield. Indeed, the in-house qPCR has already shown good sensitivity, while limited loss of DNA has been described during multiple handling steps for conventional nested PCR [47,48]. This lower sensitivity is reflected in the sample's AFB-grading, with less non-identified samples among the strong positive specimens.

This study was part of the national TB drug resistance survey, comprising both new and previously treated cases. The concern of false-positive MTBc detection in sputum from previously treated patients has mostly been raised for the highly sensitive, newer generation of rapid molecular assays, such as the "MTB detected Trace" result by Xpert Ultra [49,50]. Nevertheless, even smear microscopy can still show non-viable AFB, and introduction of alternative/additional diagnostic assays or flows would need to be validated against culture.

Our survey was not designed to include culture in the diagnostic algorithm, and hence we could not assess the viability of the detected MTBc and/or NTM bacilli, nor whether co-detection of MTBc and NTM reflected simultaneous infection, or rather an NTM infection following MTBc infection in previously treated patients, which was still detectable due to remnant DNA.

Also, we did not investigate whether the detected NTM were attributable to true infection, colonization or sample contamination. We could not assess the American Thoracic Society criterium to detect NTM in at least 3 different samples per patient given the design of the survey and the retrospective nature of molecular analysis to detect NTM. However, study participants were people with respiratory signs undergoing evaluation for pulmonary TB attending health centers or patients failing TB treatment. Finally, we do not describe the outcome of patients in whom NTM was detected, but all were started on 1st line TB therapy or multidrug-resistant treatment following the results of initial microscopy and Xpert testing.

5. Conclusion

Among sputum-AFB-positive patients presenting in Mali, 3.4% were due to NTM. Particular attention must be paid to populations at risk, such as elderly patients, and patients with a history of previous treatment. TB prevention and care programmes in resource-limited countries should ensure that their routine diagnostic algorithms can detect such cases, based on the concurrent use of rapid molecular TB diagnostics and microscopy in the absence of other molecular assays to detect NTM. However, culture is still necessary to isolate these mycobacteria and to monitor treatment in the event of confirmation of NTM disease. Clinical assessment and management of NTM infections, remain a challenge worldwide.

Acknowledgements

We would like to thank the TB management sites for recruiting patients, collecting and transporting samples, the country's regional health departments for regional coordination of the

drug resistance study and Olga Tosas AUGUET for her assistance in planning the survey, monitoring the field activities and preliminary data analysis”.

References

1. Griffith DE, Aksamit T, Brown-Elliott BA, Catanzaro A, Daley C, Gordin F, et al. An official ATS/IDSA statement: Diagnosis, treatment, and prevention of nontuberculous mycobacterial diseases. Am J Respir Crit Care Med. 2007;175: 367–416. doi:10.1164/rccm.200604-571ST

2. Pascapurnama DN, Zavitri NG, Koesoemadinata RC, Cahyadi AI, Chaidir L. Identification of Significant Pathogenic Nontuberculous Mycobacteria Species from Presumptive TB Patients Using Partial hsp65 Gene Sequencing. Infect Drug Resist. 2023;16. doi:10.2147/IDR.S419956

3. Haworth CS, Banks J, Capstick T, Fisher AJ, Gorsuch T, Laurenson IF, et al. British Thoracic Society guidelines for the management of non-tuberculous mycobacterial pulmonary disease (NTM-PD). Thorax. 2017;72: iii1-ii64. doi:10.1136/thoraxjnl-2017-210927

4. Prevots DR, Shaw PA, Strickland D, Jackson LA, Raebel MA, Blosky MA, et al. Nontuberculous mycobacterial lung disease prevalence at four integrated health care delivery systems. Am J Respir Crit Care Med. 2010;182: 970–976. doi:10.1164/rccm.201002-0310OC

5. Marras TK, Mendelson D, Marchand-Austin A, May K, Jamieson FB. Pulmonary nontuberculous mycobacterial disease, Ontario, Canada, 1998-2010. Emerg Infect Dis. 2013;19: 1889–1891. doi:10.3201/eid1911.130737

6. Corbett C, Finger P, Heiß-Neumann M, Bohnert J, Eder IB, Eisele M, et al. Development of prevalence and incidence of non-tuberculous mycobacteria in German laboratories from 2016 to 2020. Emerg Microbes Infect. 2023;12. doi:10.1080/22221751.2023.2276342

7. Shah NM, Davidson JA, Anderson LF, Lalor MK, Kim J, Thomas HL, et al. Pulmonary Mycobacterium avium-intracellulare is the main driver of the rise in non-tuberculous mycobacteria incidence in England, Wales and Northern Ireland, 2007-2012. BMC Infect Dis. 2016;16. doi:10.1186/s12879-016-1521-3

8. Varma-Basil M, Bose M. Mapping the Footprints of Nontuberculous Mycobacteria. Nontuberculous Mycobacteria (NTM): Microbiological, Clinical and Geographical Distribution. Elsevier; 2019. pp. 155–175. doi:10.1016/B978-0-12-814692-7.00008-5

9. Iseman MD, Marras TK. The importance of nontuberculous mycobacterial lung disease. Am J Respir Crit Care Med. 2008;178: 999–1000. doi:10.1164/rccm.200808-1258ED

10. WHO. Module 3: Diagnosis WHO operational handbook on tuberculosis Rapid diagnostics for tuberculosis detection. 2021.

11. Kong L, Xie B, Liu Q, Hua L, Bhusal A, Bao C, et al. Application of acid-fast staining combined with GeneXpert MTB/RIF in the diagnosis of non-tuberculous mycobacteria pulmonary disease. International Journal of Infectious Diseases. 2021;104: 711–717. doi:10.1016/j.ijid.2020.12.091

12. Maiga M, Siddiqui S, Diallo S, Diarra B, Traoré B, Shea YR, et al. Failure to recognize nontuberculous mycobacteria leads to misdiagnosis of chronic pulmonary tuberculosis. PLoS One. 2012;7: e36902. doi:10.1371/journal.pone.0036902

13. Toloba Y, Cissé AB, Soumaré D, Ouattara K, Kanouté T, Koné D, et al. Apport du GeneXpert dans le diagnostic de la tuberculose et de la résistance à la rifampicine au Mali après 22 mois d'acquisition. Rev Mal Respir. 2017;34: A216–A217. doi:10.1016/j.rmr.2016.10.515

14. World Health Organization. Global tuberculosis report 2020. World Health Organization; 2020.

15. World Health Organization. Guidelines for surveillance of drug resistance in tuberculosis 5 th Edition. 2015.

16. Lumb R, Van Deun A, Bastian I, Fitz-Gerald M, Pathology SA, Stop TB Partnership (World Health Organization). Laboratory diagnosis of tuberculosis by sputum microscopy : the handbook. 2013.

17. DNA Genotek Inc. How can I use OMNIgene®•SPUTUM? Use Case 4: Reflex testing: Microscopy centre with or without GeneXpert® centrifuge. 2016 [cited 4 Apr 2017]. Available: www.dnagenotek.com•

18. Corporation P. Maxwell(R) 16 Cell LEV DNA Purification Kit Technical Bulletin TB383. 2012 [cited 11 Mar 2024]. Available: www.promega.com

19. Tahseen S, Qadeer E, Khanzada FM, Rizvi AH, Dean A, Van Deun A, et al. Use of Xpert® MTB/RIF assay in the first national antituberculosis drug resistance survey in Pakistan. International Journal of Tuberculosis and Lung Disease. 2016;20: 448–455. doi:10.5588/ijtld.15.0645

20. Lopes C, Kundu D, Da Costa Barreto I, da Cruz B, Siva Kumar S, Ramalingam S, et al. Experience on the first national anti-TB drug resistance survey (DRS) in Timor-Leste. Glob Health Res Policy. 2022;7: 2–9. doi:10.1186/s41256-022-00249-z

21. Aia P, Kal M, Lavu E, John LN, Johnson K, Coulter C, et al. The burden of drug-resistant tuberculosis in Papua New Guinea: Results of a large population-based survey. PLoS One. 2016;11: 2–9. doi:10.1371/journal.pone.0149806

22. Iem V, Dean A, Zignol M, Vongvichit P, Inthavong D, Siphanthong S, et al. Low prevalence of MDR-TB in Lao PDR: results from the first national anti-tuberculosis drug resistance survey. Tropical Medicine and International Health. 2019;24: 421–431. doi:10.1111/tmi.13205

23. Sharma M, Malhotra B, Tiwari J, Bhargava S. Profile of Nontuberculous Mycobacteria in Patients Suspected of Tuberculosis and Drug-Resistant Tuberculosis. J Lab Physicians. 2020;12: 203–211. doi:10.1055/s-0040-1721160

24. Liu CF, Song YM, He WC, Liu DX, He P, Bao JJ, et al. Nontuberculous mycobacteria in China: incidence and antimicrobial resistance spectrum from a nationwide survey. Infect Dis Poverty. 2021;10. doi:10.1186/s40249-021-00844-1

25. Hoefsloot W, Van Ingen J, Andrejak C, Ängeby K, Bauriaud R, Bemer P, et al. The geographic diversity of nontuberculous mycobacteria isolated from pulmonary samples: An NTM-NET collaborative study. European Respiratory Journal. 2013;42: 1604–1613. doi:10.1183/09031936.00149212

26. Okoi C, Anderson STB, Antonio M, Mulwa SN, Gehre F, Adetifa IMO. Non-tuberculous Mycobacteria isolated from Pulmonary samples in sub-Saharan Africa - A Systematic Review and Meta Analyses. Sci Rep. 2017;7. doi:10.1038/s41598-017-12175-z

27. Aliyu G, El-Kamary SS, Abimiku A, Brown C, Tracy K, Hungerford L, et al. Prevalence of Non-Tuberculous Mycobacterial Infections among Tuberculosis Suspects in Nigeria. PLoS One. 2013;8: e63170. doi:10.1371/journal.pone.0063170

28. Dinic L, Idigbe OE, Meloni S, Rawizza H, Akande P, Eisen G, et al. Sputum Smear Concentration May Misidentify Acid-Fast Bacilli As Mycobacterium Tuberculosis in HIV-Infected Patients. J Acquir Immune Defic Syndr. 2013;63: 168–177. doi:DOI:10.1097/QAI.0b013e31828983b9

29. Kennedy TP, Weber DJ. Nontuberculous mycobacteria. An underappreciated cause of geriatric lung disease. Am J Respir Crit Care Med. 1994;149: 1654–1658. doi:10.1164/ajrccm.149.6.8004325

30. Limo JJ, Nyerere A, Bii C. Prevalence, Species Characterization and factors associated with Non tuberculous mycobacteria among tuberculosis retreatment patients submitting samples to the National Tuberculosis Reference Laboratory, Kenya. Jomo Kenyatta University of Agriculture and Technology. 2016. Available: http://ir.jkuat.ac.ke

31. Xu J, Li P, Zheng S, Shu W, Pang Y. Prevalence and risk factors of pulmonary nontuberculous mycobacterial infections in the Zhejiang Province of China. Epidemiol Infect. 2019;147: e269. doi:10.1017/S0950268819001626

32. Corbett EL, Churchyard GJ, Clayton T, Herselman P, Williams B, Hayes R, et al. Risk Factors for Pulmonary Mycobacterial Disease in South African Gold Miners A Case-Control Study. Am J Respir Crit Care Med. 1999;159: 94–99. doi:https://doi.org/10.1164/ajrccm.159.1.9803048

33. Andréjak C, Nielsen R, Thomsen V, Duhaut P, Sørensen HT, Thomsen RW. Chronic respiratory disease, inhaled corticosteroids and risk of non-tuberculous mycobacteriosis. Thorax. 2013;68: 256–262. doi:10.1136/thoraxjnl-2012-201772

34. Feng JY, Chen WC, Chen YY, Su WJ. Clinical relevance and diagnosis of nontuberculous mycobacterial pulmonary disease in populations at risk. Journal of the Formosan Medical Association. 2020;119: S23–S31. doi:10.1016/j.jfma.2020.05.012

35. Hoza AS, Mfinanga SGM, Rodloff AC, Moser I, König B. Increased isolation of nontuberculous mycobacteria among TB suspects in Northeastern, Tanzania: Public health and diagnostic implications for control programmes. BMC Res Notes. 2016;9. doi:10.1186/s13104-016-1928-3

36. Corbett EL, Churchyard GJ, Hay M, Herselman P, Clayton T, Williams B, et al. The Impact of HIV Infection on Mycobacterium kansasii Disease in South African Gold Miners. Am J Respir Crit Care Med. 1999;160: 10–14. Available: www.atsjournals.org

37. Kone B, Sarro YS, Maiga M, Sanogo M, Somboro AM, Diarra B, et al. Clinical characteristics of non-tuberculous mycobacterial pulmonary infections in Bamako, Mali. Epidemiol Infect. 2018;146: 354–358. doi:10.1017/S0950268817003090

38. Jankovic M, Samarzija M, Sabol I, Jakopovic M, Katalinic Jankovic V, Zmak L, et al. Geographical distribution and clinical relevance of non-tuberculous mycobacteria in Croatia. International Journal of Tuberculosis and Lung Disease. 2013;17: 836–841. doi:10.5588/ijtld.12.0843

39. Sun Q, Yan J, Liao X, Wang C, Wang C, Jiang G, et al. Trends and Species Diversity of Non-tuberculous Mycobacteria Isolated From Respiratory Samples in Northern China, 2014–2021. Front Public Health. 2022;10: 1–8. doi:10.3389/fpubh.2022.923968

40. Farnia P, Farnia P, Ghanavi J, Velayati AA. Epidemiological Distribution of Nontuberculous Mycobacteria Using Geographical Information System. Nontuberculous Mycobacteria (NTM): Microbiological, Clinical and Geographical Distribution. Elsevier; 2019. pp. 191–321. doi:10.1016/B978-0-12-814692-7.00010-3

41. Gonzalo-Asensio J, Pérez I, Aguiló N, Uranga S, Picó A, Lampreave C, et al. New insights into the transposition mechanisms of IS6110 and its dynamic distribution between Mycobacterium tuberculosis Complex lineages. PLoS Genet. 2018;14. doi:10.1371/journal.pgen.1007282

42. Lira LAS, Santos FCF, Carvalho MSZ, Montenegro RA, Lima JFC, Schindler HC, et al. Evaluation of a IS6110-Taqman real-time PCR assay to detect Mycobacterium tuberculosis in sputum samples of patients with pulmonary TB. J Appl Microbiol. 2013;114: 1103–1108. doi:10.1111/jam.12119

43. Hemeg HA, Albulushi HO, Ozbak HA, Ali HM, Alahmadi EK, Almutawif YA, et al. Evaluating the Sensitivity of Different Molecular Techniques for Detecting Mycobacterium tuberculosis Complex in Patients with Pulmonary Infection. Pol J Microbiol. 2023;72: 421–431. doi:10.33073/pjm-2023-040

44. Hanifa Y, Fielding KL, Chihota VN, Adonis L, Charalambous S, Foster N, et al. The utility of repeat Xpert MTB/RIF testing to diagnose tuberculosis in HIV-positive adults with initial negative result. Gates Open Res. 2018;2. doi:10.12688/gatesopenres.12815.1

45. Kolia-Diafouka P, Carrère-Kremer S, Lounnas M, Bourdin A, Kremer L, Van de Perre P, et al. Detection of Mycobacterium tuberculosis in paucibacillary sputum: performances of the Xpert MTB/RIF ultra compared to the Xpert MTB/RIF, and IS6110 PCR. Diagn Microbiol Infect Dis. 2019;94: 365–370. doi:10.1016/j.diagmicrobio.2019.02.008

46. Hamdi A, Fida M, Deml SM, Abu Saleh O, Wengenack NL. Utility of Mycobacterium tuberculosis PCR in ruling out active disease and impact on isolation requirements in a low prevalence setting. J Clin Tuberc Other Mycobact Dis. 2020;21: 1–5. doi:10.1016/j.jctube.2020.100181

47. Fang Y, Wu WH, Pepper JL, Larsen JL, Marras SAE, Nelson EA, et al. Comparison of real-time, quantitative PCR with molecular beacons to nested PCR and culture methods for detection of Mycobacterium avium subsp. paratuberculosis in bovine fecal samples. J Clin Microbiol. 2002;40: 287–291. doi:10.1128/JCM.40.1.287-291.2002

48. Seo AN, Park HJ, Lee HS, Park JO, Chang HE, Nam KH, et al. Performance characteristics of nested polymerase chain reaction vs real-time polymerase chain reaction methods for detecting mycobacterium tuberculosis complex in paraffin-embedded human tissues. Am J Clin Pathol. 2014;142: 384–390. doi:10.1309/AJCP2QZRH4ZNPRDD

49. Dowling WB, Whitelaw A, Nel P. Tracing TB: Are there predictors for active TB disease in patients with Xpert Ultra trace results? International Journal of Infectious Diseases. 2022;114: 115–123. doi:10.1016/j.ijid.2021.10.056

50. Boyles TH, Hughes J, Cox V, Burton R, Meintjes G, Mendelson M. False-positive Xpert® MTB/RIF assays in previously treated patients: Need for caution in interpreting results. International Journal of Tuberculosis and Lung Disease. 2014;18: 876–878. doi:10.5588/ijtld.13.0853

Sequencing results for the identification of NTM species

Click here to access/download;Figure;Fig 2.tif

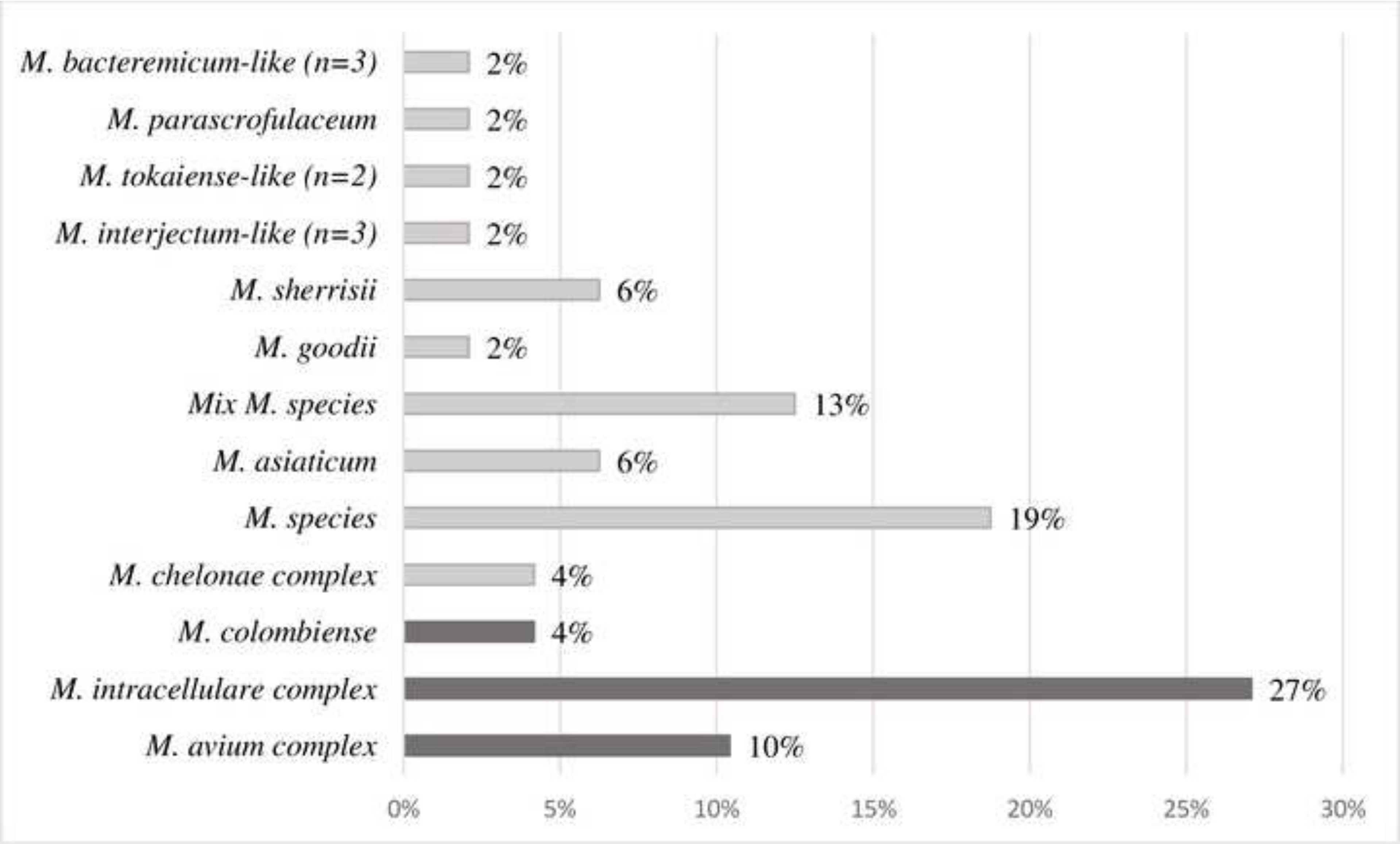

 Click here to access/download;Figure;Fig 1.tif

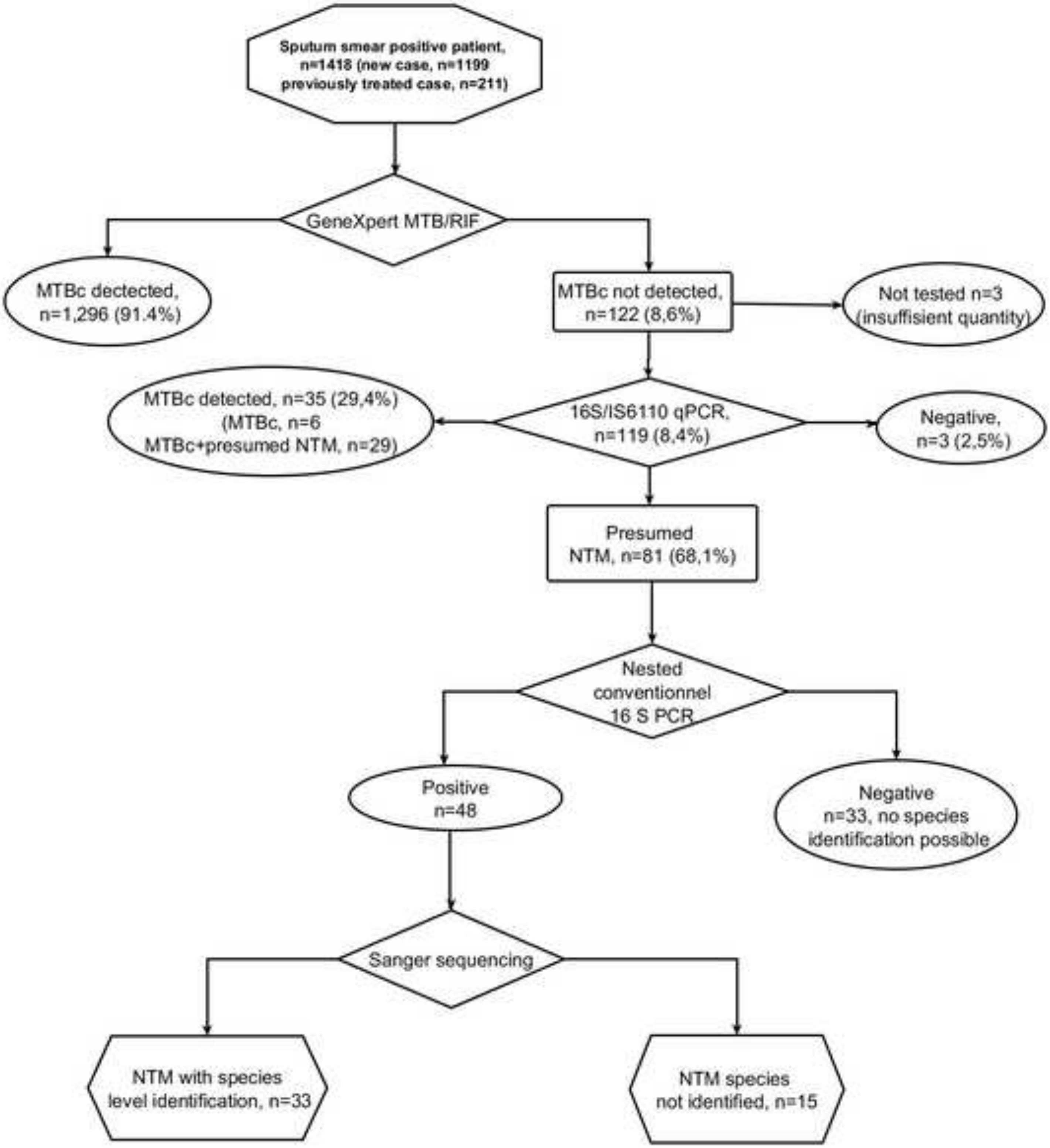

Minimal data set

Click here to access/download
Supporting Information
Minimal data set.csv

Minimal data set

Click here to access/download
Supporting Information
Minimal data set.mat

Prevalence of non-tuberculous mycobacteria among ~~people presumed to have tuberculosis, positive for~~ people with acid-fast ~~bacilli~~ positive ~~-~~presumptive tuberculosis ~~cases~~ in Mali

Prevalence of non-tuberculous mycobacteria among people with presumptive tuberculosis in Mali

Aissata Boubakar CISSE[1]*, Anna S. DEAN[2], Armand Van DEUN[3†], Jelle KEYSERS[3], Willem-Bram De RIJK~~E~~[3], Mourad GUMUSBOGA[3], Hawa SAMAKE[1], Seydou ARAMA[1], Bassirou DIARRA[5], Ibrahim DJILLA[1], Fatoumata N. COULIBALY[1], Hawa SIMPARA[1], Mamadou BERTHE[1], Khadidia OUATTARA[6], Yacouba TOLOBA[6], Ibrehima GUINDO[1], Bouke de JONG[3], Leen RIGOUTS[3,4].

1- National Institute of Public Health, Bamako, Mali
2- Word Health Organization, Geneva
3- Mycobacteriology Unit, Institute of Tropical Medicine, Antwerp, Belgium
4- Belgian Coordinated Collections of Microorganisms, Antwerp, Belgium
5- University Clinical Research Center, Bamako, Mali
6- University Teaching hospital of Point G, Bamako, Mali

† Deceased in September 2023

* Corresponding author

E-mail: aissatacisse@yahoo.fr (ABC)

Formatted: Line spacing: Double

Formatted: English (United States)

Formatted: English (United States)

Formatted: English (United States)

Field Code Changed

Formatted: English (United States)

Abstract

Background: Non-tuberculous~~is~~ mycobacteria (NTM~~s~~) are environmental agents that can cause opportunistic pulmonary disease in humans and animals which is often misdiagnosed as tuberculosis (TB). In this study, we describe the cases of NTM~~s~~ identified during the first national anti-TB drug ~~-~~resistance survey conducted in Mali, and explore associated risk factors.

Methods: Sputum was collected from people presenting for pulmonary TB diagnosis, from April to December 2019, regardless of age. Microscopy-positive patients were enrolled and were tested by ~~GeneXpert~~ the Xpert MTB/RIF assay. ~~Cases that~~Patients who tested negative for the *Mycobacterium tuberculosis* complex (MTBc) were tested for the presence of mycobacteria by amplification of the IS*6110* and 16SrRNA (16S) genes through double quantitative real-time PCR, followed by nested PCR and Sanger sequencing of the IS*6110*-negative samples for NTM species identification.

Results: A total of 1,418 sputum smear-positive patients were enrolled, including 1,199 new cases, 211 previously treated cases and 8 whose previous treatment history was unknown. Based on the results of ~~Gene~~Xpert MTB/RIF assay and in-house PCR methods, 1,331 (93.9%) patients were positive for MTBc, 48 (3.4%) for NTM~~s~~ and ~~for 39 (2.7%)~~ no species identification was possible for 39 (2.7%). Advanced age ~~to~~ of ~~(~~65 and over~~)~~ ~~(~~with an OR 8.8, (95% CI 2.3-33.2 and~~,~~ p=0.001) and previous TB treatment with an ~~(~~OR 3.4 (95% CI 1.2-9.6 and p=0.016) were the risk factors statistically associated with NTM detection. *M. avium complex* (MAC) was the predominant NTM species, detected in 20 cases.

Formatted: Font: 16 pt

Formatted: Space After: 0 pt

Formatted: Font: 16 pt

Formatted: Font: 16 pt, Bold

Formatted: Font: 16 pt, English (United States)

Formatted: English (United States)

Formatted: Font: Not Bold, English (United States)

Conclusion: Detection of NTM~~s~~ in people presumed to have TB is an ongoing challenge, confounding correct TB diagnosis. Concomitant use of microscopy and GeneXpert testing among at-risk individuals could ~~lessen confusion~~improve patient management.

1. Introduction

The *Mycobacterium* genus groups together species of the *Mycobacterium tuberculosis* complex (MTBc) responsible for tuberculosis (TB) in humans and animals, *M. leprae*, the causative agent of leprosy, and ~~finally,~~ the species of mycobacteria commonly known as atypical or non-tuberculous ~~sis or~~ mycobacteria (NTM), including *M. ulcerans* responsible for Buruli ulcer. Unlike members of the MTBc group, NTM are not obligatory parasites of humans, yet~~but~~ ~~are~~ normal residents of soil and water, and ~~are also~~can be found in natural and treated water sources [1]~~[6]~~. More than 200 species of NTM have been officially recognised [2]~~[13]~~, of which around 25 are known to be strongly associated with disease in humans. Some species have been associated with pulmonary diseases causing symptoms similar to TB [1]~~[5]~~. Because of their habitat, humans are exposed to these bacteria on a daily basis, and ~~an~~ NTM disease must therefore be distinguished from a simple colonisation or contamination of clinical samples, for example ~~by~~ from tap water [1,3]~~[6,14]~~. ~~NTMs are recognised as environmental bacteria(1–4), yet some species have been associated with pulmonary diseases with symptoms similar to TB (5).~~ Unlike for ~~tuberculosis~~TB, the global epidemiology of disease~~s~~ caused by NTM is not well known. ~~They~~NTM isolation from clinical specimens ~~are~~is ~~much~~ mo~~re~~stly ~~frequently~~ reported in industrialised countries, with variable prevalence and incidence. ~~ranging from 1.0 to 1.8 cases per 100,000 More R~~ Studies ~~have~~ based on pulmonary specimen isolates, reported a prevalence ~~based on pulmonary isolates ranging from~~of 1.4 to 6.6 per 100,000 persons in the USA from

Formatted: Font: 16 pt, English (United States)

Formatted: Font: Not Italic

Formatted: Font: Not Italic

2004 to 2006 [4], 9.8 in 2010 in Ontario, Canada [5] and 5.8 per 100,000 in Germany in 2020 [6]. Incidence of 6.1 per 100,000 in 2012 in England [7] and 5.3 per 100, 000 in Germany [6] in 2020 have also reported ~~, with a similar prevalence of 5.8/100,000 in Germany~~[6]~~[10], while a much higher ps and Germany~~ .~~—~~In TB endemic countries, NTM are being reported less frequently and mainly in populations at-risk, particularly people with predisposing conditions or compromised immunity [8]~~[12]~~. However, experiences ~~tir~~ –in industrialised countries have shown that a declining ~~-countries where the~~ TB burden has also resulted in an increase in the number of NTM cases detected. With the strengthening of TB programmes in other setting, we may also anticipate a similar scenario, posing a growing diagnosis and clinical challenge in low- and middle-income countries ~~is gradually declining as a result of TBmeasures~~ medical ~~diagnostic and clinical~~ [9].

~~In countries where tuberculosis is endemic, they have been reported less frequently and have been reported mainly in populations at risk, especially people with predisposing conditions or compromised immunity.~~ The diagnosis of NTM lung disease is based on clinical, radiological and microbiological criteria [1]. Laboratory based detection of NTM, ~~their~~with ~~to~~ differentiation from Me ~~between tuberculosis~~TBc and ~~NTM disease and to~~ identification to the ~~y the~~ species level, ~~of NTM is~~ ~~are~~is largely ~~still not~~ inaccessible in most resource-limited countries ~~with limited resources~~. Microscopy, the most accessible technique ~~in countries with limited resources~~, ~~can detect~~identifies both MTBc~~tuberculous~~ and ~~non-tuberculous mycobacteria~~ NTM as acid-fast bacilli (AFB)~~after specific staining~~, but ~~cannot~~fails to distinguish~~fferentiate between~~ them. Since 2010~~As the performance of this technique is limited~~, the World Health Organization (WHO) recommends ~~that it be replaced by the~~rapid molecular tests such as GeneXpert MTB/RIF (Xpert) as the initial test for TB diagnosis, ~~which is more~~exhibiting a higher sensitiveity and ~~more~~

specificity [10]. Typically, theyThis assay Xpert can only identifiesy the presence of anMTBc species contained in the sample. A negative result for MTBc by GeneXpert MTB/RIF when obtained on AFB-positive sputum positive for AFB canould therefore presume presence ofbe indicative of anan infection with MNNTM [11]-. All mycobacteria are acid-fast bacilli (AFB) detectable by microscopy after specific staining, yet an AFB-positive result by sputum smear cannot specify the species. A negative result for the MTBc by GeneXpert MTB/RIF (Xpert hereafter) when performed on AFB-positive sputum can suggest an NTM infection (6). Cases of NTM infection in Mali were described before the introduction of Xpert into the TB diagnostic algorithm, particularly in patients who have been previously treated for TB.-In Mali, like in other countries, cases of NTM infection have been described, particularly in patients under investigation for failure of anti-tuberculosisTB treatment or who have relapsed after TB cure [12](7). FollowingWith the introduction of Xpert in the country, presumptiveplausible cases of NTM infection with AFB-on the basis of a positive smear and a negative Xpert have been were reported more frequently reported[13].

Unlike members of the MTBc, NTMs are not obligate parasites of humans. They are normal residents of soil and water. Over 170 species of NTM have been officially recognized(8), of which only 25 species have been strongly associated with disease in humans. Because their habitat exposes humans to these bacteria every day, the diagnosis of NTM disease must be differentiated from simple colonization or contamination of clinical specimens, for example by tap water. Identification to the species level is also important as treatment differs from one NTM species to another.

In this articlestudy, we describe the proportion of MTBc and of NTM detected in patients undergoing presenting for initial or recurrentpreviously treated TB diagnosistreatment or

~~retreatment for TB~~ in Mali, in the context of a nationwide drug ~~-~~resistance survey [14]~~[20]~~.~~.~~ We also explored~~looked for~~ risk factors associated with NTM detection.

2. Methods

2.1. Design of the study

~~The study was conducted f~~From April to December 2019 c~~in the context of the first national TB drug-resistance molecular survey carried out in Mali~~ [14]~~(9). C~~onsecutive eligible patients were recruited from 78 of 86 TB diagnostic laboratories in the country, representing 91% of the country's TB laboratory network. Eight l~~L~~aboratories ~~ that~~ notified less than 10 new bacteriologically confirmed pulmonary TB cases in 2017 and were ~~excluded~~ not included for logistic reasons. Eligible participants were defined as people undergoing evaluation for pulmonary TB, both with and without a history of prior treatment, regardless of age. The sample size of 994 new cases was calculated to meet the primary objective of the survey for measuring the percentage of new pulmonary TB cases resistant to rifampicin, for which a recruitment period of 4 months was estimated to be required [15] ~~(10)~~.

2.2. Sample collection and patient enrolment

Two sputum samples, each of approximately 5 mL, were ~~initially~~ collected from each patient in the participating laboratories. Smears were made directly from freshly collected sputum, stained according to the acid-fast staining technique used at the center (auramine for most sites and Ziehl-Neelsen for several small centers), and in line~~following~~ with international guidelines for diagnosing TB by microscopy [16] ~~(11)~~. In case one of these two sputum samples was AFB

positive, the patient was included in the drug ~~-~~resistance survey after giving informed consent, and an additional sample was collected.

The nurse completed the study questionnaire with the recruited patient. The questionnaire covered socio-demographic data, and information on previous TB treatment and pulmonary disease history (duration and chronicity of respiratory signs). ~~At the same time, the ddisease and the~~ Relevant clinical and treatment-related ~~conditions~~information ~~weare~~was also explained~~described~~ to the patient. All enrolled participants received anti-TB treatment. New patients ~~if he is new and he~~ were started on~~s the1~~first-line TB treatment on the basis of an AFB microscopy. For ~~recurrent~~ patients with previously treated TB~~recurrent disease~~, ~~already treated~~ in accordance with national guidelines, ~~re-treatment or RR-TB treatment was~~treatment was initiated based on the result of Xpert~~'s rifampicin susceptibility~~ MTB/RIF~~result~~ testing~~the GeneXpert result.s are awaited in order to rule out RR -TB before re-treatment.~~

2.3. Initial ~~Xpert~~ GeneXpert testing and specimen storage

The first two sputum samples collected were preserved in 5~~-~~mL of OMNIgene® ~~s~~SPUTUM (OM-S) decontamination reagent (DNA Genotek Inc, Canada) according to the manufacturer's recommendations [17]~~(12)~~, while the third sample was preserved in 10 mL of 96% ethanol. The conditioned samples were transported by public transport at ambient temperature in triple packaging to the National ~~Tuberculosis~~TB Reference Laboratory (NTRL) in Bamako.

~~To detect MTBc, the Gene~~The Xpert MTB/RIF assay (Cepheid, CA, USA) was performed at the ~~National Tuberculosis Reference Laboratory~~NTRL~~,~~ using 2mL of one OM~~NIgene~~S-preserved ~~the~~ sample ~~preserved in Omnigene~~, in ~~accordance~~ line with the manufacturer's recommendations (Cepheid and Genotek).

Formatted: Superscript

Leftovers of the OM-~~NIgene~~S-preserved samples were then stored in cryotubes in the form of sediment obtained after centrifugation at 4000g for 15 minutes, and mixed with 96% alcohol at an equal volume (0.5 mL sediment and 0.5 mL ethanol).

2.4. Molecular detection and identification of NTM species

~~For molecular detection and identification of NTM species, AFB positive/MTBc negative samples were transported at ambient temperature to the WHO Supranational Tuberculosis Reference Laboratory at the Institute of Tropical Medicine (ITM) in Antwerp, Belgium.~~

For molecular detection and identification of NTM species, according to the study protocol, ~~the~~ samples ~~of~~ from the following patients ~~,~~ were transported at ambient temperature to the WHO Supranational ~~Tuberculosis~~TB Reference Laboratory at the Institute of Tropical Medicine (ITM) in Antwerp, Belgium:

- cases with rifampicin resistance by Xpert MTB/RIF, regardless of anti-~~tuberculosis~~ TB treatment history;
- -all previously treated cases, regardless of Xpert MTB/RIF test result;
- and cases for whom MTBc was not detected by Xpert MTB/RIF~~.~~

In this article, we described only the employed molecular methods and obtained~~the~~ results for ~~of~~ species identification of probable NTM.~~ and molecular methods used for.~~ .

Upon arrival at ITM, ethanol-preserved samples were centrifuged at 4000 g for 15 minutes, digested by overnight proteinase K incubation, followed by lysis using an internal lysis buffer, after which DNA was extracted using the Modified Maxwell-LEV® automated extraction method (Promega, USA) [18]~~(13)~~ .

Formatted: Space After: 0 pt

Formatted: Space After: 0 pt, Line spacing: Double

Formatted: Normal, Line spacing: Double, No bullets or numbering

Formatted: Font: Times New Roman, 12 pt, Font color: Black

Formatted: Font: Times New Roman, 12 pt, Font color: Black, English (United Kingdom)

A duplex quantitative real-time polymerase chain reaction (qPCR) was performed, targeting the IS~~1~~*6110* gene for the detection of the MTBc and the 16S rRNA gene to detect all species of the *Mycobacterium* genus. The primers and probes used are presented in Table 1, while interpretation of results is depicted in Table 2.~~.~~

Formatted: Justified, Line spacing: Double

Table 1. Primers and probes for qPCR to simultaneously detect *Mycobacterium tuberculosis* complex and all mycobacterial species.

Target	Name	Sequences ~~primers and probes~~
**IS*6110* ~~qPCR~~**	~~Myco_Genus_S_v3~~	~~5'-ATGCAAGTCGAACGGAAAGG-3'~~
	~~Myco_Genus_AS_v2~~	~~5'-TCTGCCCGTATCGCC-C-3'~~
	IS*6110*_S	5'-CCTGAAAGACGTTATCCACCATAC-3'
	IS*6110*_AS	5'-GGC-TAG-TGC-ATT-GTC-ATA-GGA-G-3'
	~~Myco_Genus_P_v2~~	~~FAM-CAA-AC(lnC)-AC(lnC)-TA(lnC)-GA(lnG)-CTC-EDQ~~
	IS6110_P_DFO	DFO-TCT-CAG-TA(lnC)-AC(lnA)-TC(lnG)-ATC-CGG-T-BHQ2
**16S rRNA qPCR**	Myco_Genus_S_v3	5'-ATGCAAGTCGAACGGAAAGG-3'
	Myco_Genus_AS_v2	5'-TCTGCCCGTATCGCC-C-3'
	Myco_Genus_P_v2	FAM-CAA-AC(lnC)-AC(lnC)-TA(lnC)-GA(lnG)-CTC-EDQ

Formatted: Normal

Formatted: Font: Italic

Formatted: Font: Italic

Formatted: Font: Italic

Table 2. Interpretation of duplex IS*6110*-16SrRNA qPCR results

**16S qPCR Cycle threshold (Ct) ~~16S rRNA~~**	**~~qPCR~~ IS*6110* qPCR result**	
	**Positive**	**Negative**
**Positive with any Ct value**	-	No MTBc
**Positive and ≥IS*6110* Ct**	MTBc	
**Positive and <IS*6110* Ct**	MTBc + NTM	
**Negative**	MTBc<br>(low bacillary load)	No mycobacteria species<br>identification possible

MTBc = *Mycobacterium tuberculosis* complex; NTM = non-tuberculous mycobacteria: Ct = cycle threshold

Formatted: Font: Not Italic

Formatted: Font: Not Italic

Formatted: Font: Not Italic

Formatted: Font: Not Italic

Subsequently, a nested conventional PCR targeting the 16S rRNA gene was performed on samples identified as NTM by the duplex qPCR. The primers and probes used were as follows:

- P1 (first PCR): 5’-TGCTTAACACATGCAAGTCG-3’
- P2 new (first PCR): 5’-TCTCTAGACGCGTCCTGTGC-3’
- P7 (nested PCR): 5’-CATGCAAGTCGAACGGAAAGG-3’
- P16 new (nested PCR): 5’-AAGCCGTGAGATTTCACGAACA3’

Amplicons that were clearly positive on the agarose gel underwent Sanger sequencing. A negative PCR result was interpreted as the absence of MTBc or NTM.

Sequencing was subcontracted to BaseClear (BE Leiden, The Netherlands). The results obtained after entering the sequence into the Basic Local Alignment Search Tool (BLAST), of the National Centre for Biotechnology Information (NCBI) were interpreted as follows:

- If a sequence match was found on the NCBI, the NTM species was identified.
  - In case of 100% similarity (no mismatches), the species name was assigned.
  - In case 1-3 bp mismatches with the closest species, the “species-like” name was assigned
- If no match or >3 bp difference with the closest species was found on NCBI, the result was interpreted as *Mycobacterium* species.
- If there were overlapping peaks in the sequence, indicating the presence of several NTMs, the result was interpreted as "Mixture of NTMs ".

2.5. Data analysis

The data collected using the survey forms and sample submission forms were entered into EpiInfo version 7.2.5.0. The variables analysed were age, sex, region of residence, history of illness and history of anti-TB treatment. Percentages, medians and means were calculated by crosstab, and p-values and odds ratios with 95% confidence intervals were generated using regression analysis in IBM SPSS Statistics version 21X64 (IBM Corp., NY, USA).

2.6. Ethical considerations

~~3.~~ The drug ~~-~~resistance survey protocol in which a secondary analysis of samples is described in the informed consent was accepted by the ethics committee of the National Institute of Public Health Research by decision No. 08/2018/CE-INRSP on March 21, 2018. The objectives and expected results of the study were clearly explained in the language understood by the patient. Patients ~~have also been~~were asked for permission to use their samples for ~~further~~additional analys~~e~~is, including outside Mali. ~~both inside and outside the country in Mali and abroad., and~~ Oral and written consent~~s~~ were obtained from patients over 18 years of age. For patients under the age of 18, after obtaining the prior oral and written consent~~s~~ of the parent or companion, the child's assent to participate in the study was obtained in the presence of a witness other than the parent or companion.

Formatted: Font: Not Bold, English (United States)

~~4.~~3. Results

~~4.1.~~3.1. Mycobacteria detected

A total of 1,418 ~~sputum~~ AFB-positive ~~patients~~ people with presumed pulmonary TB were enrolled in the drug-resistance survey, including 1,199 new cases, 211 previously treated cases and 8 whose previous treatment history was unknown (Figure 1). Based on Xpert MTB/RIF results, 1,296 (91.4%) patients were positive for MTc, while 1~~22 (8.6%) had an~~ "MTBc was not detected for 22 (8.6%)." ~~result~~~~remained negative.~~

The duplex IS*6110*-16S qPCR performed for 119 of these 122 patients ~~with an Xpert result,~~ identified another 35 MTBc, bringing ~~of "MTB not detected" increased~~ the total number of patients with MTBc to 1,331 (93.9%). O~~n~~f these 35 additional cases ~~(29.4%)~~, MTBc alone was detected for ~~6~~six, while ~~and~~ 29 carr~~y~~ied~~ng~~ probable mixtures of MTBc and NTM.

Fig 1. Sample and analysis flow with summary of results, *MTBc = Mycobacterium tuberculosis complex; n= number; NTM = ~~N~~non ~~T~~tuberculous ~~M~~mycobacteria*

~~The duplex IS*6110*-16S rRNA qPCR was performed for 119 patients with an Xpert result of "MTB not detected" by which increase the number of patients with MTBc increased to 1331 (93.9%) through 35 additional cases (29.4%), for six of whom MTBc alone was detected and 29 carrying probable mixtures of MTBc and NTMs,~~ For 81 (68,1%) of 119 patients tested, only the 16S target reacted positive (presumed NTM), while the PCR remained negative for both targets in 3 (2,5%) patients. The nested conventional 16S PCR performed for the 81 presumed NTM detected by IS*6110*-16SrRNA qPCR, was positive for 48 or 3.4% (48/1418) of all enrolled patients, of which 33 could be identified to the species level by sequencing. The remaining 33 of

Formatted: Space After: 0 pt

Formatted: Justified, Space After: 10 pt, Line spacing: Double

Formatted: Font: Times New Roman, 12 pt, Italic, Font color: Text 2

Formatted: English (United States)

81 presumed NTM, had a negative nested PCR result, thus no sequencing could be performed (Fig 1).

Thus, ~~For another 33 of 81 the nested PCR remained negative, thus no sequencing could be performed (Fig 1).~~~~Sequencing identified~~ eleven species or groups of species could be identified, with predominance of the *M. avium* complex (MAC) representing 20 cases or 42% of all species detected, *M. intracellulare* complex in 13 patients (27%), *M. avium* in five (10%) and *M. colombiense* in two (4 %) (Fig 2). ~~The remaining 33 of 81 presumed NTM, had a negative nested PCR result, thus no sequencing could be performed (Fig 1).~~

Fig 2. Sequencing results for the identification of NTM species

Formatted: Left, Space After: 0 pt

Sequencing identified eleven species or groups of species, with predominance of the *M. avium* complex (MAC) representing 20 cases or 42% of all species detected, *M. intracellulare* complex in 13 patients (27%), *M. avium* in five (10%) and *M. colombiense* in two (4 %) (Fig 2).

4.2.3.2. Detection of NTM in relation to smear-microscopy results

The quantification of AFB was notified for 1414 of 1418 patients by the participating laboratories. A significantly higher proportion of NTM was detected in patients with lower AFB-grades: 7% for 1+ and scanty (Table 3), compared to 2% among 3+ patients (respectively OR 4.065, 95% CI: 1.876-8.808, p=0.000 and OR 4.3, 95% CI: 2.012-9.189, p=0.000). The difference was not significant compared to grade 2+ samples (p=0.462).

Table 3. Proportion of NTM and MTB detection in relation to quantitative sputum-smear microscopy results

Final sputum smear result	MTBc n (%)	NTM n (%)	No species identification possible -n (%)	Total n (%)
Rares BAARScanty	167 (83%)	13 (7%)	21 (10%)	201
1+	170 (89%)	14 (7%)	8 (4%)	192
2+	259 (94%)	7 (3%)	8 (3%)	274
3+	731 (98%)	14 (2%)	2 (0%)	747

**Not quantified**	0	0	4 (100)	4
**Total**	**1327 (93.86%)**	**48 (3.4%)**	**39 43 (2.83%)**	**1418 (100)**

MTBc = *M. tuberculosis* complex; NTM = non-tuberculous mycobacteria; n =number

Formatted: Font: Not Italic

Formatted: Font: Not Italic

Formatted: Font: Not Italic

~~4.3.~~3.3. Socio-demographic, clinical characteristics and risk factors for NTM infection

Male patients were more ~~abundant~~ common ~~numerous~~ among ~~all~~ study participants and among those with identified NTM, with a sex ratio of respectively 2.1 and 1.5 when compared to women, ~~but~~yet NTM presence was not associated with gender (Table 4). Contrary, older age was significantly associated with NTM detection. The median age was 37 years for all patients and 59.5 years for those ~~infected~~ with NTM detected. Compared to the under-34 age group, the 35-64 age group had a greater odds (OR 3.4, 95% CI: 1.1-10.3, p=0.03) of NTM detection, which further increased for the over-65 age group (OR 8.8, 95% CI: 2.3-33.2 p=0.001 (Table 4). Also, previously treated patients were more likely than new cases to have NTM~~s~~ in their sputum (OR 3.4, 95% CI: 1.2-9.6, p=0.016) (Table 4). We found no significant associations between NTM detection and HIV status or chronic respiratory signs. Regarding the patient’s residence, patients from the Mopti region were more likely to have NTM detected compared to Bamako residents (OR 4.1, 95% CI: 1.0-16.4, p=0.04). (Table 4).

Table 4. Multivariate logistic regression analyses of demographic and clinical variables as risk factors of NTM infection

Variables		Total patients n (%)	NTM detected n (%)	OR	p-value
**Gender n=1379**	Female	434 (31.5)	19 (39.6%)	0.8 [0.3-2.04]	0.7
	Male	945 (68.5)	29 (60.4%)		
**Age n=1379**	Median	37	**59,5**		
**Age group**	0-34 years (Reference)	603 (43.7)	4 (8.3)		
	35-64 years	659 (47.8)	29 (60.4)	**3.4 [1.1-10.3]**	**0.03**
	≥65 years	117 (8.5)	15 (31.3)	**8.8 [2.3-33.2]**	**0.001**
**History of treatment n=1371**	New	1165 (84.5)	30 (62.5)		
	Previously treatment	206 (14.9)	18 (37.5)	**3.4 [1.2-9.6]**	**0.016**
**HIV status n=1092**	Positive	77 (5.6)	4 (8.3)	2.8 [0.8-8.9]	0.07
	Negative	1015 (73.6)	29 (60.4)		
**Chronic clinical signs n=1174**	Yes	442 (37.6)	24 (60)	0.7 [0.3-1.8]	0.5
	No	752 (62.4)	16 (40)		
**Region of residence at the time of diagnosis n=1348**	Bamako (Reference)	451 (33.5)	5 (10.9)		
	Kayes	101 (7.5)	4 (8.7)	2.7 [0.5-12.7]	0.2
	Koulikoro	217 (16.1)	7 (15.2)	1.5 [0.3-7.4]	0.5
	Sikasso	155 (11.5)	12 (26.1)	2.9 [0.7-10.8]	0.10
	Ségou	147 (10.9)	7 (15.2)	3.2 [0.8-12.7]	0.09
	Mopti	191 (14.2)	7 (15.2)	**4.1 [1.02-16.4]**	**0.04**
	Northern regions	86 (6.4)	4 (8.7)	4.2 [0.7-25.2]	0.1

NTM = non-tuberculous mycobacteria; n = number; OR = odds ratio

Formatted: Left

~~5.~~4. Discussion

The proportion of sputum-AFB-positive patients and MTBc detected by Xpert MTB/RIF among people presumed to have TB in our study was slightly lower than the rate reported in other drug resistance surveys, ranging from 95.8% -97.7% [19–22] ~~[19,38–40].~~The confirmed proportion with NTM ~~detection rate~~ in our study was higher ~~(3.4%)~~ than ~~those obtained~~ in TB drug-resistance surveys ~~conducted in~~ Pakistan ~~with~~ (0.7%, ~~(~~13/1972) ~~NTM~~[19]~~,~~ and India ~~with~~ (1.~~19~~2%, ~~(~~35/2,938) ~~in India NTM~~ [23], yet lower than the 6.4% (317/4917) observed in China [24]~~-~~. This result may reflect regional differences in ~~NTM~~these mycobacteria occurrence and diseases they cause ~~d by NTMs~~ around the world~~-~~[25,26].

NTM detection was more likely among paucibacillary patients as determined by smear microscopy, corroborating findings from Nigeria where the AFB-smear grading was relatively lower for patients with NTM detected than for those with MTBc [27], and higher AFB grading was predictive for MTBc detection [28]. With age, the risk of developing NTM infection increased, but we did not find a significant association between gender and NTM infection, contrary to other studies, where females have been more frequently associated with NTM infections, especially at older ages [11,29,30]. Previous anti-TB treatment was another risk factor for NTM infection in our study, confirming findings from NTM pulmonary disease in China and South Africa [31,32]. Nevertheless, we did not find an association between NTM detection and chronic respiratory signs in our study, in contrast with the described association between NTM infection and the presence of lung disease or chronic respiratory symptoms in studies from China~~ese~~ and ~~Danish studies~~ Denmark [33,34].

HIV infection has frequently been identified as a risk factor for NTM infections and disease, especially in situations of advanced immunosuppression [27,35,36]. In our study, the trend

towards an association between HIV-positivity and NTM detection was not significant, corroborating previous observation in Mali [37]. In the context of the drug -resistance survey, no information on anti-retroviral treatment has beenwas collected, but such treatment is to be started upon HIV diagnosis as per national guidelines in Mali. While patients from the Mopti region had a higher odds for having NTM detected in their sputum than those from the Bamako region, further investigation of the environmental conditions in these regions would be needed, especially as NTM are environmental bacteria and have often been isolated at different frequencies in different geographical areas [25,30,38]. MAC species were the most commonly NTM detected in our survey. The predominance of MAC has been reported in pulmonary NTM infections in many countries around the world [25,26,39,40]. -In Mali, between 2004 and 2013, *M. avium* was the most frequently identified species of the MAC complex [12,37].

The rate of sputum -AFB-positive patients and MTBc detected by Xpert in our study was slightly lower than the rate reported in other drug-resistance surveys, ranging from 95.8% -97.7% [19-22](14-17).

The useEmployment of an additional in-house qPCR for IS*6110* in our study has allowed to increase the detection of MTBc. The MTBc-specific repetitive element IS*6110* is a suitable has been described as a marker target of choice for to employ in the detection of MTBc detection assays [41]. , hence its use in many tuberculosis detection techniques. The applied qPCR IS*6110* has shown good sensitivity and specificity for the detection of MTBc compared to culture [42](18) and higher performance when compared to the Xpert MTB/RIF assay [43].. The observed high Ct values in our study indicates, however, a low quantity of MTB DNA anyway forin samples from these patients, that may as well have become positivehave been positive upon repeated Xpert testing [44](19), or by using the Xpert Ultra test, which is more sensitive with use

Formatted: Font: Italic

Formatted: Space After: 0 pt

Formatted: Font: Italic

Formatted: English (United States)

~~of~~by targeting both IS*6110* and another repetitive element IS*1081* for MTBc detection, in addition to the *rpoB* gene for assessing rifampicin susceptibility [45]~~-~~. The higher positivity rate by qPCR compared to the conventional nested PCR we observed in our study, could be partially attributable to the non-specificity of the 16S primers employed for the in-house qPCR which also detects species belonging to closely related genera like *Rhodococcus* and *Nocardia*, hence potentially overestimating the NTM rate. These closely related species indeed can be detected in smear-positive samples not containing tubercle bacilli, such as documented in the USA with 78.4% (134/171) of AFB-positive specimens yielding a positive culture with NTM or *Nocardia* spp, while 14.6% (25/171) of AFB-positive specimens grew MTBc and another 7% (12/171) remained negative in culture [46]~~(20). The proportion of NTM versus *Nocardia* spp note the clinical relevance of these isolated bacteria were discussed in that study~~. In another study in Nigeria, out of 32 smear-positive samples identified as not containing ~~tuberculosis~~TB bacilli, ~~6~~ six were NTM (18.75%), 14 were ~~C~~corynebacteria (43.75%) and 12 ~~R~~rhodococci (37.5%) [28]~~(21)~~. In our study, we did not further identify the non-MTBc/non-NTM samples. In addition, the lower sensitivity of the conventional nested PCR may have hampered confirmation by sequencing in case of negative results or low amplicon yield. Indeed, the in-house qPCR has already shown good sensitivity, while limited loss of DNA has been described during multiple handling steps for conventional nested PCR[47,48]~~(22,23)~~. This lower sensitivity is reflected in the sample's~~'~~ AFB-grading, with less non-identified samples among the strong positive specimens.

This study was part of the ~~DR-TB~~national TB drug resistance survey~~-study~~, comprising both new and ~~recurrent TB~~previously treated cases. The concern of false-positive MTBc detection in sputum from recurrent patients has mostly been raised for the highly sensitive, newer generation

Formatted: Font: Italic

Formatted: Font: Italic

Formatted: Font: Italic

Formatted: Font: Times New Roman

Formatted: Font: Times New Roman

of rapid molecular assays, such as the “MTB detected Trace” result by Xpert Ultra [49,50]. Nevertheless, even smear -microscopy can still show ~~dead~~non-viable AFB, and introduction of alternative/additional diagnostic assays or flows would need to be validated against culture.

Our ~~DR-TB~~ survey was not designed to ~~perform~~ include culture in the diagnostic algorithm, and hence we could not assess- the viability of the detected MTBc and/or NTM bacilli, nor whether co-detection of MTBc and NTM reflect~~s~~ed simultaneous infection, or rather an ~~the~~ NTM infection following ~~the~~ MTBc infection in previously treated patients, which was still detectable ~~as~~due to remnant DNA.

~~The confirmed NTM detection rate in our study was higher (3.4%) than that obtained in TB drug resistance surveys conducted in Pakistan with 0.7% (13/1972) NTM (15,24), and in India with 1.19% (35/2,938) NTM (24), but it was lower than the 6.4% (317/4917) observed in China (25) . This result may reflect regional differences in NTM occurrence and disease caused by NTMs around the world (26,27).~~

~~NTM detection was more likely among paucibacillary patients as determined by smear microscopy, corroborating findings from Nigeria where the AFB smear grading was respectively lower for NTM cases than for MTBc patients (28) and was predictive for MTBc detection when it was higher (21)With age, the risk of developing NTMs infection increased, but we did not find a significant association between gender and NTM infection, contrary to other studies, where females have been more frequently associated with NTM infections, especially at older ages (6,29,30). Previous anti-TB treatment was another risk factor for NTM infection in our study, confirming findings from NTM pulmonary disease in China and South Africa (31,32). Nevertheless, we did not find an association between NTM detection and chronic respiratory~~

Formatted: Font: Times New Roman, 12 pt, Font color: Black, English (United States)

Formatted: Font: Times New Roman

Formatted: Space After: 0 pt

signs in our study, in contrast with the described association between NTM infection and the presence of lung disease or chronic respiratory symptoms in Chinese and Danish studies(33,34).

HIV infection has frequently been identified as a risk factor for NTM infections and disease, especially in situations of advanced immunosuppression (28,35,36). In our study the trend towards an association between HIV-positivity and NTM detection was not significant, corroborating previous observation in Mali (37,38). In the context of the drug-resistance survey, no information on anti-retroviral treatment has been collected, but such treatment is to be started upon HIV diagnosis as per national guidelines in Mali. While patients from the Mopti region had a higher odds for having NTM detected in their sputum than those from the Bamako region, further investigation of the environmental conditions in these regions would be needed, especially as NTMs are environmental bacteria and have often been isolated at different frequencies in different geographical areas(26,30,39). The MAC was the most common NTM detected. The predominance of MAC has been reported in pulmonary NTM infections in many countries around the world (26,27,40,41) 29,38,40,41. In Mali, between 2004 and 2013, *M. avium* was the most frequently identified species of the MAC complex (7,27,37).

In this studyAlso, we did not investigate whether the detected NTM were attributable to true infection, colonization or sample contamination. We could not assess the American Thoracic SocietyAST criterium to detect NTM in at least 3 different samples per patient given the design of the survey and the retrospective nature of molecular analysis to detect NTM. However, study participants were people with respiratory signs undergoing evaluation for pulmonary TB attending health centers or patients failing TB treatment. AlsoFinally, we do not describe the outcome of patients in whom NTM was detected, but all were started on 1st line TB therapy or multidrug-resistant treatment following the results of initial microscopy and Xpert testing.

~~6.~~5. Conclusion

Among sputum~~-~~ AFB-positive patients presenting in Mali, 3.4% were due to NTM. Particular attention must be paid to populations at risk, such as elderly patients, and patients with a history of previous treatment. TB prevention and care programmes in resource-limited countries should ensure that their routine diagnostic algorithms can detect such cases, based on the concurrent use of rapid molecular TB diagnostics and microscopy in the absence of other molecular assays to detect NTM. However, culture is still necessary to isolate these mycobacteria and to monitor~~follow up any~~ treatment in the event of confirmation of NTM disease. Clinical assessment and management of NTM infections~~ however~~, remain a challenge worldwide.

Acknowledgements

We would like to thank the ~~tuberculosis~~TB management sites for recruiting patients, ~~for~~ collecting and transporting samples, the country's regional health departments for regional coordination of the drug resistance study and Olga Tosas AUGUET for her assistance in planning the survey, monitoring the field activities and preliminary data analysis”.

Formatted: Font: Not Italic

Formatted: Font: Not Italic

References

1. Griffith DE, Aksamit T, Brown-Elliott BA, Catanzaro A, Daley C, Gordin F, et al. An official ATS/IDSA statement: Diagnosis, treatment, and prevention of nontuberculous mycobacterial diseases. Am J Respir Crit Care Med. 2007;175: 367–416. doi:10.1164/rccm.200604-571ST

2. Pascapurnama DN, Zavitri NG, Koesoemadinata RC, Cahyadi AI, Chaidir L. Identification of Significant Pathogenic Nontuberculous Mycobacteria Species from Presumptive TB Patients Using Partial hsp65 Gene Sequencing. Infect Drug Resist. 2023;16. doi:10.2147/IDR.S419956

3. Haworth CS, Banks J, Capstick T, Fisher AJ, Gorsuch T, Laurenson IF, et al. British Thoracic Society guidelines for the management of non-tuberculous mycobacterial pulmonary disease (NTM-PD). Thorax. 2017;72: iii1-ii64. doi:10.1136/thoraxjnl-2017-210927

4. Prevots DR, Shaw PA, Strickland D, Jackson LA, Raebel MA, Blosky MA, et al. Nontuberculous mycobacterial lung disease prevalence at four integrated health care delivery systems. Am J Respir Crit Care Med. 2010;182: 970–976. doi:10.1164/rccm.201002-0310OC

5. Marras TK, Mendelson D, Marchand-Austin A, May K, Jamieson FB. Pulmonary nontuberculous mycobacterial disease, Ontario, Canada, 1998-2010. Emerg Infect Dis. 2013;19: 1889–1891. doi:10.3201/eid1911.130737

6. Corbett C, Finger P, Heiß-Neumann M, Bohnert J, Eder IB, Eisele M, et al. Development of prevalence and incidence of non-tuberculous mycobacteria in German laboratories from 2016 to 2020. Emerg Microbes Infect. 2023;12. doi:10.1080/22221751.2023.2276342

7. Shah NM, Davidson JA, Anderson LF, Lalor MK, Kim J, Thomas HL, et al. Pulmonary Mycobacterium avium-intracellulare is the main driver of the rise in non-tuberculous mycobacteria incidence in England, Wales and Northern Ireland, 2007-2012. BMC Infect Dis. 2016;16. doi:10.1186/s12879-016-1521-3

8. Varma-Basil M, Bose M. Mapping the Footprints of Nontuberculous Mycobacteria. Nontuberculous Mycobacteria (NTM): Microbiological, Clinical and Geographical Distribution. Elsevier; 2019. pp. 155–175. doi:10.1016/B978-0-12-814692-7.00008-5

9. Iseman MD, Marras TK. The importance of nontuberculous mycobacterial lung disease. Am J Respir Crit Care Med. 2008;178: 999–1000. doi:10.1164/rccm.200808-1258ED

10. WHO. Module 3: Diagnosis WHO operational handbook on tuberculosis Rapid diagnostics for tuberculosis detection. 2021.

11. Kong L, Xie B, Liu Q, Hua L, Bhusal A, Bao C, et al. Application of acid-fast staining combined with GeneXpert MTB/RIF in the diagnosis of non-tuberculous mycobacteria

pulmonary disease. International Journal of Infectious Diseases. 2021;104: 711–717. doi:10.1016/j.ijid.2020.12.091

12. Maiga M, Siddiqui S, Diallo S, Diarra B, Traoré B, Shea YR, et al. Failure to recognize nontuberculous mycobacteria leads to misdiagnosis of chronic pulmonary tuberculosis. PLoS One. 2012;7: e36902. doi:10.1371/journal.pone.0036902

13. Toloba Y, Cissé AB, Soumaré D, Ouattara K, Kanouté T, Koné D, et al. Apport du GeneXpert dans le diagnostic de la tuberculose et de la résistance à la rifampicine au Mali après 22 mois d'acquisition. Rev Mal Respir. 2017;34: A216–A217. doi:10.1016/j.rmr.2016.10.515

14. World Health Organization. Global tuberculosis report 2020. World Health Organization; 2020.

15. World Health Organization. Guidelines for surveillance of drug resistance in tuberculosis 5 th Edition. 2015.

16. Lumb R, Van Deun A, Bastian I, Fitz-Gerald M, Pathology SA, Stop TB Partnership (World Health Organization). Laboratory diagnosis of tuberculosis by sputum microscopy : the handbook. 2013.

17. DNA Genotek Inc. How can I use OMNIgene®•SPUTUM? Use Case 4: Reflex testing: Microscopy centre with or without GeneXpert® centrifuge. 2016 [cited 4 Apr 2017]. Available: www.dnagenotek.com•

18. Corporation P. Maxwell(R) 16 Cell LEV DNA Purification Kit Technical Bulletin TB383. 2012 [cited 11 Mar 2024]. Available: www.promega.com

19. Tahseen S, Qadeer E, Khanzada FM, Rizvi AH, Dean A, Van Deun A, et al. Use of Xpert® MTB/RIF assay in the first national antituberculosis drug resistance survey in Pakistan. International Journal of Tuberculosis and Lung Disease. 2016;20: 448–455. doi:10.5588/ijtld.15.0645

20. Lopes C, Kundu D, Da Costa Barreto I, da Cruz B, Siva Kumar S, Ramalingam S, et al. Experience on the first national anti-TB drug resistance survey (DRS) in Timor-Leste. Glob Health Res Policy. 2022;7: 2–9. doi:10.1186/s41256-022-00249-z

21. Aia P, Kal M, Lavu E, John LN, Johnson K, Coulter C, et al. The burden of drug-resistant tuberculosis in Papua New Guinea: Results of a large population-based survey. PLoS One. 2016;11: 2–9. doi:10.1371/journal.pone.0149806

22. Iem V, Dean A, Zignol M, Vongvichit P, Inthavong D, Siphanthong S, et al. Low prevalence of MDR-TB in Lao PDR: results from the first national anti-tuberculosis drug resistance survey. Tropical Medicine and International Health. 2019;24: 421–431. doi:10.1111/tmi.13205

23. Sharma M, Malhotra B, Tiwari J, Bhargava S. Profile of Nontuberculous Mycobacteria in Patients Suspected of Tuberculosis and Drug-Resistant Tuberculosis. J Lab Physicians. 2020;12: 203–211. doi:10.1055/s-0040-1721160

24. Liu CF, Song YM, He WC, Liu DX, He P, Bao JJ, et al. Nontuberculous mycobacteria in China: incidence and antimicrobial resistance spectrum from a nationwide survey. Infect Dis Poverty. 2021;10. doi:10.1186/s40249-021-00844-1

Formatted: English (United States)

25. Hoefsloot W, Van Ingen J, Andrejak C, Ängeby K, Bauriaud R, Bemer P, et al. The geographic diversity of nontuberculous mycobacteria isolated from pulmonary samples: An NTM-NET collaborative study. European Respiratory Journal. 2013;42: 1604–1613. doi:10.1183/09031936.00149212

Formatted: English (United States)

Formatted: English (United States)

26. Okoi C, Anderson STB, Antonio M, Mulwa SN, Gehre F, Adetifa IMO. Non-tuberculous Mycobacteria isolated from Pulmonary samples in sub-Saharan Africa - A Systematic Review and Meta Analyses. Sci Rep. 2017;7. doi:10.1038/s41598-017-12175-z

Formatted: English (United States)

27. Aliyu G, El-Kamary SS, Abimiku A, Brown C, Tracy K, Hungerford L, et al. Prevalence of Non-Tuberculous Mycobacterial Infections among Tuberculosis Suspects in Nigeria. PLoS One. 2013;8: e63170. doi:10.1371/journal.pone.0063170

Formatted: English (United States)

28. Dinic L, Idigbe OE, Meloni S, Rawizza H, Akande P, Eisen G, et al. Sputum Smear Concentration May Misidentify Acid-Fast Bacilli As Mycobacterium Tuberculosis in HIV-Infected Patients. J Acquir Immune Defic Syndr. 2013;63: 168–177. doi:DOI:10.1097/QAI.0b013e31828983b9

Formatted: English (United States)

Formatted: English (United States)

29. Kennedy TP, Weber DJ. Nontuberculous mycobacteria. An underappreciated cause of geriatric lung disease. Am J Respir Crit Care Med. 1994;149: 1654–1658. doi:10.1164/ajrccm.149.6.8004325

Formatted: English (United States)

Formatted: English (United States)

30. Limo JJ, Nyerere A, Bii C. Prevalence, Species Characterization and factors associated with Non tuberculous mycobacteria among tuberculosis retreatment patients submitting samples to the National Tuberculosis Reference Laboratory, Kenya. Jomo Kenyatta University of Agriculture and Technology. 2016. Available: http://ir.jkuat.ac.ke

Formatted: English (United States)

31. Xu J, Li P, Zheng S, Shu W, Pang Y. Prevalence and risk factors of pulmonary nontuberculous mycobacterial infections in the Zhejiang Province of China. Epidemiol Infect. 2019;147: e269. doi:10.1017/S0950268819001626

Formatted: English (United States)

32. Corbett EL, Churchyard GJ, Clayton T, Herselman P, Williams B, Hayes R, et al. Risk Factors for Pulmonary Mycobacterial Disease in South African Gold Miners A Case-Control Study. Am J Respir Crit Care Med. 1999;159: 94–99. doi:https://doi.org/10.1164/ajrccm.159.1.9803048

Formatted: English (United States)

Formatted: English (United States)

33. Andréjak C, Nielsen R, Thomsen V, Duhaut P, Sørensen HT, Thomsen RW. Chronic respiratory disease, inhaled corticosteroids and risk of non-tuberculous mycobacteriosis. Thorax. 2013;68: 256–262. doi:10.1136/thoraxjnl-2012-201772

Formatted: English (United States)

Formatted: English (United States)

34. Feng JY, Chen WC, Chen YY, Su WJ. Clinical relevance and diagnosis of nontuberculous mycobacterial pulmonary disease in populations at risk. Journal of the Formosan Medical Association. 2020;119: S23–S31. doi:10.1016/j.jfma.2020.05.012

Formatted: English (United States)

Formatted: English (United States)

35. Hoza AS, Mfinanga SGM, Rodloff AC, Moser I, König B. Increased isolation of nontuberculous mycobacteria among TB suspects in Northeastern, Tanzania: Public health and diagnostic implications for control programmes. BMC Res Notes. 2016;9. doi:10.1186/s13104-016-1928-3

36. Corbett EL, Churchyard GJ, Hay M, Herselman P, Clayton T, Williams B, et al. The Impact of HIV Infection on Mycobacterium kansasii Disease in South African Gold Miners. Am J Respir Crit Care Med. 1999;160: 10–14. Available: www.atsjournals.org

37. Kone B, Sarro YS, Maiga M, Sanogo M, Somboro AM, Diarra B, et al. Clinical characteristics of non-tuberculous mycobacterial pulmonary infections in Bamako, Mali. Epidemiol Infect. 2018;146: 354–358. doi:10.1017/S0950268817003090

38. Jankovic M, Samarzija M, Sabol I, Jakopovic M, Katalinic Jankovic V, Zmak L, et al. Geographical distribution and clinical relevance of non-tuberculous mycobacteria in Croatia. International Journal of Tuberculosis and Lung Disease. 2013;17: 836–841. doi:10.5588/ijtld.12.0843

39. Sun Q, Yan J, Liao X, Wang C, Wang C, Jiang G, et al. Trends and Species Diversity of Non-tuberculous Mycobacteria Isolated From Respiratory Samples in Northern China, 2014–2021. Front Public Health. 2022;10: 1–8. doi:10.3389/fpubh.2022.923968

40. Farnia P, Farnia P, Ghanavi J, Velayati AA. Epidemiological Distribution of Nontuberculous Mycobacteria Using Geographical Information System. Nontuberculous Mycobacteria (NTM): Microbiological, Clinical and Geographical Distribution. Elsevier; 2019. pp. 191–321. doi:10.1016/B978-0-12-814692-7.00010-3

41. Gonzalo-Asensio J, Pérez I, Aguiló N, Uranga S, Picó A, Lampreave C, et al. New insights into the transposition mechanisms of IS6110 and its dynamic distribution between Mycobacterium tuberculosis Complex lineages. PLoS Genet. 2018;14. doi:10.1371/journal.pgen.1007282

42. Lira LAS, Santos FCF, Carvalho MSZ, Montenegro RA, Lima JFC, Schindler HC, et al. Evaluation of a IS6110-Taqman real-time PCR assay to detect Mycobacterium tuberculosis in sputum samples of patients with pulmonary TB. J Appl Microbiol. 2013;114: 1103–1108. doi:10.1111/jam.12119

43. Hemeg HA, Albulushi HO, Ozbak HA, Ali HM, Alahmadi EK, Almutawif YA, et al. Evaluating the Sensitivity of Different Molecular Techniques for Detecting Mycobacterium tuberculosis Complex in Patients with Pulmonary Infection. Pol J Microbiol. 2023;72: 421–431. doi:10.33073/pjm-2023-040

44. Hanifa Y, Fielding KL, Chihota VN, Adonis L, Charalambous S, Foster N, et al. The utility of repeat Xpert MTB/RIF testing to diagnose tuberculosis in HIV-positive adults with initial negative result. Gates Open Res. 2018;2. doi:10.12688/gatesopenres.12815.1

45. Kolia-Diafouka P, Carrère-Kremer S, Lounnas M, Bourdin A, Kremer L, Van de Perre P, et al. Detection of Mycobacterium tuberculosis in paucibacillary sputum: performances of

the Xpert MTB/RIF ultra compared to the Xpert MTB/RIF, and IS6110 PCR. Diagn Microbiol Infect Dis. 2019;94: 365–370. doi:10.1016/j.diagmicrobio.2019.02.008

46. Hamdi A, Fida M, Deml SM, Abu Saleh O, Wengenack NL. Utility of Mycobacterium tuberculosis PCR in ruling out active disease and impact on isolation requirements in a low prevalence setting. J Clin Tuberc Other Mycobact Dis. 2020;21: 1–5. doi:10.1016/j.jctube.2020.100181

47. Fang Y, Wu WH, Pepper JL, Larsen JL, Marras SAE, Nelson EA, et al. Comparison of real-time, quantitative PCR with molecular beacons to nested PCR and culture methods for detection of Mycobacterium avium subsp. paratuberculosis in bovine fecal samples. J Clin Microbiol. 2002;40: 287–291. doi:10.1128/JCM.40.1.287-291.2002

48. Seo AN, Park HJ, Lee HS, Park JO, Chang HE, Nam KH, et al. Performance characteristics of nested polymerase chain reaction vs real-time polymerase chain reaction methods for detecting mycobacterium tuberculosis complex in paraffin-embedded human tissues. Am J Clin Pathol. 2014;142: 384–390. doi:10.1309/AJCP2QZRH4ZNPRDD

49. Dowling WB, Whitelaw A, Nel P. Tracing TB: Are there predictors for active TB disease in patients with Xpert Ultra trace results? International Journal of Infectious Diseases. 2022;114: 115–123. doi:10.1016/j.ijid.2021.10.056

50. Boyles TH, Hughes J, Cox V, Burton R, Meintjes G, Mendelson M. False-positive Xpert® MTB/RIF assays in previously treated patients: Need for caution in interpreting results. International Journal of Tuberculosis and Lung Disease. 2014;18: 876–878. doi:10.5588/ijtld.13.0853

1. Claeys TA, Robinson RT. The many lives of nontuberculous mycobacteria. J Bacteriol. 2018;200(11).

2. Falkinham JO. Environmental sources of nontuberculous mycobacteria. Vol. 36, Clinics in Chest Medicine. 2015.

3. Falkinham JO. Ecology of nontuberculous mycobacteria-where do human infections come from? Semin Respir Crit Care Med. 2013;34(1):95–102.

4. Falkinham JO. Impact of human activities on the ecology of nontuberculous mycobacteria. Vol. 5, Future Microbiology. 2010.

5. American Thoracic Society. Diagnosis and treatment of disease caused by nontuberculous mycobacteria. Vol. 142, American Review of Respiratory Disease. 1990.

6. Kong L, Xie B, Liu Q, Hua L, Bhusal A, Bao C, et al. Application of acid-fast staining combined with GeneXpert MTB/RIF in the diagnosis of non-tuberculous mycobacteria pulmonary disease. International Journal of Infectious Diseases. 2021;104.

7. Maiga M, Siddiqui S, Diallo S, Diarra B, Traoré B, Shea YR, et al. Failure to recognize nontuberculous mycobacteria leads to misdiagnosis of chronic pulmonary tuberculosis. PLoS One. 2012 May 16;7(5).

Formatted: English (United States)

Formatted: English (United States)

Formatted: English (United States)

Formatted: English (United States)

Formatted: English (United States)

Formatted: English (United States)

Formatted: English (United States)

Formatted: English (United States)

Formatted: English (United States)

Formatted: English (United States)

8. Fedrizzi T, Meehan CJ, Grottola A, Giacobazzi E, Fregni Serpini G, Tagliazucchi S, et al. Genomic characterization of Nontuberculous Mycobacteria. Sci Rep. 2017;7.

9. World Health Organization. Global tuberculosis report 2020. World Health Organization; 2020.

10. World Health Organization. Guidelines for surveillance of drug resistance in tuberculosis 5 th Edition. 2015.

11. Lumb R, Van Deun A, Bastian I, Fitz-Gerald M, Pathology SA, Stop TB Partnership (World Health Organization). Laboratory diagnosis of tuberculosis by sputum microscopy : the handbook. 84 p.

12. How can I use OMNIgene®•SPUTUM? Use Case 4: Reflex testing: Microscopy centre with or without GeneXpert® centrifuge [Internet]. 2016. Available from: www.dnagenotek.com•

13. Corporation P. Maxwell(R) 16 Cell LEV DNA Purification Kit Technical Bulletin TB383 [Internet]. Available from: www.promega.com

14. Lopes C, Kundu D, Da Costa Barreto I, da Cruz B, Siva Kumar S, Ramalingam S, et al. Experience on the first national anti-TB drug resistance survey (DRS) in Timor-Leste. Glob Health Res Policy. 2022;7(1).

15. Tahseen S, Qadeer E, Khanzada FM, Rizvi AH, Dean A, Van Deun A, et al. Use of Xpert® MTB/RIF assay in the first national antituberculosis drug resistance survey in Pakistan. Vol. 20, International Journal of Tuberculosis and Lung Disease. International Union against Tubercul. and Lung Dis.; 2016. p. 448–55.

16. Aia P, Kal M, Lavu E, John LN, Johnson K, Coulter C, et al. The burden of drug-resistant tuberculosis in Papua New Guinea: Results of a large population-based survey. PLoS One. 2016;11(3).

17. Iem V, Dean A, Zignol M, Vongvichit P, Inthavong D, Siphanthong S, et al. Low prevalence of MDR-TB in Lao PDR: results from the first national anti-tuberculosis drug resistance survey. Tropical Medicine and International Health. 2019;24(4).

18. Lira LAS, Santos FCF, Carvalho MSZ, Montenegro RA, Lima JFC, Schindler HC, et al. Evaluation of a IS6110-Taqman real-time PCR assay to detect Mycobacterium tuberculosis in sputum samples of patients with pulmonary TB. J Appl Microbiol. 2013 Apr;114(4):1103–8.

19. Hanifa Y, Fielding KL, Chihota VN, Adonis L, Charalambous S, Foster N, et al. The utility of repeat Xpert MTB/RIF testing to diagnose tuberculosis in HIV-positive adults with initial negative result. Gates Open Res. 2018 Apr 26;2:22.

20. Hamdi A, Fida M, Deml SM, Abu Saleh O, Wengenack NL. Utility of Mycobacterium tuberculosis PCR in ruling out active disease and impact on isolation requirements in a low prevalence setting. J Clin Tuberc Other Mycobact Dis. 2020 Dec 1;21.

21. Dinic L, Idigbe OE, Meloni S, Rawizza H, Akande P, Eisen G, et al. Sputum Smear Concentration May Misidentify Acid-Fast Bacilli As Mycobacterium Tuberculosis in HIV-Infected Patients [Internet]. 2013. Available from: www.jaids.com

Formatted: French (France)

22. Fang Y, Wu WH, Pepper JL, Larsen JL, Marras SAE, Nelson EA, et al. Comparison of real-time, quantitative PCR with molecular beacons to nested PCR and culture methods for detection of Mycobacterium avium subsp. paratuberculosis in bovine fecal samples. J Clin Microbiol. 2002;40(1):287–91.

23. Seo AN, Park HJ, Lee HS, Park JO, Chang HE, Nam KH, et al. Performance characteristics of nested polymerase chain reaction vs real-time polymerase chain reaction methods for detecting mycobacterium tuberculosis complex in paraffin-embedded human tissues. Am J Clin Pathol. 2014 Sep 1;142(3):384–90.

24. Sharma M, Malhotra B, Tiwari J, Bhargava S. Profile of Nontuberculous Mycobacteria in Patients Suspected of Tuberculosis and Drug-Resistant Tuberculosis. J Lab Physicians. 2020;12(03).

25. Liu CF, Song YM, He WC, Liu DX, He P, Bao JJ, et al. Nontuberculous mycobacteria in China: incidence and antimicrobial resistance spectrum from a nationwide survey. Infect Dis Poverty. 2021 Dec 1;10(1).

26. Hoefsloot W, Van Ingen J, Andrejak C, Ängeby K, Bauriaud R, Bemer P, et al. The geographic diversity of nontuberculous mycobacteria isolated from pulmonary samples: An NTM-NET collaborative study. European Respiratory Journal. 2013 Dec 1;42(6):1604–13.

27. Okoi C, Anderson STB, Antonio M, Mulwa SN, Gehre F, Adetifa IMO. Non-tuberculous Mycobacteria isolated from Pulmonary samples in sub-Saharan Africa - A Systematic Review and Meta Analyses. Sci Rep. 2017;7(1).

28. Aliyu G, El-Kamary SS, Abimiku A, Brown C, Tracy K, Hungerford L, et al. Prevalence of Non-Tuberculous Mycobacterial Infections among Tuberculosis Suspects in Nigeria. PLoS One. 2013 May 9;8(5).

29. Kennedy TP, Weber DJ. Nontuberculous mycobacteria. An underappreciated cause of geriatric lung disease. Am J Respir Crit Care Med. 1994 Jun;149(6):1654–8.

30. Limo JJ, Nyerere A, Bii C. Prevalence, Species Characterization and factors associated with Non tuberculous mycobacteria among tuberculosis retreatment patients submitting samples to the National Tuberuculosis Reference Laboratory, Kenya. Vol. 7, Jomo Kenyatta University of Agriculture and Technology. 2016.

31. Xu J, Li P, Zheng S, Shu W, Pang Y. Prevalence and risk factors of pulmonary nontuberculous mycobacterial infections in the Zhejiang Province of China. Epidemiol Infect. 2019;147.

32. Corbett EL, Churchyard GJ, Clayton T, Herselman P, Williams B, Hayes R, et al. Risk Factors for Pulmonary Mycobacterial Disease in South African Gold Miners A Case-Control Study [Internet]. Vol. 159, Am J Respir Crit Care Med. 1999. Available from: www.atsjournals.org

33. Andréjak C, Nielsen R, Thomsen V, Duhaut P, Sørensen HT, Thomsen RW. Chronic respiratory disease, inhaled corticosteroids and risk of non-tuberculous mycobacteriosis. Thorax. 2013;68(3).

34. Feng JY, Chen WC, Chen YY, Su WJ. Clinical relevance and diagnosis of nontuberculous mycobacterial pulmonary disease in populations at risk. Vol. 119, Journal of the Formosan Medical Association. 2020.

35. Corbett EL, Churchyard GJ, Hay M, Herselman P, Clayton T, Williams B, et al. The Impact of HIV Infection on Mycobacterium kansasii Disease in South African Gold Miners [Internet]. Vol. 160, Am J Respir Crit Care Med. 1999. Available from: www.atsjournals.org

36. Hoza AS, Mfinanga SGM, Rodloff AC, Moser I, König B. Increased isolation of nontuberculous mycobacteria among TB suspects in Northeastern, Tanzania: Public health and diagnostic implications for control programmes. BMC Res Notes. 2016 Feb 17;9(1).

37. Kone B, Sarro YS, Maiga M, Sanogo M, Somboro AM, Diarra B, et al. Clinical characteristics of non-tuberculous mycobacterial pulmonary infections in Bamako, Mali. Epidemiol Infect. 2018;146(3).

38. Pokam BT, Asuquo AE. Acid-Fast Bacilli Other than Mycobacteria in Tuberculosis Patients Receiving Directly Observed Therapy Short Course in Cross River State, Nigeria. Tuberc Res Treat. 2012;2012:1–4.

39. Jankovic M, Samarzija M, Sabol I, Jakopovic M, Katalinic Jankovic V, Zmak L, et al. Geographical distribution and clinical relevance of non-tuberculous mycobacteria in Croatia. International Journal of Tuberculosis and Lung Disease. 2013 Jun 1;17(6):836–41.

40. Sun Q, Yan J, Liao X, Wang C, Wang C, Jiang G, et al. Trends and Species Diversity of Non-tuberculous Mycobacteria Isolated From Respiratory Samples in Northern China, 2014–2021. Front Public Health. 2022;10.

41. Farnia P, Farnia P, Ghanavi J, Velayati AA. Epidemiological Distribution of Nontuberculous Mycobacteria Using Geographical Information System. In: Nontuberculous Mycobacteria (NTM): Microbiological, Clinical and Geographical Distribution. Elsevier; 2019. p. 191–321.

Aissata Boubakar CISSE
Mycobacteriology Unit
Laboratories and Biomedical Research Department
National Institute of Public Health
Bamako (Mali)
Phone: +223 75507091
Email: aissatacisse@yahoo.fr
June 12, 2024

Dear Academic Editor of Plos One journal,

I am pleased to submit herewith our point-by-point responses to remarks raised by the academic editor and reviewers of our manuscript titled "**Prevalence of non-tuberculous mycobacteria among people with acid-fast positive presumptive tuberculosis in Mali"**.

Points raised by academic editor

1. Please ensure that your manuscript meets PLOS ONE's style requirements, including those for file naming.

We have adapted the manuscript to the style of Plos One, with special attention to the titles and the presentation of the references.

2. Please provide additional details regarding participant consent. In the ethics statement in the Methods and online submission information, please ensure that you have specified what type you obtained (for instance, written or verbal, and if verbal, how it was documented and witnessed). If your study included minors, state whether you obtained consent from parents or guardians. If the need for consent was waived by the ethics committee, please include this information. Once you have amended this/these statement(s) in the Methods section of the manuscript, please add the same text to the "Ethics Statement" field of the submission form (via "Edit Submission").

We have reviewed the ethics section, specifying the type of consent required.

3. We note that the grant information you provided in the 'Funding Information' and 'Financial Disclosure' sections do not match

We have aligned the grant information in the two sections.

4. Authors must share the "minimal data set" for their submission

We submitted the minimal data used in the study in the MATLAB and SCV file named "Minimal data set".

5. Any changes to the reference list should be mentioned in the rebuttal letter that accompanies your revised manuscript
   - **7 references have been removed (1-5, 8 and 38 of initial manuscript);**
   - **34 references have been retained (11, 12, 14-40, 42, 44, 46, 47, 48 of revised manuscript)**
   - **16 new references have been added (1, 2, 3, 4, 5-10, 13, 41, 43, 45, 49, 50 of revised manuscript).**

<u>Points raised by Reviewers</u>

Reviewer #1:

1. Introduction part is not enough and Rewrite the introduction part with the current information. Major revision is required to get published. Rewrite the contents in the methodology and discussion part as mentioned in the reviewed manuscript

We have rewritten the introduction (adding more information about disease burden and laboratory diagnostics, and reorganised the structure), and added some additional information to the methodology and discussion parts. In doing so, we hope to have properly addressed this remark.

Reviewer #2:

1. Your article raises several questions about TB diagnosis and provides additional evidence for why smear microscopy as a first line diagnostic tool needed to be replaced with GeneXpert. However, it is interesting that GeneXpert also missed some cases that your inhouse method detected. Could you explain how your method achieved this?

We have provided an explanation for the higher sensitivity of the IS*6110* in-house qPCR method compared to Xpert in the discussion part. <u>Line 264-268 in discussion of</u>

manuscript: "**The observed high Ct values in our study indicates, however, a low quantity of MTB DNA in samples from these patients, that may have been positive upon repeated Xpert testing or by using the Xpert Ultra test, which is more sensitive by targeting both IS*6110* and another repetitive element IS*1081* for MTBc detection, in addition to the *rpoB* gene for assessing rifampicin susceptibility".**

2. Consider changing the title to 'Prevalence of non-tuberculous mycobacteria among acid-fast positive presumptive TB cases in Mali"

We have accepted to change the title of the manuscript to "Prevalence of non-tuberculous mycobacteria among people with acid-fast positive presumptive TB in Mali" (with a minor edit from "cases" to "people with").

3. Based on your results, your in-house qPCR for IS6110 was more sensitive than Xpert as it detected some additional MTBC cases from Xpert negative samples? How sure are you that these additional cases represented ongoing infection with MTBC and not previous exposure?

This study was part of the national drug-resistant TB survey, comprising both new and previously treated TB cases. The concern of false-positive MTBc detection in sputum from previously treated patients is mostly an issue for the highly sensitive, newer generation of rapid molecular assays, such as a result by "MTB detected - trace" by Xpert Ultra. However, even smear-microscopy could, in theory, identify non-viable AFB. The survey algorithm was not designed to include culture; hence we could not assess the viability of the detected MTBc and/or bacilli detected by microscopy, nor whether this reflected the presence of an NTM in combination with an ongoing MTBc infection, or rather an NTM following a previous MTBc infection. We have included this limitation in the discussion.

4. During the survey, at which point were people treated for TB, after ZN staining or based on Xpert results? During the survey was TB culture (gold standard for TB diagnosis) done for the ZN positive samples to confirm ongoing infection?

"All enrolled participants received anti-TB treatment. New patients were started on first-line TB treatment on the basis of an AFB microscopy. For patients with previously treated TB, in accordance with national guidelines, treatment was initiated based on the result of Xpert MTB/RIF testing".

5. If based on Xpert results, what happened to those who were Xpert-negative and later MTBC was detected by your inhouse method?

We have clarified this in the methodology part: "All enrolled participants received anti-TB treatment. New patients were started on first-line treatment based on the microscopy results. For previously treated patients, in accordance with national guidelines, re-treatment or RR-TB treatment was initiated based on Xpert's rifampicin-susceptibility result". Hence, no anti-TB treatment had to be started based on the in-house qPCR results because the results were received 1 year later.

6. The conclusion that there is the need for alternative diagnostics beyond Xpert is valid, as your study found that some Xpert-negative and AFB-positive samples could actually still be MTBC positive by other methods. However, it would be important to state that such methods would need to be validated with TB culture which is the gold standard.

We added this reflection in the discussion (line 287-289) and described also the place of culture for the diagnosis of NTM and monitoring of the treatment in the discussion part: "Nevertheless, even smear microscopy can still show non-viable AFB, and introduction of alternative/additional diagnostic assays or flows would need to be validated against culture".

7. For general comments:
    - Line 154-157: The sentence is too long and confusing, kindly rephrase using shorter sentences for easy comprehension.

We rewrote the sentence.

- Line 184: change from "male patients were more numerous" to "male patients were more".

We rephrased this sentence.

- Line 214-216: "The proportion of NTM versus Nocardia spp note the clinical relevance of these isolated bacteria were discussed in that study". What is the relevance of the statement? Can you rephrase for clarity?

We removed the ambiguous sentence.

- Line 252: Please check the arrangements of the references-some have been repeated and are outside the bracket.

We checked and corrected where needed.

- Check spacing before references in brackets- In some instances, there is no space between the last word and the reference number. Please be consistent with this throughout the manuscript.

We checked and corrected where needed.
