# Supplementary material for: Prevalence of non-tuberculous mycobacteria among people with acid-fast positive presumptive tuberculosis in Mali
Source: PLoS One. 2024 Dec 18;19(12):e0301731. doi: 10.1371/journal.pone.0301731 (PMC11654988; doi:10.1371/journal.pone.0301731)
Supplement: Attachment — Submitted filename: PONE-D-24-10019_R3.pdf [file pone.0301731.s007.pdf]

PLOS ONE

Prevalence of non-tuberculous mycobacteria among people with acid-fast positive presumptive tuberculosis in Mali

--Manuscript Draft--

**Manuscript Number:**	PONE-D-24-10019R3
**Article Type:**	Research Article
**Full Title:**	Prevalence of non-tuberculous mycobacteria among people with acid-fast positive presumptive tuberculosis in Mali
**Short Title:**	Prevalence of non-tuberculous mycobacteria among people with presumptive tuberculosis in Mali
**Corresponding Author:**	Aissata Boubakar CISSE, PharmD,Mcs, doctoral student<br>National Institute of Public Health: Institut National de Sante Publique<br>Bamako, MALI
**Keywords:**	Acid-fast bacilli; Mali; non-tuberculous mycobacteria; Drug resistance survey.
**Abstract:**	Background: Non-tuberculous mycobacteria (NTM) are environmental agents that can cause opportunistic pulmonary disease in humans and animals, often misdiagnosed as tuberculosis (TB). In this study, we describe the cases of NTM identified during the first national anti-TB drug resistance survey conducted in Mali and explore associated risk factors.<br><br>Methods: Sputum was collected from people presenting for pulmonary TB diagnosis from April to December 2019, regardless of age. Microscopy-positive patients were enrolled and tested using the Xpert MTB/RIF assay. A patient who tested negative for the Mycobacterium tuberculosis complex (MTBC) was tested for the presence of mycobacteria by amplification of the IS6110 and 16SrRNA (16S) genes through double quantitative real-time PCR, followed by nested PCR and Sanger sequencing of the IS6110-negative samples for NTM species identification.<br>Results: 1,418 sputum smear-positive patients were enrolled, including 1,199 new cases, 211 previously treated cases, and 8 whose previous treatment history was unknown. Based on the results of Xpert MTB/RIF assay and in-house PCR methods, 1,331 (93.9%) patients were positive for MTBC, 48 (3.4%) for NTM, and no species identification was possible for 39 (2.7%). Advanced age of 65 and over with an OR 8.8 (95% CI 2.3-33.2 and p=0.001) and previous TB treatment with an OR 3.4 (95% CI 1.2-9.6 and p=0.016) were the risk factors statistically associated with NTM detection. M. avium complex (MAC) was the predominant NTM species, detected in 20 cases.<br>Conclusion: Detection of NTM in people presumed to have TB is an ongoing challenge, confounding correct TB diagnosis. Concomitant use of microscopy and GeneXpert testing among at-risk individuals could improve patient management.
**Order of Authors:**	Aissata Boubakar CISSE, PharmD,Mcs, doctoral student<br>Anna S. Dean, PhD<br>Armand Van Deun, PhD<br>Jelle Keysers<br>Willem-Bram de Rijk, Bachelor<br>Mourad Gumusboga, Bachelor<br>Hawa SAMAKE, PhD<br>Seydou ARAMA, PharmD<br>Bassirou Diarra, MD, Msc, PhD<br>Fatoumata N'Dji Coulibaly, Bachelor<br>Ibrahim Djilla, Bachelor<br>Hawa Simpara, Msc

Powered by Editorial Manager® and ProduXion Manager® from Aries Systems Corporation

	Mamadou Berthé, MD, Msc, PhD
	Khadidia Ouattara, MD
	Yacouba Toloba, MD
	Ibrehima Guindo, PharmD, Msc, PhD
	Bouke Catherine de Jong, MD PhD
	Leen Rigouts, PhD
**Response to Reviewers:**	We have reviewed our list of references and have not made any changes. And we have accepted the minor corrections.
**Additional Information:**	
**Question**	**Response**
**Financial Disclosure**<br><br>Enter a financial disclosure statement that describes the sources of funding for the work included in this submission. Review the submission guidelines for detailed requirements. View published research articles from *PLOS ONE* for specific examples.<br><br>This statement is required for submission and **will appear in the published article** if the submission is accepted. Please make sure it is accurate.<br><br>**Funded studies**<br>Enter a statement with the following details:<br>• Initials of the authors who received each award<br>• Grant numbers awarded to each author<br>• The full name of each funder<br>• URL of each funder website<br>• Did the sponsors or funders play any role in the study design, data collection and analysis, decision to publish, or preparation of the manuscript?<br><br>Did you receive funding for this work?	Yes
Please add funding details.<br>as follow-up to "**Financial Disclosure**<br><br>Enter a financial disclosure statement that describes the sources of funding for the work included in this submission. Review the submission guidelines for detailed requirements. View published research	1st tuberculosis Drug resistance survey financed by the Global Fund

Powered by Editorial Manager® and ProduXion Manager® from Aries Systems Corporation

articles from *PLOS ONE* for specific examples.<br><br>This statement is required for submission and **will appear in the published article** if the submission is accepted. Please make sure it is accurate.<br><br>**Funded studies**<br>Enter a statement with the following details:<br>• Initials of the authors who received each award<br>• Grant numbers awarded to each author<br>• The full name of each funder<br>• URL of each funder website<br>• Did the sponsors or funders play any role in the study design, data collection and analysis, decision to publish, or preparation of the manuscript?<br><br>Did you receive funding for this work?"	
Please select the country of your main research funder (please select carefully as in some cases this is used in fee calculation).<br>as follow-up to "**Financial Disclosure**<br><br>Enter a financial disclosure statement that describes the sources of funding for the work included in this submission. Review the submission guidelines for detailed requirements. View published research articles from *PLOS ONE* for specific examples.<br><br>This statement is required for submission and **will appear in the published article** if the submission is accepted. Please make sure it is accurate.	MALI - ML

**Funded studies**<br>Enter a statement with the following details:<br>• Initials of the authors who received each award<br>• Grant numbers awarded to each author<br>• The full name of each funder<br>• URL of each funder website<br>• Did the sponsors or funders play any role in the study design, data collection and analysis, decision to publish, or preparation of the manuscript?<br><br>Did you receive funding for this work?"	
**Competing Interests**<br><br>Use the instructions below to enter a competing interest statement for this submission. On behalf of all authors, disclose any competing interests that could be perceived to bias this work—acknowledging all financial support and any other relevant financial or non-financial competing interests.<br><br>This statement is required for submission and **will appear in the published article** if the submission is accepted. Please make sure it is accurate and that any funding sources listed in your Funding Information later in the submission form are also declared in your Financial Disclosure statement.<br><br>View published research articles from *PLOS ONE* for specific examples.	The authors have declared that no competing interests exist.

Powered by Editorial Manager® and ProduXion Manager® from Aries Systems Corporation

**NO authors have competing interests**<br><br>Enter: *The authors have declared that no competing interests exist.*<br><br>**Authors with competing interests**<br><br>Enter competing interest details beginning with this statement:<br><br>*I have read the journal's policy and the authors of this manuscript have the following competing interests: [insert competing interests here]*<br><br>* typeset	
**Ethics Statement**<br><br>Enter an ethics statement for this submission. This statement is required if the study involved:<br><br>• Human participants<br>• Human specimens or tissue<br>• Vertebrate animals or cephalopods<br>• Vertebrate embryos or tissues<br>• Field research<br><br>Write "N/A" if the submission does not require an ethics statement.<br><br>General guidance is provided below. Consult the submission guidelines for detailed instructions. **Make sure that all information entered here is included in the Methods section of the manuscript.**	The drug resistance survey protocol in which a secondary analysis of samples is described in the informed consent was accepted by the ethics committee of the National Institute of Public Health Research by decision No. 08/2018/CE-INRSP on March 21, 2018. The objectives and expected results of the study were clearly explained in the language understood by the patient. Patients were asked for permission to use their samples for additional analyses, including outside Mali. Oral and written consent were obtained from patients over 18 years of age. For patients under the age of 18, after obtaining the prior oral and written consent of the parent or companion, the child's assent to participate in the study was obtained in the presence of a witness other than the parent or companion.

Powered by Editorial Manager® and ProduXion Manager® from Aries Systems Corporation

**Format for specific study types**<br><br>**Human Subject Research (involving human participants and/or tissue)**<br>• Give the name of the institutional review board or ethics committee that approved the study<br>• Include the approval number and/or a statement indicating approval of this research<br>• Indicate the form of consent obtained (written/oral) or the reason that consent was not obtained (e.g. the data were analyzed anonymously)<br><br>**Animal Research (involving vertebrate animals, embryos or tissues)**<br>• Provide the name of the Institutional Animal Care and Use Committee (IACUC) or other relevant ethics board that reviewed the study protocol, and indicate whether they approved this research or granted a formal waiver of ethical approval<br>• Include an approval number if one was obtained<br>• If the study involved *non-human primates*, add *additional details* about animal welfare and steps taken to ameliorate suffering<br>• If anesthesia, euthanasia, or any kind of animal sacrifice is part of the study, include briefly which substances and/or methods were applied<br><br>**Field Research**<br><br>Include the following details if this study involves the collection of plant, animal, or other materials from a natural setting:<br>• Field permit number<br>• Name of the institution or relevant body that granted permission	
**Data Availability**<br><br>Authors are required to make all data underlying the findings described fully available, without restriction, and from the time of publication. PLOS allows rare exceptions to address legal and ethical concerns. See the PLOS Data Policy and FAQ for detailed information.	Yes - all data are fully available without restriction

A Data Availability Statement describing where the data can be found is required at submission. Your answers to this question constitute the Data Availability Statement and **will be published in the article**, if accepted.<br><br>**Important:** Stating 'data available on request from the author' is not sufficient. If your data are only available upon request, select 'No' for the first question and explain your exceptional situation in the text box.<br><br>Do the authors confirm that all data underlying the findings described in their manuscript are fully available without restriction?	
**Describe where the data may be found in full sentences. If you are copying our sample text, replace any instances of XXX with the appropriate details.**<br><br>• If the data are **held or will be held in a public repository**, include URLs, accession numbers or DOIs. If this information will only be available after acceptance, indicate this by ticking the box below. For example: *All XXX files are available from the XXX database (accession number(s) XXX, XXX.*).<br>• If the data are all contained **within the manuscript and/or Supporting Information files**, enter the following: *All relevant data are within the manuscript and its Supporting Information files.*<br>• If neither of these applies but you are able to provide **details of access elsewhere**, with or without limitations, please do so. For example:<br><br>*Data cannot be shared publicly because of [XXX]. Data are available from the XXX Institutional Data Access / Ethics Committee (contact via XXX) for researchers who meet the criteria for access to confidential data.*<br><br>*The data underlying the results presented in the study are available from (include the name of the third party*	All relevant data are within the manuscript and its Supporting Information files.

and contact information or URL).<br>• This text is appropriate if the data are owned by a third party and authors do not have permission to share the data.<br><br>* typeset	
Additional data availability information:	Tick here if your circumstances are not covered by the questions above and you need the journal’s help to make your data available.

Powered by Editorial Manager® and ProduXion Manager® from Aries Systems Corporation

Manuscript Click here to access/download;Manuscript;Manuscrit.rtf

Prevalence of non-tuberculous mycobacteria among people with acid-fast positive presumptive tuberculosis in Mali

Prevalence of non-tuberculous mycobacteria among people with presumptive tuberculosis in Mali

Aissata Boubakar CISSE[1]*, Anna S. DEAN[2], Armand Van DEUN[3†], Jelle KEYSERS[3], Willem-Bram De RIJK[3], Mourad GUMUSBOGA[3], Hawa SAMAKE[1], Seydou ARAMA[1], Bassirou DIARRA[5], Ibrahim DJILLA[1], Fatoumata N. COULIBALY[1], Hawa SIMPARA[1], Mamadou BERTHE[1], Khadidia OUATTARA[6], Yacouba TOLOBA[6], Ibrehima GUINDO[1], Bouke de JONG[3], Leen RIGOUTS[3,4].

1- National Institute of Public Health, Bamako, Mali
2- Word Health Organization, Geneva
3- Mycobacteriology Unit, Institute of Tropical Medicine, Antwerp, Belgium
4- Belgian Coordinated Collections of Microorganisms, Antwerp, Belgium
5- University Clinical Research Center, Bamako, Mali
6- University Teaching hospital of Point G, Bamako, Mali

† Deceased in September 2023

* Corresponding author

E-mail: aissatacisse@yahoo.fr (ABC)

Abstract

Background: Non-tuberculous mycobacteria (NTM) are environmental agents that can cause opportunistic pulmonary disease in humans and animals, often misdiagnosed as tuberculosis (TB). In this study, we describe the cases of NTM identified during the first national anti-TB drug resistance survey conducted in Mali and explore associated risk factors.

Methods: Sputum was collected from people presenting for pulmonary TB diagnosis from April to December 2019, regardless of age. Microscopy-positive patients were enrolled and tested using the Xpert MTB/RIF assay. A patient who tested negative for the *Mycobacterium tuberculosis* complex (MTBC) was tested for the presence of mycobacteria by amplification of the IS*6110* and 16SrRNA (16S) genes through double quantitative real-time PCR, followed by nested PCR and Sanger sequencing of the IS*6110*-negative samples for NTM species identification.

Results: 1,418 sputum smear-positive patients were enrolled, including 1,199 new cases, 211 previously treated cases, and 8 whose previous treatment history was unknown. Based on the results of Xpert MTB/RIF assay and in-house PCR methods, 1,331 (93.9%) patients were positive for MTBC, 48 (3.4%) for NTM, and no species identification was possible for 39 (2.7%). Advanced age of 65 and over with an OR 8.8 (95% CI 2.3-33.2 and p=0.001) and previous TB treatment with an OR 3.4 (95% CI 1.2-9.6 and p=0.016) were the risk factors statistically associated with NTM detection. *M. avium complex* (MAC) was the predominant NTM species, detected in 20 cases.

Conclusion: Detection of NTM in people presumed to have TB is an ongoing challenge, confounding correct TB diagnosis. Concomitant use of microscopy and GeneXpert testing among at-risk individuals could improve patient management.

1. Introduction

The *Mycobacterium* genus groups species of the *Mycobacterium tuberculosis* complex (MTBC) responsible for tuberculosis (TB) in humans and animals, *M. leprae*, the causative agent of leprosy, and the species of mycobacteria commonly known as atypical or non-tuberculous mycobacteria (NTM), including *M. ulcerans* responsible for Buruli ulcer. Unlike members of the MTBC group, NTM are not obligatory parasites of humans, yet normal residents of soil and water, and can be found in natural and treated water sources [1]. More than 200 species of NTM have been officially recognised [2], of which around 25 are known to be strongly associated with disease in humans. Some species have been associated with pulmonary diseases causing symptoms similar to TB [1]. Because of their habitat, humans are exposed to these bacteria daily. Therefore, NTM disease must be distinguished from simple colonisation or contamination of clinical samples, for example from tap water [1,3]. Unlike TB, the global epidemiology of disease caused by NTM is not well known. NTM isolation from clinical specimens is mainly reported in industrialised countries, with variable prevalence and incidence. Studies based on pulmonary specimen isolates reported a prevalence of 1.4 to 6.6 per 100,000 persons in the USA from 2004 to 2006 [4], 9.8 in 2010 in Ontario, Canada [5], and 5.8 per 100,000 in Germany [6] in 2020. Incidence of 6.1 per 100,000 in 2012 in England [7] and 5.3 per 100, 000 in Germany in 2020 [6] have also reported. In TB-endemic countries, NTM is being reported less frequently and mainly in populations at risk, particularly people with predisposing conditions or compromised

immunity [8]. However, experiences in industrialised countries have shown that a declining TB burden has also increased ~~in~~ the number of NTM cases detected. With the strengthening of TB programmes in another setting, we may also anticipate a similar scenario, posing a growing diagnosis and clinical challenge in low-and middle-income countries [9]. The diagnosis of NTM lung disease is based on clinical, radiological, and microbiological criteria [1]. Laboratory-based detection of NTM, with differentiation from MTBC and identification to the species level, is mainly inaccessible in most resource-limited countries. Microscopy, the most accessible technique, identifies MTBC and NTM as acid-fast bacilli (AFB) but fails to distinguish them. Since 2010, the World Health Organization (WHO) has recommended rapid molecular tests such as GeneXpert MTB/RIF (Xpert) as the initial test for TB diagnosis, exhibiting a higher sensitivity and specificity [10]. This assay only identifies the presence of MTBC species in the sample. A negative result for MTBC by Xpert obtained on AFB-positive sputum could indicate NTM [11]. In Mali, cases of NTM infection have been described, particularly in patients under investigation for failure of anti-TB treatment or who have relapsed after TB cure [12]. Following the introduction of Xpert in the country, plausible cases of NTM infection with an AFB-positive smear and a negative Xpert have been reported more frequently [13].

In this study, we describe the proportion of MTBC and NTM detected in patients presenting for initial or previously treated TB diagnoses in Mali in the context of a nationwide drug resistance survey [14]. We also explored risk factors associated with NTM detection.

2. Methods

2.1. Design of the study

From April to December 2019, consecutive eligible patients were recruited from 78 of 86 TB diagnostic laboratories, representing 91% of the country's TB laboratory network. Eight laboratories notified less than 10 new bacteriologically confirmed pulmonary TB cases in 2017 and were not included for logistic reasons. Eligible participants were defined as people undergoing evaluation for pulmonary TB, regardless of age, with and without a prior treatment history. The sample size of 994 new cases was calculated to meet the primary objective of the survey for measuring the percentage of new pulmonary TB cases resistant to rifampicin, for which a recruitment period of 4 months was estimated to be required [15].

2.2. Sample collection and patient enrolment

Two sputum samples of approximately 5 mL were collected from each patient in the participating laboratories. Smears were made directly from freshly collected sputum, stained according to the acid-fast staining technique used at the center (auramine for most sites and Ziehl-Neelsen for several small centers), and in line with international guidelines for diagnosing TB by microscopy [16]. If one of these two sputum samples was AFB positive, the patient was included in the drug resistance survey after giving informed consent, and an additional sample was collected.

The nurse completed the study questionnaire with the recruited patient. The questionnaire covered socio-demographic data, information on previous TB treatment, and pulmonary disease history (duration and chronicity of respiratory signs). Relevant clinical and treatment-related

information was also provided to the patient. All enrolled participants received anti-TB treatment. New patients were started on first-line TB treatment based on AFB microscopy. According to national guidelines, treatments were initiated based on the Xpert MTB/RIF testing results of patients with previously treated TB.

2.3. Initial GeneXpert testing and specimen storage

The first two sputum samples collected were preserved in 5 mL of OMNIgene® SPUTUM (OM-S) decontamination reagent (DNA Genotek Inc, Canada) according to the manufacturer's recommendations [17]. In contrast, the third sample was preserved in 10 mL of 96% ethanol. The conditioned samples were transported by public transport at ambient temperature in triple packaging to the National TB Reference Laboratory (NTRL) in Bamako.

The Xpert MTB/RIF assay (Cepheid, CA, USA) was performed at the NTRL using 2 mL of one OM-S-preserved sample, as per the manufacturer's recommendations (Cepheid and Genotek).

Leftovers of the OM-S-preserved samples were then stored in cryotubes as sediment obtained after centrifugation at 4000 g for 15 minutes and mixed with 96% alcohol at an equal volume (0.5 mL sediment and 0.5 mL ethanol).

2.4. Molecular detection and identification of NTM species

For molecular detection and identification of NTM species, according to the study protocol, samples from the following patients were transported at ambient temperature to the WHO Supranational TB Reference Laboratory at the Institute of Tropical Medicine (ITM) in Antwerp, Belgium:

- cases with rifampicin resistance by Xpert MTB/RIF, regardless of anti-TB treatment history;
- all previously treated cases, regardless of Xpert MTB/RIF test result;
- ~~and cases for whom MTBC was not detected by Xpert MTB/RIF~~.

In this article, we describe only the molecular methods employed and the results obtained for species identification of probable NTM.

Upon arrival at ITM, ethanol-preserved samples were centrifuged at 4000 g for 15 minutes, digested by overnight proteinase K incubation, followed by lysis using an internal lysis buffer, after which DNA was extracted using the Modified Maxwell-LEV® automated extraction method (Promega, USA) [18]. A duplex quantitative real-time polymerase chain reaction (qPCR) was performed, targeting the IS*6110* gene to detect the MTBC and the 16S gene to detect all species of the *Mycobacterium* genus. The primers and probes used are presented in Table 1, while the interpretation of results is depicted in Table 2.

Table 1. Primers and probes for qPCR to simultaneously detect *Mycobacterium tuberculosis* complex and all mycobacterial species.

Target	Name	Sequences
**IS*6110* gene**	IS*6110*_S<br>IS*6110*_AS	5'-CCTGAAAGACGTTATCCACCATAC-3'<br>5'-GGC-TAG-TGC-ATT-GTC-ATA-GGA-G-3'
	IS6110_P_DFO	DFO-TCT-CAG-TA(lnC)-AC(lnA)-TC(lnG)-ATC-CGG-T-BHQ2
**16S gene**	Myco_Genus_S_v3<br>Myco_Genus_AS_v2	5'-ATGCAAGTCGAACGGAAAGG-3'<br>5'-TCTGCCCGTATCGCC-C-3'
	Myco_Genus_P_v2	FAM-CAA-AC(lnC)-AC(lnC)-TA(lnC)-GA(lnG)-CTC-EDQ

Table 2. Interpretation of duplex IS*6110*-16S qPCR results

**16S qPCR Cycle threshold (Ct)**	**IS*6110* qPCR result**	
	**Positive**	**Negative**
**Positive with any Ct value**	-	No MTBC
**Positive and ≥IS*6110* Ct**	MTBC	
**Positive and <IS*6110* Ct**	MTBC + NTM	
**Negative**	MTBC (low bacillary load)	No mycobacteria species identification possible

MTBC = *Mycobacterium tuberculosis* complex; NTM = non-tuberculous mycobacteria: Ct = Cycle threshold

Subsequently, a nested conventional PCR targeting the 16S gene was performed on samples identified as NTM by the duplex qPCR. The primers and probes used were as follows:

- P1 (first PCR): 5'-TGCTTAACACATGCAAGTCG-3'
- P2 new (first PCR): 5'-TCTCTAGACGCGTCCTGTGC-3'
- P7 (nested PCR): 5'-CATGCAAGTCGAACGGAAAGG-3'
- P16 new (nested PCR): 5'-AAGCCGTGAGATTTCACGAACA3'

Amplicons that were positive on the agarose gel underwent Sanger sequencing. A negative PCR result was interpreted as the absence of MTBC or NTM.

Sequencing was subcontracted to BaseClear (BE Leiden, The Netherlands). After entering the sequence into the Basic Local Alignment Search Tool (BLAST) of the National Centre for Biotechnology Information (NCBI), we compared it to reference strains sequences on NCBI such as ATCC 25791, ATCC BAA-614, ATCC 27282, ATCC 51457, 4773, ATCC 700504, ATCC 25276, ATCC 35752, ATCC 13950. We interpreted the results obtained as follows:

- If a sequence match was found on the NCBI, the NTM species was identified.

    - In case of 100% similarity (no mismatches), the species name was assigned.
    - In case 1-3 bp mismatches with the closest species, the “species-like” name was assigned.
- If no match or >3 bp difference with the closest species was found on NCBI, the result was interpreted as *Mycobacterium* species.
- If there were overlapping peaks in the sequence, indicating the presence of several NTM, the result was interpreted as "Mixture of NTM ".

2.5. Data analysis

The data collected using the survey and sample submission forms were entered into EpiInfo version 7.2.5.0. The variables analysed were age, sex, region of residence, history of illness, and history of anti-TB treatment. Percentages, medians, and means were calculated by crosstab, and p-values and odds ratios with 95% confidence intervals were generated using regression analysis in IBM SPSS Statistics version 21X64 (IBM Corp., NY, USA).

2.6. Ethical considerations

The drug resistance survey protocol in which a secondary analysis of samples is described in the informed consent was accepted by the National Institute of Public Health Research ethics committee by decision No. 08/2018/CE-INRSP on March 21, 2018.~~The objectives and expected results of the study were clearly explained in the language understood by the patient.~~ Patients were asked permission to use their samples for additional analyses, including outside Mali. ~~Oral and written consent were obtained from patients over 18 years of age. For patients under the age of 18, after obtaining the prior oral and written consent of the parent or companion, the child's~~

~~assent to participate in the study was obtained in the presence of a witness other than the parent or companion.~~

3. Results

3.1. Mycobacteria detected

A total of 1,418 AFB-positive people with presumed pulmonary TB were enrolled in the drug resistance survey, including 1,199 new cases, 211 previously treated cases, and 8 whose previous treatment history was unknown (Figure 1). Based on Xpert MTB/RIF results, 1,296 (91.4%) patients were positive for MTBC, while 1MTBC was not detected for 22 (8.6%). The duplex IS6110-16S qPCR performed for 119 of these 122 patients identified another 35 MTBC, bringing the total number of patients with MTBC to 1,331 (93.9%). Of these 35 additional cases, MTBC alone was detected for six, while 29 carried probable mixtures of MTBC and NTM.

Fig 1. **Sample and analysis flow with summary of results,** MTBC=*Mycobacterium tuberculosis* complex; n= number; NTM = non-tuberculous mycobacteria.

For 81 (68,1%) of 119 patients tested, only the 16S target reacted positively (presumed NTM), while the PCR remained negative for both targets in 3 (2,5%) patients. The nested conventional 16S PCR performed for the 81 presumed NTM detected by IS*6110*-16S qPCR was positive for 48 or 3.4% (48/1418) of all enrolled patients, of which 33 could be identified to the species level by sequencing. The remaining 33 of 81 presumed NTM had a negative nested PCR result, thus no sequencing could be performed (Fig 1).

Thus, eleven species or groups of species could be identified, with a predominance of the *M. avium* complex (MAC) representing 20 cases or 42% of all species detected, *M. intracellulare* complex in 13 patients (27%), *M. avium* in five (10%) and *M. colombiense* in two (4 %) (Fig 2).

Fig 2. **Sequencing results for the identification of NTM species**

3.2. Detection of NTM in relation to smear-microscopy results

The participating laboratories were notified of the quantification of AFB for 1414 of 1418 patients. A significantly higher proportion of NTM was detected in patients with lower AFB-grades: 7% for 1+ and scanty (Table 3), compared to 2% among 3+ patients (respectively OR 4.065, 95% CI: 1.876-8.808, p=0.000 and OR 4.3, 95% CI: 2.012-9.189, p=0.000). The difference was not significant compared to grade 2+ samples (p=0.462).

Table 3. Proportion of NTM and MTB detection in relation to quantitative sputum-smear microscopy results

Final sputum smear result	MTBC n (%)	NTM n (%)	No species identification possible n (%)	Total n (%)
**Scanty**	167 (83)	13 (7)	21 (10)	201
**1+**	170 (89)	14 (7)	8 (4)	192
**2+**	259 (94)	7 (3)	8 (3)	274
**3+**	731 (98)	14 (2)	2 (0)	747
**Not quantified**	0	0	4 (100)	4
**Total**	**1327 (93.6)**	**48 (3.4)**	**43 (3)**	**1418 (100)**

MTBC = *M. tuberculosis* complex; NTM = non-tuberculous mycobacteria; n =number

3.3. Socio-demographic, clinical characteristics and risk factors for NTM infection

Male patients were more common among study participants and among those with identified NTM, with a sex ratio of respectively 2.1 and 1.5 when compared to women, yet NTM presence was not associated with gender (Table 4). On the contrary, older age was significantly associated with NTM detection. The median age was 37 years for all patients and 59.5 years for those with NTM detected. Compared to the under-34 age group, the 35-64 age group had greater odds (OR 3.4, 95% CI: 1.1-10.3, p=0.03) of NTM detection, which further increased for the over-65 age group (OR 8.8, 95% CI: 2.3-33.2 p=0.001 (Table 4). Also, previously treated patients were more likely than new cases to have NTM in their sputum (OR 3.4, 95% CI: 1.2-9.6, p=0.016) (Table 4). We found no significant associations between NTM detection and HIV status or chronic respiratory signs. Regarding the patient's residence, patients from the Mopti region were more likely to have NTM detected than Bamako residents (OR 4.1, 95% CI: 1.0-16.4, p=0.04). (Table 4).

Table 4. Multivariate logistic regression analyses of demographic and clinical variables as risk factors of NTM infection

Variables		Total patients n (%)	NTM detected n (%)	OR	p-value
**Gender n=1379**	Female	434 (31.5)	19 (39.6%)	0.8 [0.3-2.04]	0.7
	Male	945 (68.5)	29 (60.4%)		
**Age n=1379**	Median	37	**59,5**		
**Age group**	0-34 years (Reference)	603 (43.7)	4 (8.3)		
	35-64 years	659 (47.8)	29 (60.4)	**3.4 [1.1-10.3]**	**0.03**
	≥65 years	117 (8.5)	15 (31.3)	**8.8 [2.3-33.2]**	**0.001**
**History of treatment n=1371**	New	1165 (84.5)	30 (62.5)		
	Previously treatment	206 (14.9)	18 (37.5)	**3.4 [1.2-9.6]**	**0.016**
**HIV status n=1092**	Positive	77 (5.6)	4 (8.3)	2.8 [0.8-8.9]	0.07
	Negative	1015 (73.6)	29 (60.4)		
**Chronic clinical signs n=1174**	Yes	442 (37.6)	24 (60)	0.7 [0.3-1.8]	0.5
	No	752 (62.4)	16 (40)		
**Region of residence at the time of diagnosis n=1348**	Bamako (Reference)	451 (33.5)	5 (10.9)		
	Kayes	101 (7.5)	4 (8.7)	2.7 [0.5-12.7]	0.2
	Koulikoro	217 (16.1)	7 (15.2)	1.5 [0.3-7.4]	0.5
	Sikasso	155 (11.5)	12 (26.1)	2.9 [0.7-10.8]	0.10
	Ségou	147 (10.9)	7 (15.2)	3.2 [0.8-12.7]	0.09
	Mopti	191 (14.2)	7 (15.2)	**4.1 [1.02-16.4]**	**0.04**
	Northern regions	86 (6.4)	4 (8.7)	4.2 [0.7-25.2]	0.1

NTM = non-tuberculous mycobacteria; n = number; OR = odds ratio

4. Discussion

The proportion of sputum-AFB-positive patients and MTBC detected by Xpert MTB/RIF among people presumed to have TB in our study was slightly lower than the rate reported in other drug resistance surveys, ranging from 95.8%-97.7% [19–22]. The confirmed proportion with NTM in our study was higher than in TB drug-resistance surveys in Pakistan (0.7%, 13/1972) [19] and in India (1.2%, 35/2,938) [23], yet lower than the 6.4% (317/4917) observed in China [24]. This result may reflect regional differences in the occurrence of these mycobacteria and the diseases they cause worldwide [25,26].

NTM detection was more likely among paucibacillary patients as determined by smear microscopy, corroborating findings from Nigeria where the AFB-smear grading was relatively lower for patients with NTM detected than for those with MTBC [27], and higher AFB grading was predictive for MTBC detection [28]. With age, the risk of developing NTM infection increases. Still, we did not find a significant association between gender and NTM infection; contrary to other studies, females have been more frequently associated with NTM infections, especially at older ages [11,29,30]. Previous anti-TB treatment was another risk factor for NTM infection in our study, confirming findings from NTM pulmonary disease in China and South Africa [31,32]. Nevertheless, we did not find an association between NTM detection and chronic respiratory signs in our study, in contrast with the described association between NTM infection and lung disease or chronic respiratory symptoms in studies from China and Denmark [33,34].

HIV infection has frequently been identified as a risk factor for NTM infections and disease, especially in situations of advanced immunosuppression [27,35,36]. In our study, the trend toward an association between HIV positivity and NTM detection was not significant, corroborating previous observations in Mali [37]. No information about anti-retroviral treatment

was gathered as part of the drug resistance survey. Still, such treatment is to be started upon HIV diagnosis as per national guidelines in Mali. While patients from the Mopti region had higher odds of having NTM detected in their sputum than those from the Bamako region, further investigation of the environmental conditions in these regions would be needed, especially as NTM are environmental bacteria and have often been isolated at different frequencies in different geographical areas [25,30,38]. MAC species were the most commonly NTM detected in our survey. The predominance of MAC has been reported in pulmonary NTM infections in many countries around the world [25,26,39,40]. In Mali, between 2004 and 2013, *M. avium* was the most frequently identified species of the MAC complex [12,37].

Employing an additional in-house qPCR for IS*6110* in our study has increased the detection of MTBC. The MTBC-specific repetitive element IS*6110* is a suitable target for MTBC detection assays [41]. The applied qPCR IS*6110* has shown good sensitivity and specificity for detecting MTBC compared to culture [42] and higher performance when compared to the Xpert MTB/RIF assay [43]. The observed high Ct values in our study indicate, however, a low quantity of MTB DNA in samples from these patients, which may have been positive upon repeated Xpert testing [44] or by using the Xpert Ultra test, which is more sensitive by targeting both IS*6110* and another repetitive element IS*1081* for MTBC detection, in addition to the *rpoB* gene for assessing rifampicin susceptibility [45]. The higher positivity rate by qPCR compared to the conventional nested PCR we observed in our study could be partially attributable to the non-specificity of the 16S primers employed for the in-house qPCR, which also detects species belonging to closely related genera like *Rhodococcus* and *Nocardia*, hence potentially overestimating the NTM rate. These closely related species can indeed be detected in smear-positive samples not containing tubercle bacilli, ~~such~~ as documented in the ~~USA~~ with 78.4%

(134/171) of AFB-positive specimens yielding a positive culture with NTM or *Nocardia* spp, while 14.6% (25/171) of AFB-positive specimens grew MTBC and another 7% (12/171) remained negative in culture [46]. In another study in Nigeria, out of 32 smear-positive samples identified as not containing TB bacilli, six were NTM (18.75%), 14 were corynebacteria (43.75%), and 12 were rhodococci (37.5%) [28]. Our study did not further identify the non-MTBC/non-NTM samples. In addition, the lower sensitivity of the conventional nested PCR may have hampered confirmation by sequencing in case of negative results or low amplicon yield. Indeed, the in-house qPCR has already shown good sensitivity, while limited loss of DNA has been described during multiple handling steps for conventional nested PCR [47,48]. This lower sensitivity is reflected in the sample's AFB grading, with fewer non-identified samples among the strong positive specimens.

This study was part of the national TB drug resistance survey, comprising new and previously treated cases. The concern of false-positive MTBC detection in sputum from previously treated patients has been chiefly raised for the highly sensitive, newer generation of rapid molecular assays, such as the "MTB detected Trace" result by Xpert Ultra [49,50]. Nevertheless, even smear microscopy can still show non-viable AFB, and the introduction of alternative/additional diagnostic assays or flows would need to be validated against culture.

Our survey was not designed to include culture in the diagnostic algorithm, and hence, we could not assess the viability of the detected MTBC and NTM bacilli, nor whether co-detection of MTBC and NTM reflected simultaneous infection, or rather an NTM infection following MTBC infection in previously treated patients, which was still detectable due to remnant DNA.

Also, we did not investigate whether the detected NTM was attributable to actual infection, colonization, or sample contamination. We could not assess the American Thoracic Society

criteria to detect NTM in at least 3 different samples per patient, given the ~~design of the survey~~ and the retrospective nature of molecular analysis to detect NTM. However, study participants were people with respiratory signs undergoing evaluation for pulmonary TB attending health centers or patients failing TB treatment. Finally, we do not describe the outcome of patients in whom NTM was detected. Still, all were started on 1st line TB therapy or multidrug-resistant treatment following the results of initial microscopy and Xpert testing.

5. Conclusion

Among sputum-AFB-positive patients presenting in Mali, 3.4% were due to NTM. Particular attention must be paid to populations at risk, such as elderly patients and patients with a history of previous treatment. TB prevention and care programmes in resource-limited countries should ensure that their routine diagnostic algorithms can detect such cases, based on the concurrent use of rapid molecular TB diagnostics and microscopy without other molecular assays to detect NTM. However, culture is still necessary to isolate these mycobacteria and to monitor treatment in the event of confirmation of NTM disease. Clinical assessment and management of NTM infections remain a challenge worldwide.

Acknowledgements

We would like to thank the TB management sites for recruiting patients, collecting and transporting samples, the country's regional health departments for regional coordination of the drug resistance study, and Olga Tosas AUGUET for her assistance in planning the survey, monitoring the field activities and preliminary data analysis~~”~~.

References

1. Griffith DE, Aksamit T, Brown-Elliott BA, Catanzaro A, Daley C, Gordin F, et al. An official ATS/IDSA statement: Diagnosis, treatment, and prevention of nontuberculous mycobacterial diseases. Am J Respir Crit Care Med. 2007;175: 367–416. doi:10.1164/rccm.200604-571ST
2. Pascapurnama DN, Zavitri NG, Koesoemadinata RC, Cahyadi AI, Chaidir L. Identification of Significant Pathogenic Nontuberculous Mycobacteria Species from Presumptive TB Patients Using Partial hsp65 Gene Sequencing. Infect Drug Resist. 2023;16. doi:10.2147/IDR.S419956
3. Haworth CS, Banks J, Capstick T, Fisher AJ, Gorsuch T, Laurenson IF, et al. British Thoracic Society guidelines for the management of non-tuberculous mycobacterial pulmonary disease (NTM-PD). Thorax. 2017;72: iii1-ii64. doi:10.1136/thoraxjnl-2017-210927
4. Prevots DR, Shaw PA, Strickland D, Jackson LA, Raebel MA, Blosky MA, et al. Nontuberculous mycobacterial lung disease prevalence at four integrated health care delivery systems. Am J Respir Crit Care Med. 2010;182: 970–976. doi:10.1164/rccm.201002-0310OC
5. Marras TK, Mendelson D, Marchand-Austin A, May K, Jamieson FB. Pulmonary nontuberculous mycobacterial disease, Ontario, Canada, 1998-2010. Emerg Infect Dis. 2013;19: 1889–1891. doi:10.3201/eid1911.130737
6. Corbett C, Finger P, Heiß-Neumann M, Bohnert J, Eder IB, Eisele M, et al. Development of prevalence and incidence of non-tuberculous mycobacteria in German laboratories from 2016 to 2020. Emerg Microbes Infect. 2023;12. doi:10.1080/22221751.2023.2276342
7. Shah NM, Davidson JA, Anderson LF, Lalor MK, Kim J, Thomas HL, et al. Pulmonary Mycobacterium avium-intracellulare is the main driver of the rise in non-tuberculous mycobacteria incidence in England, Wales and Northern Ireland, 2007-2012. BMC Infect Dis. 2016;16. doi:10.1186/s12879-016-1521-3
8. Varma-Basil M, Bose M. Mapping the Footprints of Nontuberculous Mycobacteria. Nontuberculous Mycobacteria (NTM): Microbiological, Clinical and Geographical Distribution. Elsevier; 2019. pp. 155–175. doi:10.1016/B978-0-12-814692-7.00008-5
9. Iseman MD, Marras TK. The importance of nontuberculous mycobacterial lung disease. Am J Respir Crit Care Med. 2008;178: 999–1000. doi:10.1164/rccm.200808-1258ED
10. WHO. Module 3: Diagnosis WHO operational handbook on tuberculosis Rapid diagnostics for tuberculosis detection. 2021.
11. Kong L, Xie B, Liu Q, Hua L, Bhusal A, Bao C, et al. Application of acid-fast staining combined with GeneXpert MTB/RIF in the diagnosis of non-tuberculous mycobacteria pulmonary disease. International Journal of Infectious Diseases. 2021;104: 711–717. doi:10.1016/j.ijid.2020.12.091
12. Maiga M, Siddiqui S, Diallo S, Diarra B, Traoré B, Shea YR, et al. Failure to recognize nontuberculous mycobacteria leads to misdiagnosis of chronic pulmonary tuberculosis. PLoS One. 2012;7: e36902. doi:10.1371/journal.pone.0036902

13. Toloba Y, Cissé AB, Soumaré D, Ouattara K, Kanouté T, Koné D, et al. Apport du GeneXpert dans le diagnostic de la tuberculose et de la résistance à la rifampicine au Mali après 22 mois d'acquisition. Rev Mal Respir. 2017;34: A216–A217. doi:10.1016/j.rmr.2016.10.515

14. World Health Organization. Global tuberculosis report 2020. World Health Organization; 2020.

15. World Health Organization. Guidelines for surveillance of drug resistance in tuberculosis 5 th Edition. 2015.

16. Lumb R, Van Deun A, Bastian I, Fitz-Gerald M, Pathology SA, Stop TB Partnership (World Health Organization). Laboratory diagnosis of tuberculosis by sputum microscopy : the handbook. 2013.

17. DNA Genotek Inc. How can I use OMNIgene®•SPUTUM? Use Case 4: Reflex testing: Microscopy centre with or without GeneXpert® centrifuge. 2016 [cited 4 Apr 2017]. Available: www.dnagenotek.com•

18. Corporation P. Maxwell(R) 16 Cell LEV DNA Purification Kit Technical Bulletin TB383. 2012 [cited 11 Mar 2024]. Available: www.promega.com

19. Tahseen S, Qadeer E, Khanzada FM, Rizvi AH, Dean A, Van Deun A, et al. Use of Xpert® MTB/RIF assay in the first national antituberculosis drug resistance survey in Pakistan. International Journal of Tuberculosis and Lung Disease. 2016;20: 448–455. doi:10.5588/ijtld.15.0645

20. Lopes C, Kundu D, Da Costa Barreto I, da Cruz B, Siva Kumar S, Ramalingam S, et al. Experience on the first national anti-TB drug resistance survey (DRS) in Timor-Leste. Glob Health Res Policy. 2022;7: 2–9. doi:10.1186/s41256-022-00249-z

21. Aia P, Kal M, Lavu E, John LN, Johnson K, Coulter C, et al. The burden of drug-resistant tuberculosis in Papua New Guinea: Results of a large population-based survey. PLoS One. 2016;11: 2–9. doi:10.1371/journal.pone.0149806

22. Iem V, Dean A, Zignol M, Vongvichit P, Inthavong D, Siphanthong S, et al. Low prevalence of MDR-TB in Lao PDR: results from the first national anti-tuberculosis drug resistance survey. Tropical Medicine and International Health. 2019;24: 421–431. doi:10.1111/tmi.13205

23. Sharma M, Malhotra B, Tiwari J, Bhargava S. Profile of Nontuberculous Mycobacteria in Patients Suspected of Tuberculosis and Drug-Resistant Tuberculosis. J Lab Physicians. 2020;12: 203–211. doi:10.1055/s-0040-1721160

24. Liu CF, Song YM, He WC, Liu DX, He P, Bao JJ, et al. Nontuberculous mycobacteria in China: incidence and antimicrobial resistance spectrum from a nationwide survey. Infect Dis Poverty. 2021;10. doi:10.1186/s40249-021-00844-1

25. Hoefsloot W, Van Ingen J, Andrejak C, Ängeby K, Bauriaud R, Bemer P, et al. The geographic diversity of nontuberculous mycobacteria isolated from pulmonary samples: An NTM-NET collaborative study. European Respiratory Journal. 2013;42: 1604–1613. doi:10.1183/09031936.00149212

26. Okoi C, Anderson STB, Antonio M, Mulwa SN, Gehre F, Adetifa IMO. Non-tuberculous Mycobacteria isolated from Pulmonary samples in sub-Saharan Africa - A Systematic Review and Meta Analyses. Sci Rep. 2017;7. doi:10.1038/s41598-017-12175-z

27. Aliyu G, El-Kamary SS, Abimiku A, Brown C, Tracy K, Hungerford L, et al. Prevalence of Non-Tuberculous Mycobacterial Infections among Tuberculosis Suspects in Nigeria. PLoS One. 2013;8: e63170. doi:10.1371/journal.pone.0063170

28. Dinic L, Idigbe OE, Meloni S, Rawizza H, Akande P, Eisen G, et al. Sputum Smear Concentration May Misidentify Acid-Fast Bacilli As Mycobacterium Tuberculosis in HIV-Infected Patients. J Acquir Immune Defic Syndr. 2013;63: 168–177. doi:DOI:10.1097/QAI.0b013e31828983b9

29. Kennedy TP, Weber DJ. Nontuberculous mycobacteria. An underappreciated cause of geriatric lung disease. Am J Respir Crit Care Med. 1994;149: 1654–1658. doi:10.1164/ajrccm.149.6.8004325

30. Limo JJ, Nyerere A, Bii C. Prevalence, Species Characterization and factors associated with Non tuberculous mycobacteria among tuberculosis retreatment patients submitting samples to the National Tuberculosis Reference Laboratory, Kenya. Jomo Kenyatta University of Agriculture and Technology. 2016. Available: http://ir.jkuat.ac.ke

31. Xu J, Li P, Zheng S, Shu W, Pang Y. Prevalence and risk factors of pulmonary nontuberculous mycobacterial infections in the Zhejiang Province of China. Epidemiol Infect. 2019;147: e269. doi:10.1017/S0950268819001626

32. Corbett EL, Churchyard GJ, Clayton T, Herselman P, Williams B, Hayes R, et al. Risk Factors for Pulmonary Mycobacterial Disease in South African Gold Miners A Case-Control Study. Am J Respir Crit Care Med. 1999;159: 94–99. doi:https://doi.org/10.1164/ajrccm.159.1.9803048

33. Andréjak C, Nielsen R, Thomsen V, Duhaut P, Sørensen HT, Thomsen RW. Chronic respiratory disease, inhaled corticosteroids and risk of non-tuberculous mycobacteriosis. Thorax. 2013;68: 256–262. doi:10.1136/thoraxjnl-2012-201772

34. Feng JY, Chen WC, Chen YY, Su WJ. Clinical relevance and diagnosis of nontuberculous mycobacterial pulmonary disease in populations at risk. Journal of the Formosan Medical Association. 2020;119: S23–S31. doi:10.1016/j.jfma.2020.05.012

35. Hoza AS, Mfinanga SGM, Rodloff AC, Moser I, König B. Increased isolation of nontuberculous mycobacteria among TB suspects in Northeastern, Tanzania: Public health and diagnostic implications for control programmes. BMC Res Notes. 2016;9. doi:10.1186/s13104-016-1928-3

36. Corbett EL, Churchyard GJ, Hay M, Herselman P, Clayton T, Williams B, et al. The Impact of HIV Infection on Mycobacterium kansasii Disease in South African Gold Miners. Am J Respir Crit Care Med. 1999;160: 10–14. Available: www.atsjournals.org

37. Kone B, Sarro YS, Maiga M, Sanogo M, Somboro AM, Diarra B, et al. Clinical characteristics of non-tuberculous mycobacterial pulmonary infections in Bamako, Mali. Epidemiol Infect. 2018;146: 354–358. doi:10.1017/S0950268817003090

38. Jankovic M, Samarzija M, Sabol I, Jakopovic M, Katalinic Jankovic V, Zmak L, et al. Geographical distribution and clinical relevance of non-tuberculous mycobacteria in Croatia. International Journal of Tuberculosis and Lung Disease. 2013;17: 836–841. doi:10.5588/ijtld.12.0843

39. Sun Q, Yan J, Liao X, Wang C, Wang C, Jiang G, et al. Trends and Species Diversity of Non-tuberculous Mycobacteria Isolated From Respiratory Samples in Northern China, 2014–2021. Front Public Health. 2022;10: 1–8. doi:10.3389/fpubh.2022.923968

40. Farnia P, Farnia P, Ghanavi J, Velayati AA. Epidemiological Distribution of Nontuberculous Mycobacteria Using Geographical Information System. Nontuberculous Mycobacteria (NTM): Microbiological, Clinical and Geographical Distribution. Elsevier; 2019. pp. 191–321. doi:10.1016/B978-0-12-814692-7.00010-3

41. Gonzalo-Asensio J, Pérez I, Aguiló N, Uranga S, Picó A, Lampreave C, et al. New insights into the transposition mechanisms of IS6110 and its dynamic distribution between Mycobacterium tuberculosis Complex lineages. PLoS Genet. 2018;14. doi:10.1371/journal.pgen.1007282

42. Lira LAS, Santos FCF, Carvalho MSZ, Montenegro RA, Lima JFC, Schindler HC, et al. Evaluation of a IS6110-Taqman real-time PCR assay to detect Mycobacterium tuberculosis in sputum samples of patients with pulmonary TB. J Appl Microbiol. 2013;114: 1103–1108. doi:10.1111/jam.12119

43. Hemeg HA, Albulushi HO, Ozbak HA, Ali HM, Alahmadi EK, Almutawif YA, et al. Evaluating the Sensitivity of Different Molecular Techniques for Detecting Mycobacterium tuberculosis Complex in Patients with Pulmonary Infection. Pol J Microbiol. 2023;72: 421–431. doi:10.33073/pjm-2023-040

44. Hanifa Y, Fielding KL, Chihota VN, Adonis L, Charalambous S, Foster N, et al. The utility of repeat Xpert MTB/RIF testing to diagnose tuberculosis in HIV-positive adults with initial negative result. Gates Open Res. 2018;2. doi:10.12688/gatesopenres.12815.1

45. Kolia-Diafouka P, Carrère-Kremer S, Lounnas M, Bourdin A, Kremer L, Van de Perre P, et al. Detection of Mycobacterium tuberculosis in paucibacillary sputum: performances of the Xpert MTB/RIF ultra compared to the Xpert MTB/RIF, and IS6110 PCR. Diagn Microbiol Infect Dis. 2019;94: 365–370. doi:10.1016/j.diagmicrobio.2019.02.008

46. Hamdi A, Fida M, Deml SM, Abu Saleh O, Wengenack NL. Utility of Mycobacterium tuberculosis PCR in ruling out active disease and impact on isolation requirements in a low prevalence setting. J Clin Tuberc Other Mycobact Dis. 2020;21: 1–5. doi:10.1016/j.jctube.2020.100181

47. Fang Y, Wu WH, Pepper JL, Larsen JL, Marras SAE, Nelson EA, et al. Comparison of real-time, quantitative PCR with molecular beacons to nested PCR and culture methods for detection of Mycobacterium avium subsp. paratuberculosis in bovine fecal samples. J Clin Microbiol. 2002;40: 287–291. doi:10.1128/JCM.40.1.287-291.2002

48. Seo AN, Park HJ, Lee HS, Park JO, Chang HE, Nam KH, et al. Performance characteristics of nested polymerase chain reaction vs real-time polymerase chain reaction methods for detecting mycobacterium tuberculosis complex in paraffin-embedded human tissues. Am J Clin Pathol. 2014;142: 384–390. doi:10.1309/AJCP2QZRH4ZNPRDD

49. Dowling WB, Whitelaw A, Nel P. Tracing TB: Are there predictors for active TB disease in patients with Xpert Ultra trace results? International Journal of Infectious Diseases. 2022;114: 115–123. doi:10.1016/j.ijid.2021.10.056

50. Boyles TH, Hughes J, Cox V, Burton R, Meintjes G, Mendelson M. False-positive Xpert® MTB/RIF assays in previously treated patients: Need for caution in interpreting results. International Journal of Tuberculosis and Lung Disease. 2014;18: 876–878. doi:10.5588/ijtld.13.0853

Sequencing results for the identification of NTM species

Click here to access/download;Figure;Fig 2.tif

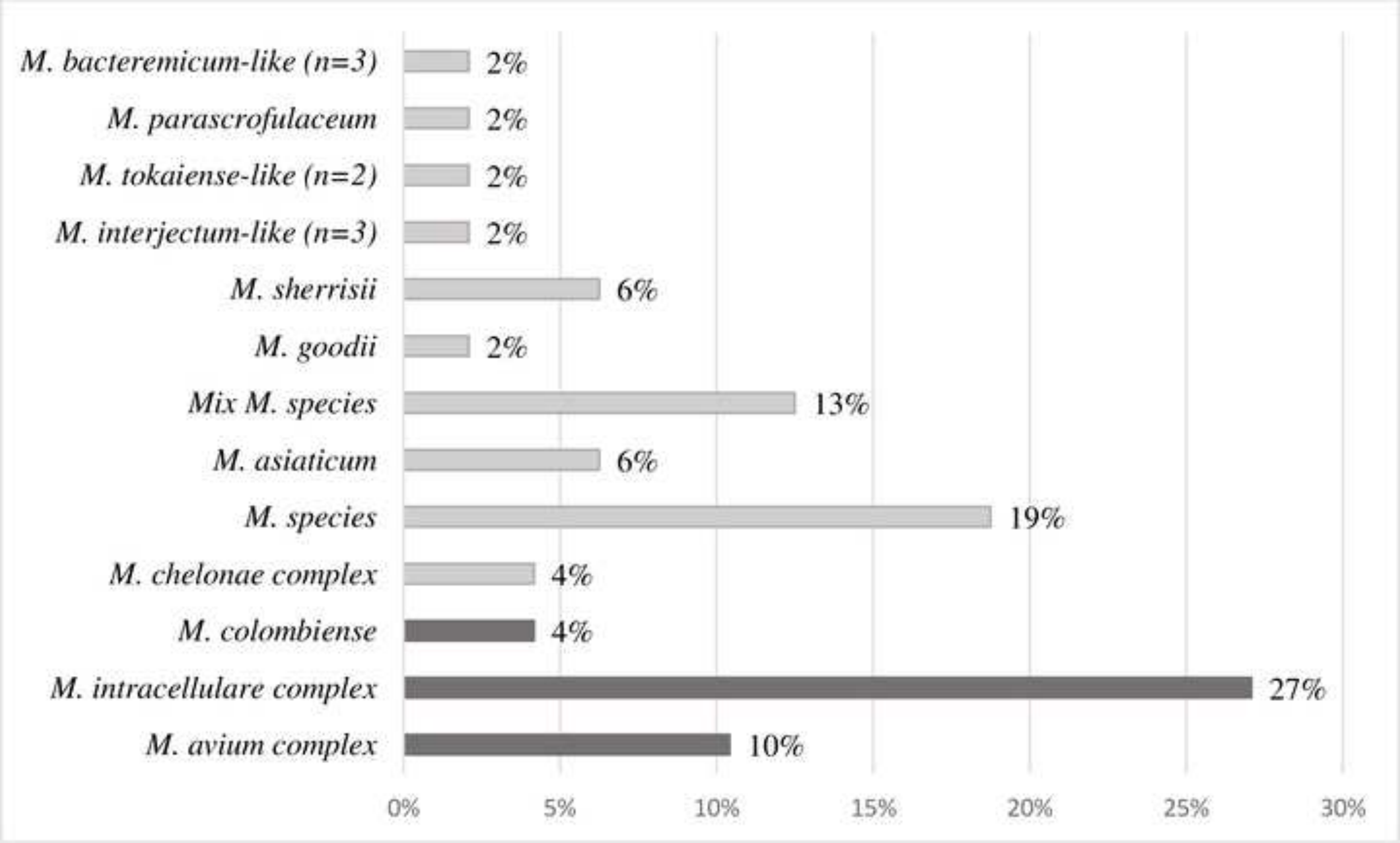

Sample and analysis flow with summary of results

Click here to access/download;Figure;Fig 1.tif

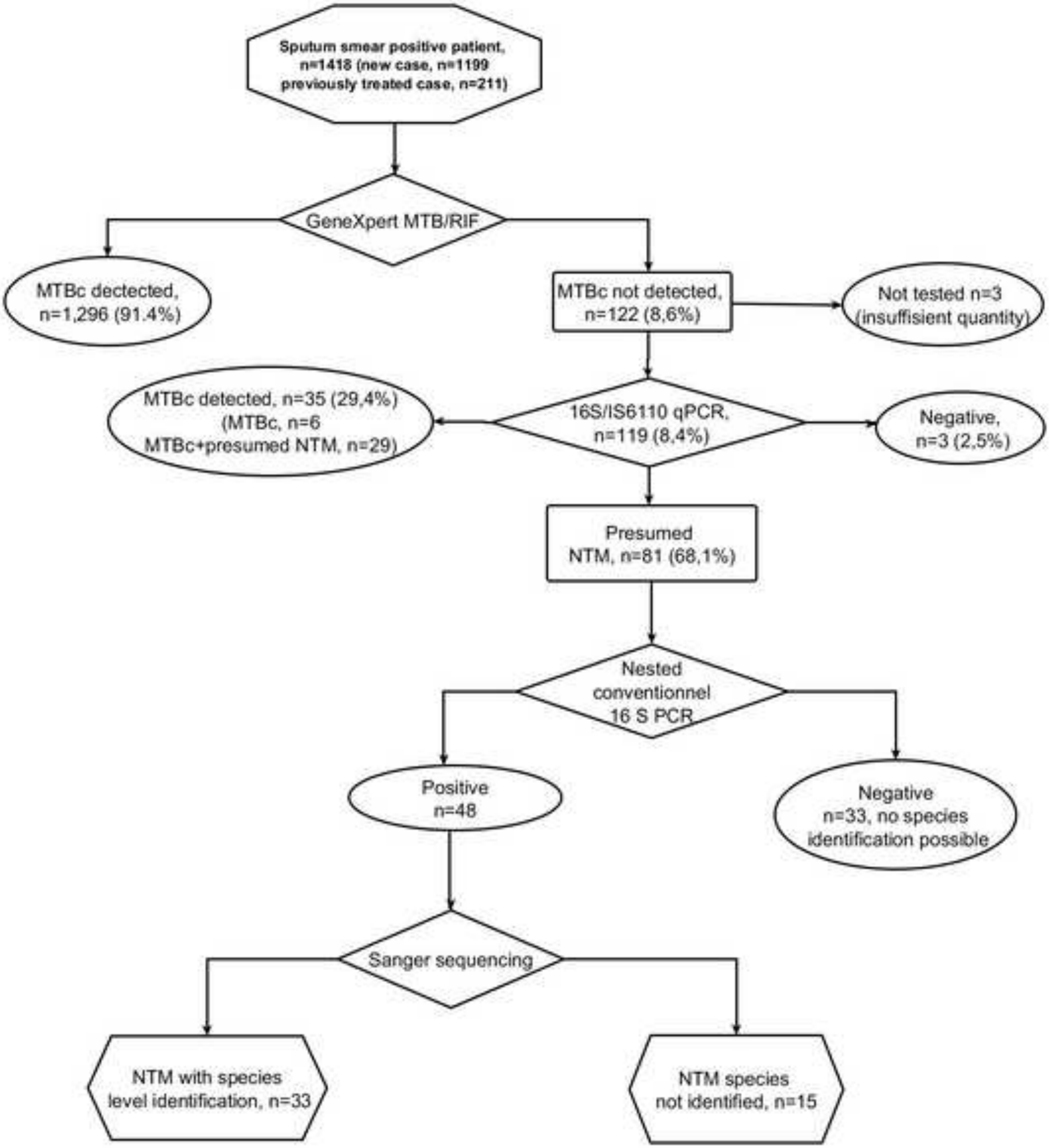

Minimal data set

Click here to access/download
Supporting Information
Minimal data set.csv

Minimal data set

Click here to access/download
Supporting Information
Minimal data set.mat

Prevalence of non-tuberculous mycobacteria among people with acid-fast positive presumptive tuberculosis in Mali

Prevalence of non-tuberculous mycobacteria among people with presumptive tuberculosis in Mali

Aissata Boubakar CISSE[1]*, Anna S. DEAN[2], Armand Van DEUN[3†], Jelle KEYSERS[3], Willem-Bram De RIJK[3], Mourad GUMUSBOGA[3], Hawa SAMAKE[1], Seydou ARAMA[1], Bassirou DIARRA[5], Ibrahim DJILLA[1], Fatoumata N. COULIBALY[1], Hawa SIMPARA[1], Mamadou BERTHE[1], Khadidia OUATTARA[6], Yacouba TOLOBA[6], Ibrehima GUINDO[1], Bouke de JONG[3], Leen RIGOUTS[3,4].

1- National Institute of Public Health, Bamako, Mali
2- Word Health Organization, Geneva
3- Mycobacteriology Unit, Institute of Tropical Medicine, Antwerp, Belgium
4- Belgian Coordinated Collections of Microorganisms, Antwerp, Belgium
5- University Clinical Research Center, Bamako, Mali
6- University Teaching hospital of Point G, Bamako, Mali

† Deceased in September 2023

* Corresponding author

E-mail: aissatacisse@yahoo.fr (ABC)

Abstract

Background: Non-tuberculous mycobacteria (NTM) are environmental agents that can cause opportunistic pulmonary disease in humans and animals, often misdiagnosed as tuberculosis (TB). In this study, we describe the cases of NTM identified during the first national anti-TB drug resistance survey conducted in Mali and ~~explored~~explore associated risk factors.

Methods: Sputum was collected from people presenting for pulmonary TB diagnosis from April to December 2019, regardless of age. Microscopy-positive patients were enrolled and tested using the Xpert MTB/RIF assay. A ~~P~~patient who tested negative for the *Mycobacterium tuberculosis* complex (MTBC) was tested for the presence of mycobacteria by amplification of the IS*6110* and 16SrRNA (16S) genes through double quantitative real-time PCR, followed by nested PCR and Sanger sequencing of the IS*6110*-negative samples for NTM species identification.

Results: 1,418 sputum smear-positive patients were enrolled, including 1,199 new cases, 211 previously treated cases, and 8 whose previous treatment history was unknown. Based on the results of Xpert MTB/RIF assay and in-house PCR methods, 1,331 (93.9%) patients were positive for ~~MTBc~~MTBC, 48 (3.4%) for NTM, and no species identification was possible for 39 (2.7%). Advanced age of 65 and over with an OR 8.8 (95% CI 2.3-33.2 and p=0.001) and previous TB treatment with an OR 3.4 (95% CI 1.2-9.6 and p=0.016) were the risk factors statistically associated with NTM detection. *M. avium complex* (MAC) was the predominant NTM species, detected in 20 cases.

Conclusion: Detection of NTM in people presumed to have TB is an ongoing challenge, confounding correct TB diagnosis. Concomitant use of microscopy and GeneXpert testing among at-risk individuals could improve patient management.

1. Introduction

The *Mycobacterium* genus groups species of the *Mycobacterium tuberculosis* complex (MTBC) responsible for tuberculosis (TB) in humans and animals, *M. leprae*, the causative agent of leprosy, and the species of mycobacteria commonly known as atypical or non-tuberculous mycobacteria (NTM), including *M. ulcerans* responsible for Buruli ulcer. Unlike members of the MTBCe group, NTM are not obligatory parasites of humans, yet normal residents of soil and water, and can be found in natural and treated water sources [1]. More than 200 species of NTM have been officially recognised [2], of which around 25 are known to be strongly associated with disease in humans. Some species have been associated with pulmonary diseases causing symptoms similar to TB [1]. Because of their habitat, humans are exposed to these bacteria daily. Therefore, NTM disease must, therefore, be distinguished from simple colonisation or contamination of clinical samples, for example from tap water [1,3]. Unlike TB, the global epidemiology of disease caused by NTM is not well known. NTM isolation from clinical specimens is mainly reported in industrialised countries, with variable prevalence and incidence. Studies based on pulmonary specimen isolates reported a prevalence of 1.4 to 6.6 per 100,000 persons in the USA from 2004 to 2006 [4], 9.8 in 2010 in Ontario, Canada [5], and 5.8 per 100,000 in Germany [6] in 2020. Incidence of 6.1 per 100,000 in 2012 in England [7] and 5.3 per 100, 000 in Germany in 2020 [6] have also reported. In TB-endemic countries, NTM is being reported less frequently and mainly in populations at risk, particularly people with predisposing

conditions or compromised immunity [8]. However, experiences in industrialised countries have shown that a declining TB burden has also increased in the number of NTM cases detected. With the strengthening of TB programmes in another setting, we may also anticipate a similar scenario, posing a growing diagnosis and clinical challenge in low-and middle-income countries [9]. The diagnosis of NTM lung disease is based on clinical, radiological, and microbiological criteria [1]. Laboratory-based detection of NTM, with differentiation from MTBC and identification to the species level, is mainly inaccessible in most resource-limited countries. Microscopy, the most accessible technique, identifies MTBC and NTM as acid-fast bacilli (AFB) but fails to distinguish them. Since 2010, the World Health Organization (WHO) has recommended rapid molecular tests such as GeneXpert MTB/RIF (Xpert) as the initial test for TB diagnosis, exhibiting a higher sensitivity and specificity [10]. This assay only identifies the presence of MTBC species in the sample. A negative result for ~~MTBc~~ MTBC by Xpert obtained on AFB-positive sputum could~~, therefore, be~~ indicate NTM [11]. In Mali, cases of NTM infection have been described, particularly in patients under investigation for failure of anti-TB treatment or who have relapsed after TB cure [12]. Following the introduction of Xpert in the country, plausible cases of NTM infection with an AFB-positive smear and a negative Xpert have been reported more frequently [13].

In this study, we describe the proportion of MTBC and NTM detected in patients presenting for initial or previously treated TB diagnoses in Mali~~,~~ in the context of a nationwide drug resistance survey [14]. We also explored risk factors associated with NTM detection.

2. Methods

2.1. Design of the study

From April to December 2019, consecutive eligible patients were recruited from 78 of 86 TB diagnostic laboratories, representing 91% of the country's TB laboratory network. Eight laboratories notified less than 10 new bacteriologically confirmed pulmonary TB cases in 2017 and were not included for logistic reasons. Eligible participants were defined as people undergoing evaluation for pulmonary TB, regardless of age, with and without a ~~history of~~ prior treatment history. The sample size of 994 new cases was calculated to meet the primary objective of the survey for measuring the percentage of new pulmonary TB cases resistant to rifampicin, for which a recruitment period of 4 months was estimated to be required [15].

2.2. Sample collection and patient enrolment

Two sputum samples of approximately 5 mL were collected from each patient in the participating laboratories. Smears were made directly from freshly collected sputum, stained according to the acid-fast staining technique used at the center (auramine for most sites and Ziehl-Neelsen for several small centers), and in line with international guidelines for diagnosing TB by microscopy [16]. If one of these two sputum samples was AFB positive, the patient was included in the drug resistance survey after giving informed consent, and an additional sample was collected.

The nurse completed the study questionnaire with the recruited patient. The questionnaire covered socio-demographic data, ~~and~~ information on previous TB treatment, and pulmonary disease history (duration and chronicity of respiratory signs). Relevant clinical and treatment-

related information was also provided to the patient. All enrolled participants received anti-TB treatment. New patients were started on first-line TB treatment based on AFB microscopy. According to national guidelines, treatments were initiated based on the Xpert MTB/RIF testing results of patients with previously treated TB.

2.3. Initial GeneXpert testing and specimen storage

The first two sputum samples collected were preserved in 5 mL of OMNIgene® SPUTUM (OM-S) decontamination reagent (DNA Genotek Inc, Canada) according to the manufacturer's recommendations [17]. In contrast, the third sample was preserved in 10 mL of 96% ethanol. The conditioned samples were transported by public transport at ambient temperature in triple packaging to the National TB Reference Laboratory (NTRL) in Bamako.

The Xpert MTB/RIF assay (Cepheid, CA, USA) was performed at the NTRL using 2 mL of one OM-S-preserved sample, as per the manufacturer's recommendations (Cepheid and Genotek).

Leftovers of the OM-S-preserved samples were then stored in cryotubes as sediment obtained after centrifugation at 4000 g for 15 minutes and mixed with 96% alcohol at an equal volume (0.5 mL sediment and 0.5 mL ethanol).

2.4. Molecular detection and identification of NTM species

For molecular detection and identification of NTM species, according to the study protocol, samples from the following patients were transported at ambient temperature to the WHO Supranational TB Reference Laboratory at the Institute of Tropical Medicine (ITM) in Antwerp, Belgium:

- cases with rifampicin resistance by Xpert MTB/RIF, regardless of anti-TB treatment history;
- all previously treated cases, regardless of Xpert MTB/RIF test result;
- and cases for whom ~~MTBc~~ MTBC was not detected by Xpert MTB/RIF.

In this article, we describe only the molecular methods employed and the results obtained for species identification of probable NTM.

Upon arrival at ITM, ethanol-preserved samples were centrifuged at 4000 g for 15 minutes, digested by overnight proteinase K incubation, followed by lysis using an internal lysis buffer, after which DNA was extracted using the Modified Maxwell-LEV® automated extraction method (Promega, USA) [18]. A duplex quantitative real-time polymerase chain reaction (qPCR) was performed, targeting the IS*6110* gene to detect the MTBC and the 16S gene to detect all species of the *Mycobacterium* genus. The primers and probes used are presented in Table 1, while the interpretation of results is depicted in Table 2.

Table 1. Primers and probes for qPCR to simultaneously detect *Mycobacterium tuberculosis* complex and all mycobacterial species.

Target	Name	Sequences
**IS*6110* gene**	IS*6110*_S	5'-CCTGAAAGACGTTATCCACCATAC-3'
	IS*6110*_AS	5'-GGC-TAG-TGC-ATT-GTC-ATA-GGA-G-3'
	IS6110_P_DFO	DFO-TCT-CAG-TA(lnC)-AC(lnA)-TC(lnG)-ATC-CGG-T-BHQ2
**16S gene**	Myco_Genus_S_v3	5'-ATGCAAGTCGAACGGAAAGG-3'
	Myco_Genus_AS_v2	5'-TCTGCCCGTATCGCC-C-3'
	Myco_Genus_P_v2	FAM-CAA-AC(lnC)-AC(lnC)-TA(lnC)-GA(lnG)-CTC-EDQ

Table 2. Interpretation of duplex IS*6110*-16S qPCR results

**16S qPCR Cycle threshold (Ct)**	**IS*6110* qPCR result**	
	**Positive**	**Negative**
**Positive with any Ct value**	-	No ~~MTBc~~MTBC
**Positive and ≥IS*6110* Ct**	~~MTBc~~MTBC	
**Positive and <IS*6110* Ct**	~~MTBc~~ MTBC + NTM	
**Negative**	~~MTBc~~MTBC (low bacillary load)	No mycobacteria species identification possible

~~MTBc~~ MTBC = *Mycobacterium tuberculosis* complex; NTM = non-tuberculous mycobacteria: Ct = Cycle threshold

Subsequently, a nested conventional PCR targeting the 16S gene was performed on samples identified as NTM by the duplex qPCR. The primers and probes used were as follows:

- P1 (first PCR): 5'-TGCTTAACACATGCAAGTCG-3'
- P2 new (first PCR): 5'-TCTCTAGACGCGTCCTGTGC-3'
- P7 (nested PCR): 5'-CATGCAAGTCGAACGGAAAGG-3'
- P16 new (nested PCR): 5'-AAGCCGTGAGATTTCACGAACA3'

Amplicons that were positive on the agarose gel underwent Sanger sequencing. A negative PCR result was interpreted as the absence of ~~MTBc~~ MTBC or NTM.

Sequencing was subcontracted to BaseClear (BE Leiden, The Netherlands). After entering the sequence into the Basic Local Alignment Search Tool (BLAST) of the National Centre for Biotechnology Information (NCBI), we compared it to reference strains sequences on NCBI such as ATCC 25791, ATCC BAA-614, ATCC 27282, ATCC 51457, 4773, ATCC 700504, ATCC 25276, ATCC 35752, ATCC 13950. We interpreted the results obtained as follows:

- If a sequence match was found on the NCBI, the NTM species was identified.

    - In case of 100% similarity (no mismatches), the species name was assigned.
    - In case 1-3 bp mismatches with the closest species, the “species-like” name was assigned.
- If no match or >3 bp difference with the closest species was found on NCBI, the result was interpreted as *Mycobacterium* species.
- If there were overlapping peaks in the sequence, indicating the presence of several NTM, the result was interpreted as "Mixture of NTM ".

2.5. Data analysis

The data collected using the survey and sample submission forms were entered into EpiInfo version 7.2.5.0. The variables analysed were age, sex, region of residence, history of illness, and history of anti-TB treatment. Percentages, medians, and means were calculated by crosstab, and p-values and odds ratios with 95% confidence intervals were generated using regression analysis in IBM SPSS Statistics version 21X64 (IBM Corp., NY, USA).

2.6. Ethical considerations

The drug resistance survey protocol in which a secondary analysis of samples is described in the informed consent was accepted by the National Institute of Public Health Research ethics committee by decision No. 08/2018/CE-INRSP on March 21, 2018.The objectives and expected results of the study were clearly explained in the language understood by the patient. Patients were asked permission to use their samples for additional analyses, including outside Mali. Oral and written consent were obtained from patients over 18 years of age. For patients under the age of 18, after obtaining the prior oral and written consent of the parent or companion, the child's

assent to participate in the study was obtained in the presence of a witness other than the parent or companion.

3. Results

3.1. Mycobacteria detected

A total of 1,418 AFB-positive people with presumed pulmonary TB were enrolled in the drug resistance survey, including 1,199 new cases, 211 previously treated cases, and 8 whose previous treatment history was unknown (Figure 1). Based on Xpert MTB/RIF results, 1,296 (91.4%) patients were positive for ~~MTBc~~MTBC, while ~~1MTBc~~ 1MTBC was not detected for 22 (8.6%). The duplex IS6110-16S qPCR performed for 119 of these 122 patients identified another 35 ~~MTBc~~MTBC, bringing the total number of patients with ~~MTBc~~ MTBC to 1,331 (93.9%). Of these 35 additional cases, ~~MTBc~~ MTBC alone was detected for six, while 29 carried probable mixtures of ~~MTBc~~ MTBC and NTM.

Fig 1. Sample and analysis flow with summary of results, ~~MTBc~~MTBC=*Mycobacterium tuberculosis* complex; n= number; NTM = non-tuberculous mycobacteria.

For 81 (68,1%) of 119 patients tested, only the 16S target reacted positively (presumed NTM), while the PCR remained negative for both targets in 3 (2,5%) patients. The nested conventional 16S PCR performed for the 81 presumed NTM detected by IS*6110*-16S qPCR was positive for 48 or 3.4% (48/1418) of all enrolled patients, of which 33 could be identified to the species level by sequencing. The remaining 33 of 81 presumed NTM had a negative nested PCR result, thus no sequencing could be performed (Fig 1).

Thus, eleven species or groups of species could be identified, with a predominance of the *M. avium* complex (MAC) representing 20 cases or 42% of all species detected, *M. intracellulare* complex in 13 patients (27%), *M. avium* in five (10%) and *M. colombiense* in two (4 %) (Fig 2).

Fig 2. Sequencing results for the identification of NTM species

3.2. Detection of NTM in relation to smear-microscopy results

The participating laboratories were notified of the quantification of AFB for 1414 of 1418 patients. A significantly higher proportion of NTM was detected in patients with lower AFB-grades: 7% for 1+ and scanty (Table 3), compared to 2% among 3+ patients (respectively OR 4.065, 95% CI: 1.876-8.808, p=0.000 and OR 4.3, 95% CI: 2.012-9.189, p=0.000). The difference was not significant compared to grade 2+ samples (p=0.462).

Table 3. Proportion of NTM and MTB detection in relation to quantitative sputum-smear microscopy results

Final sputum smear result	~~MTBc~~MTBC n (%)	NTM n (%)	No species identification possible n (%)	Total n (%)
**Scanty**	167 (83)	13 (7)	21 (10)	201
**1+**	170 (89)	14 (7)	8 (4)	192
**2+**	259 (94)	7 (3)	8 (3)	274
**3+**	731 (98)	14 (2)	2 (0)	747
**Not quantified**	0	0	4 (100)	4
**Total**	**1327 (93.6)**	**48 (3.4)**	**43 (3)**	**1418 (100)**

~~MTBc~~ MTBC = *M. tuberculosis* complex; NTM = non-tuberculous mycobacteria; n =number

3.3. Socio-demographic, clinical characteristics and risk factors for NTM infection

Male patients were more common among study participants and among those with identified NTM, with a sex ratio of respectively 2.1 and 1.5 when compared to women, yet NTM presence was not associated with gender (Table 4). On the contrary, older age was significantly associated with NTM detection. The median age was 37 years for all patients and 59.5 years for those with NTM detected. Compared to the under-34 age group, the 35-64 age group had greater odds (OR 3.4, 95% CI: 1.1-10.3, p=0.03) of NTM detection, which further increased for the over-65 age group (OR 8.8, 95% CI: 2.3-33.2 p=0.001 (Table 4). Also, previously treated patients were more likely than new cases to have NTM in their sputum (OR 3.4, 95% CI: 1.2-9.6, p=0.016) (Table 4). We found no significant associations between NTM detection and HIV status or chronic respiratory signs. Regarding the patient's residence, patients from the Mopti region were more likely to have NTM detected than Bamako residents (OR 4.1, 95% CI: 1.0-16.4, p=0.04). (Table 4).

Table 4. Multivariate logistic regression analyses of demographic and clinical variables as risk factors of NTM infection

Variables		Total patients n (%)	NTM detected n (%)	OR	p-value
**Gender n=1379**	Female	434 (31.5)	19 (39.6%)	0.8 [0.3-2.04]	0.7
	Male	945 (68.5)	29 (60.4%)		
**Age n=1379**	Median	37	**59,5**		
**Age group**	0-34 years (Reference)	603 (43.7)	4 (8.3)		
	35-64 years	659 (47.8)	29 (60.4)	**3.4 [1.1-10.3]**	**0.03**
	≥65 years	117 (8.5)	15 (31.3)	**8.8 [2.3-33.2]**	**0.001**
**History of treatment n=1371**	New	1165 (84.5)	30 (62.5)		
	Previously treatment	206 (14.9)	18 (37.5)	**3.4 [1.2-9.6]**	**0.016**
**HIV status n=1092**	Positive	77 (5.6)	4 (8.3)	2.8 [0.8-8.9]	0.07
	Negative	1015 (73.6)	29 (60.4)		
**Chronic clinical signs n=1174**	Yes	442 (37.6)	24 (60)	0.7 [0.3-1.8]	0.5
	No	752 (62.4)	16 (40)		
**Region of residence at the time of diagnosis n=1348**	Bamako (Reference)	451 (33.5)	5 (10.9)		
	Kayes	101 (7.5)	4 (8.7)	2.7 [0.5-12.7]	0.2
	Koulikoro	217 (16.1)	7 (15.2)	1.5 [0.3-7.4]	0.5
	Sikasso	155 (11.5)	12 (26.1)	2.9 [0.7-10.8]	0.10
	Ségou	147 (10.9)	7 (15.2)	3.2 [0.8-12.7]	0.09
	Mopti	191 (14.2)	7 (15.2)	**4.1 [1.02-16.4]**	**0.04**
	Northern regions	86 (6.4)	4 (8.7)	4.2 [0.7-25.2]	0.1

NTM = non-tuberculous mycobacteria; n = number; OR = odds ratio

4. Discussion

The proportion of sputum-AFB-positive patients and ~~MTBc~~ MTBC detected by Xpert MTB/RIF among people presumed to have TB in our study was slightly lower than the rate reported in other drug resistance surveys, ranging from 95.8%-97.7% [19–22]. The confirmed proportion with NTM in our study was higher than in TB drug-resistance surveys in Pakistan (0.7%, 13/1972) [19] and in India (1.2%, 35/2,938) [23], yet lower than the 6.4% (317/4917) observed in China [24]. This result may reflect regional differences in the occurrence of these mycobacteria ~~occurrence~~ and the diseases they cause ~~around the world~~worldwide [25,26].

NTM detection was more likely among paucibacillary patients as determined by smear microscopy, corroborating findings from Nigeria where the AFB-smear grading was relatively lower for patients with NTM detected than for those with ~~MTBc~~ MTBC [27], and higher AFB grading was predictive for ~~MTBc~~ MTBC detection [28]. With age, the risk of developing NTM infection increases~~d~~. Still, we did not find a significant association between gender and NTM infection;~~,~~ contrary to other studies, ~~where~~ females have been more frequently associated with NTM infections, especially at older ages [11,29,30]. Previous anti-TB treatment was another risk factor for NTM infection in our study, confirming findings from NTM pulmonary disease in China and South Africa [31,32]. Nevertheless, we did not find an association between NTM detection and chronic respiratory signs in our study, in contrast with the described association between NTM infection and lung disease or chronic respiratory symptoms in studies from China and Denmark [33,34].

HIV infection has frequently been identified as a risk factor for NTM infections and disease, especially in situations of advanced immunosuppression [27,35,36]. In our study, the trend toward an association between HIV positivity and NTM detection was not significant,

corroborating previous observations in Mali [37]. ~~In the drug resistance survey context, no~~ No information about ~~on~~ anti-retroviral treatment was ~~collected~~gathered as part of the drug resistance survey. Still, such treatment is to be started upon HIV diagnosis as per national guidelines in Mali. While patients from the Mopti region had higher odds of having NTM detected in their sputum than those from the Bamako region, further investigation of the environmental conditions in these regions would be needed, especially as NTM are environmental bacteria and have often been isolated at different frequencies in different geographical areas [25,30,38]. MAC species were the most commonly NTM detected in our survey. The predominance of MAC has been reported in pulmonary NTM infections in many countries around the world [25,26,39,40]. In Mali, between 2004 and 2013, *M. avium* was the most frequently identified species of the MAC complex [12,37].

Employing an additional in-house qPCR for IS*6110* in our study has ~~allowed to increase~~increased the detection of MTBC. The MTBC~~-~~specific repetitive element IS*6110* is a suitable target for MTBC detection assays [41]. The applied qPCR IS*6110* has shown good sensitivity and specificity for detecting MTBC compared to culture [42] and higher performance when compared to the Xpert MTB/RIF assay [43]. The observed high Ct values in our study indicate, however, a low quantity of MTB DNA in samples from these patients, which may have been positive upon repeated Xpert testing [44] or by using the Xpert Ultra test, which is more sensitive by targeting both IS*6110* and another repetitive element IS*1081* for MTBC detection, in addition to the *rpoB* gene for assessing rifampicin susceptibility [45]. The higher positivity rate by qPCR compared to the conventional nested PCR we observed in our study could be partially attributable to the non-specificity of the 16S primers employed for the in-house qPCR, which also detects species belonging to closely related genera like *Rhodococcus* and *Nocardia*, hence

potentially overestimating the NTM rate. These closely related species can indeed be detected in smear-positive samples not containing tubercle bacilli, such as documented in the USA with 78.4% (134/171) of AFB-positive specimens yielding a positive culture with NTM or *Nocardia* spp, while 14.6% (25/171) of AFB-positive specimens grew MTBc MTBC and another 7% (12/171) remained negative in culture [46]. In another study in Nigeria, out of 32 smear-positive samples identified as not containing TB bacilli, six were NTM (18.75%), 14 were corynebacteria (43.75%), and 12 were rhodococci (37.5%) [28]. Our study did not further identify the non-MTBC/non-NTM samples. In addition, the lower sensitivity of the conventional nested PCR may have hampered confirmation by sequencing in case of negative results or low amplicon yield. Indeed, the in-house qPCR has already shown good sensitivity, while limited loss of DNA has been described during multiple handling steps for conventional nested PCR [47,48]. This lower sensitivity is reflected in the sample's AFB grading, with fewer non-identified samples among the strong positive specimens.

This study was part of the national TB drug resistance survey, comprising new and previously treated cases. The concern of false-positive MTBc MTBC detection in sputum from previously treated patients has been chiefly raised for the highly sensitive, newer generation of rapid molecular assays, such as the "MTB detected Trace" result by Xpert Ultra [49,50]. Nevertheless, even smear microscopy can still show non-viable AFB, and the introduction of alternative/additional diagnostic assays or flows would need to be validated against culture.

Our survey was not designed to include culture in the diagnostic algorithm, and hence, we could not assess the viability of the detected MTBC and NTM bacilli, nor whether co-detection of MTBC and NTM reflected simultaneous infection, or rather an NTM infection following MTBC infection in previously treated patients, which was still detectable due to remnant DNA.

Also, we did not investigate whether the detected NTM was attributable to actual infection, colonization, or sample contamination. We could not assess the American Thoracic Society criteria to detect NTM in at least 3 different samples per patient, given the design of the survey and the retrospective nature of molecular analysis to detect NTM. However, study participants were people with respiratory signs undergoing evaluation for pulmonary TB attending health centers or patients failing TB treatment. Finally, we do not describe the outcome of patients in whom NTM was detected. Still, all were started on 1st line TB therapy or multidrug-resistant treatment following the results of initial microscopy and Xpert testing.

5. Conclusion

Among sputum-AFB-positive patients presenting in Mali, 3.4% were due to NTM. Particular attention must be paid to populations at risk, such as elderly patients and patients with a history of previous treatment. TB prevention and care programmes in resource-limited countries should ensure that their routine diagnostic algorithms can detect such cases, based on the concurrent use of rapid molecular TB diagnostics and microscopy without other molecular assays to detect NTM. However, culture is still necessary to isolate these mycobacteria and to monitor treatment in the event of confirmation of NTM disease. Clinical assessment and management of NTM infections remain a challenge worldwide.

Acknowledgements

We would like to thank the TB management sites for recruiting patients, collecting and transporting samples, the country's regional health departments for regional coordination of the

drug resistance study, and Olga Tosas AUGUET for her assistance in planning the survey, monitoring the field activities and preliminary data analysis”.

References

1. Griffith DE, Aksamit T, Brown-Elliott BA, Catanzaro A, Daley C, Gordin F, et al. An official ATS/IDSA statement: Diagnosis, treatment, and prevention of nontuberculous mycobacterial diseases. Am J Respir Crit Care Med. 2007;175: 367–416. doi:10.1164/rccm.200604-571ST

2. Pascapurnama DN, Zavitri NG, Koesoemadinata RC, Cahyadi AI, Chaidir L. Identification of Significant Pathogenic Nontuberculous Mycobacteria Species from Presumptive TB Patients Using Partial hsp65 Gene Sequencing. Infect Drug Resist. 2023;16. doi:10.2147/IDR.S419956

3. Haworth CS, Banks J, Capstick T, Fisher AJ, Gorsuch T, Laurenson IF, et al. British Thoracic Society guidelines for the management of non-tuberculous mycobacterial pulmonary disease (NTM-PD). Thorax. 2017;72: iii1-ii64. doi:10.1136/thoraxjnl-2017-210927

4. Prevots DR, Shaw PA, Strickland D, Jackson LA, Raebel MA, Blosky MA, et al. Nontuberculous mycobacterial lung disease prevalence at four integrated health care delivery systems. Am J Respir Crit Care Med. 2010;182: 970–976. doi:10.1164/rccm.201002-0310OC

5. Marras TK, Mendelson D, Marchand-Austin A, May K, Jamieson FB. Pulmonary nontuberculous mycobacterial disease, Ontario, Canada, 1998-2010. Emerg Infect Dis. 2013;19: 1889–1891. doi:10.3201/eid1911.130737

6. Corbett C, Finger P, Heiß-Neumann M, Bohnert J, Eder IB, Eisele M, et al. Development of prevalence and incidence of non-tuberculous mycobacteria in German laboratories from 2016 to 2020. Emerg Microbes Infect. 2023;12. doi:10.1080/22221751.2023.2276342

7. Shah NM, Davidson JA, Anderson LF, Lalor MK, Kim J, Thomas HL, et al. Pulmonary Mycobacterium avium-intracellulare is the main driver of the rise in non-tuberculous mycobacteria incidence in England, Wales and Northern Ireland, 2007-2012. BMC Infect Dis. 2016;16. doi:10.1186/s12879-016-1521-3

8. Varma-Basil M, Bose M. Mapping the Footprints of Nontuberculous Mycobacteria. Nontuberculous Mycobacteria (NTM): Microbiological, Clinical and Geographical Distribution. Elsevier; 2019. pp. 155–175. doi:10.1016/B978-0-12-814692-7.00008-5

9. Iseman MD, Marras TK. The importance of nontuberculous mycobacterial lung disease. Am J Respir Crit Care Med. 2008;178: 999–1000. doi:10.1164/rccm.200808-1258ED

10. WHO. Module 3: Diagnosis WHO operational handbook on tuberculosis Rapid diagnostics for tuberculosis detection. 2021.

11. Kong L, Xie B, Liu Q, Hua L, Bhusal A, Bao C, et al. Application of acid-fast staining combined with GeneXpert MTB/RIF in the diagnosis of non-tuberculous mycobacteria pulmonary disease. International Journal of Infectious Diseases. 2021;104: 711–717. doi:10.1016/j.ijid.2020.12.091

12. Maiga M, Siddiqui S, Diallo S, Diarra B, Traoré B, Shea YR, et al. Failure to recognize nontuberculous mycobacteria leads to misdiagnosis of chronic pulmonary tuberculosis. PLoS One. 2012;7: e36902. doi:10.1371/journal.pone.0036902

13. Toloba Y, Cissé AB, Soumaré D, Ouattara K, Kanouté T, Koné D, et al. Apport du GeneXpert dans le diagnostic de la tuberculose et de la résistance à la rifampicine au Mali après 22 mois d'acquisition. Rev Mal Respir. 2017;34: A216–A217. doi:10.1016/j.rmr.2016.10.515

14. World Health Organization. Global tuberculosis report 2020. World Health Organization; 2020.

15. World Health Organization. Guidelines for surveillance of drug resistance in tuberculosis 5 th Edition. 2015.

16. Lumb R, Van Deun A, Bastian I, Fitz-Gerald M, Pathology SA, Stop TB Partnership (World Health Organization). Laboratory diagnosis of tuberculosis by sputum microscopy : the handbook. 2013.

17. DNA Genotek Inc. How can I use OMNIgene®•SPUTUM? Use Case 4: Reflex testing: Microscopy centre with or without GeneXpert® centrifuge. 2016 [cited 4 Apr 2017]. Available: www.dnagenotek.com•

18. Corporation P. Maxwell(R) 16 Cell LEV DNA Purification Kit Technical Bulletin TB383. 2012 [cited 11 Mar 2024]. Available: www.promega.com

19. Tahseen S, Qadeer E, Khanzada FM, Rizvi AH, Dean A, Van Deun A, et al. Use of Xpert® MTB/RIF assay in the first national antituberculosis drug resistance survey in Pakistan. International Journal of Tuberculosis and Lung Disease. 2016;20: 448–455. doi:10.5588/ijtld.15.0645

20. Lopes C, Kundu D, Da Costa Barreto I, da Cruz B, Siva Kumar S, Ramalingam S, et al. Experience on the first national anti-TB drug resistance survey (DRS) in Timor-Leste. Glob Health Res Policy. 2022;7: 2–9. doi:10.1186/s41256-022-00249-z

21. Aia P, Kal M, Lavu E, John LN, Johnson K, Coulter C, et al. The burden of drug-resistant tuberculosis in Papua New Guinea: Results of a large population-based survey. PLoS One. 2016;11: 2–9. doi:10.1371/journal.pone.0149806

22. Iem V, Dean A, Zignol M, Vongvichit P, Inthavong D, Siphanthong S, et al. Low prevalence of MDR-TB in Lao PDR: results from the first national anti-tuberculosis drug resistance survey. Tropical Medicine and International Health. 2019;24: 421–431. doi:10.1111/tmi.13205

23. Sharma M, Malhotra B, Tiwari J, Bhargava S. Profile of Nontuberculous Mycobacteria in Patients Suspected of Tuberculosis and Drug-Resistant Tuberculosis. J Lab Physicians. 2020;12: 203–211. doi:10.1055/s-0040-1721160

24. Liu CF, Song YM, He WC, Liu DX, He P, Bao JJ, et al. Nontuberculous mycobacteria in China: incidence and antimicrobial resistance spectrum from a nationwide survey. Infect Dis Poverty. 2021;10. doi:10.1186/s40249-021-00844-1

25. Hoefsloot W, Van Ingen J, Andrejak C, Ängeby K, Bauriaud R, Bemer P, et al. The geographic diversity of nontuberculous mycobacteria isolated from pulmonary samples: An NTM-NET collaborative study. European Respiratory Journal. 2013;42: 1604–1613. doi:10.1183/09031936.00149212

26. Okoi C, Anderson STB, Antonio M, Mulwa SN, Gehre F, Adetifa IMO. Non-tuberculous Mycobacteria isolated from Pulmonary samples in sub-Saharan Africa - A Systematic Review and Meta Analyses. Sci Rep. 2017;7. doi:10.1038/s41598-017-12175-z

27. Aliyu G, El-Kamary SS, Abimiku A, Brown C, Tracy K, Hungerford L, et al. Prevalence of Non-Tuberculous Mycobacterial Infections among Tuberculosis Suspects in Nigeria. PLoS One. 2013;8: e63170. doi:10.1371/journal.pone.0063170

28. Dinic L, Idigbe OE, Meloni S, Rawizza H, Akande P, Eisen G, et al. Sputum Smear Concentration May Misidentify Acid-Fast Bacilli As Mycobacterium Tuberculosis in HIV-Infected Patients. J Acquir Immune Defic Syndr. 2013;63: 168–177. doi:DOI:10.1097/QAI.0b013e31828983b9

29. Kennedy TP, Weber DJ. Nontuberculous mycobacteria. An underappreciated cause of geriatric lung disease. Am J Respir Crit Care Med. 1994;149: 1654–1658. doi:10.1164/ajrccm.149.6.8004325

30. Limo JJ, Nyerere A, Bii C. Prevalence, Species Characterization and factors associated with Non tuberculous mycobacteria among tuberculosis retreatment patients submitting samples to the National Tuberculosis Reference Laboratory, Kenya. Jomo Kenyatta University of Agriculture and Technology. 2016. Available: http://ir.jkuat.ac.ke

31. Xu J, Li P, Zheng S, Shu W, Pang Y. Prevalence and risk factors of pulmonary nontuberculous mycobacterial infections in the Zhejiang Province of China. Epidemiol Infect. 2019;147: e269. doi:10.1017/S0950268819001626

32. Corbett EL, Churchyard GJ, Clayton T, Herselman P, Williams B, Hayes R, et al. Risk Factors for Pulmonary Mycobacterial Disease in South African Gold Miners A Case-Control Study. Am J Respir Crit Care Med. 1999;159: 94–99. doi:https://doi.org/10.1164/ajrccm.159.1.9803048

33. Andréjak C, Nielsen R, Thomsen V, Duhaut P, Sørensen HT, Thomsen RW. Chronic respiratory disease, inhaled corticosteroids and risk of non-tuberculous mycobacteriosis. Thorax. 2013;68: 256–262. doi:10.1136/thoraxjnl-2012-201772

34. Feng JY, Chen WC, Chen YY, Su WJ. Clinical relevance and diagnosis of nontuberculous mycobacterial pulmonary disease in populations at risk. Journal of the Formosan Medical Association. 2020;119: S23–S31. doi:10.1016/j.jfma.2020.05.012

35. Hoza AS, Mfinanga SGM, Rodloff AC, Moser I, König B. Increased isolation of nontuberculous mycobacteria among TB suspects in Northeastern, Tanzania: Public health and diagnostic implications for control programmes. BMC Res Notes. 2016;9. doi:10.1186/s13104-016-1928-3

36. Corbett EL, Churchyard GJ, Hay M, Herselman P, Clayton T, Williams B, et al. The Impact of HIV Infection on Mycobacterium kansasii Disease in South African Gold Miners. Am J Respir Crit Care Med. 1999;160: 10–14. Available: www.atsjournals.org

37. Kone B, Sarro YS, Maiga M, Sanogo M, Somboro AM, Diarra B, et al. Clinical characteristics of non-tuberculous mycobacterial pulmonary infections in Bamako, Mali. Epidemiol Infect. 2018;146: 354–358. doi:10.1017/S0950268817003090

38. Jankovic M, Samarzija M, Sabol I, Jakopovic M, Katalinic Jankovic V, Zmak L, et al. Geographical distribution and clinical relevance of non-tuberculous mycobacteria in Croatia. International Journal of Tuberculosis and Lung Disease. 2013;17: 836–841. doi:10.5588/ijtld.12.0843

39. Sun Q, Yan J, Liao X, Wang C, Wang C, Jiang G, et al. Trends and Species Diversity of Non-tuberculous Mycobacteria Isolated From Respiratory Samples in Northern China, 2014–2021. Front Public Health. 2022;10: 1–8. doi:10.3389/fpubh.2022.923968

40. Farnia P, Farnia P, Ghanavi J, Velayati AA. Epidemiological Distribution of Nontuberculous Mycobacteria Using Geographical Information System. Nontuberculous Mycobacteria (NTM): Microbiological, Clinical and Geographical Distribution. Elsevier; 2019. pp. 191–321. doi:10.1016/B978-0-12-814692-7.00010-3

41. Gonzalo-Asensio J, Pérez I, Aguiló N, Uranga S, Picó A, Lampreave C, et al. New insights into the transposition mechanisms of IS6110 and its dynamic distribution between Mycobacterium tuberculosis Complex lineages. PLoS Genet. 2018;14. doi:10.1371/journal.pgen.1007282

42. Lira LAS, Santos FCF, Carvalho MSZ, Montenegro RA, Lima JFC, Schindler HC, et al. Evaluation of a IS6110-Taqman real-time PCR assay to detect Mycobacterium tuberculosis in sputum samples of patients with pulmonary TB. J Appl Microbiol. 2013;114: 1103–1108. doi:10.1111/jam.12119

43. Hemeg HA, Albulushi HO, Ozbak HA, Ali HM, Alahmadi EK, Almutawif YA, et al. Evaluating the Sensitivity of Different Molecular Techniques for Detecting Mycobacterium tuberculosis Complex in Patients with Pulmonary Infection. Pol J Microbiol. 2023;72: 421–431. doi:10.33073/pjm-2023-040

44. Hanifa Y, Fielding KL, Chihota VN, Adonis L, Charalambous S, Foster N, et al. The utility of repeat Xpert MTB/RIF testing to diagnose tuberculosis in HIV-positive adults with initial negative result. Gates Open Res. 2018;2. doi:10.12688/gatesopenres.12815.1

45. Kolia-Diafouka P, Carrère-Kremer S, Lounnas M, Bourdin A, Kremer L, Van de Perre P, et al. Detection of Mycobacterium tuberculosis in paucibacillary sputum: performances of the Xpert MTB/RIF ultra compared to the Xpert MTB/RIF, and IS6110 PCR. Diagn Microbiol Infect Dis. 2019;94: 365–370. doi:10.1016/j.diagmicrobio.2019.02.008

46. Hamdi A, Fida M, Deml SM, Abu Saleh O, Wengenack NL. Utility of Mycobacterium tuberculosis PCR in ruling out active disease and impact on isolation requirements in a low prevalence setting. J Clin Tuberc Other Mycobact Dis. 2020;21: 1–5. doi:10.1016/j.jctube.2020.100181

47. Fang Y, Wu WH, Pepper JL, Larsen JL, Marras SAE, Nelson EA, et al. Comparison of real-time, quantitative PCR with molecular beacons to nested PCR and culture methods for detection of Mycobacterium avium subsp. paratuberculosis in bovine fecal samples. J Clin Microbiol. 2002;40: 287–291. doi:10.1128/JCM.40.1.287-291.2002

48. Seo AN, Park HJ, Lee HS, Park JO, Chang HE, Nam KH, et al. Performance characteristics of nested polymerase chain reaction vs real-time polymerase chain reaction methods for detecting mycobacterium tuberculosis complex in paraffin-embedded human tissues. Am J Clin Pathol. 2014;142: 384–390. doi:10.1309/AJCP2QZRH4ZNPRDD

49. Dowling WB, Whitelaw A, Nel P. Tracing TB: Are there predictors for active TB disease in patients with Xpert Ultra trace results? International Journal of Infectious Diseases. 2022;114: 115–123. doi:10.1016/j.ijid.2021.10.056

50. Boyles TH, Hughes J, Cox V, Burton R, Meintjes G, Mendelson M. False-positive Xpert® MTB/RIF assays in previously treated patients: Need for caution in interpreting results. International Journal of Tuberculosis and Lung Disease. 2014;18: 876–878. doi:10.5588/ijtld.13.0853

Aissata Boubakar CISSE
Mycobacteriology Unit
Laboratories and Biomedical Research Department
National Institute of Public Health
Bamako (Mali)
Phone: +223 75507091
Email: aissatacisse@yahoo.fr
August 08, 2024

Dear Academic Editor of Plos One journal,

I am pleased to submit herewith responses to journal requirements and remark raised by the reviewer #1 of our manuscript titled "**Prevalence of non-tuberculous mycobacteria among people with acid-fast positive presumptive tuberculosis in Mali**".

Journal Requirements

"Please review your reference list to ensure that it is complete and correct. If you have cited papers that have been retracted, please include the rationale for doing so in the manuscript text, or remove these references and replace them with relevant current references. Any changes to the reference list should be mentioned in the rebuttal letter that accompanies your revised manuscript. If you need to cite a retracted article, indicate the article's retracted status in the References list and also include a citation and full reference for the retraction notice."

We have reviewed our list of references and have not made any changes.

Points raised by Reviewer #1: The revised manuscript contains minor corrections. Author should address the corrections before get published.

We have accepted the minor corrections.
