# Supplementary material for: Prevalence of non-tuberculous mycobacteria among people with acid-fast positive presumptive tuberculosis in Mali
Source: PLoS One. 2024 Dec 18;19(12):e0301731. doi: 10.1371/journal.pone.0301731 (PMC11654988; doi:10.1371/journal.pone.0301731)
Supplement: Attachment — Submitted filename: Response to Reviewers.rtf [file pone.0301731.s002.rtf]

Aissata Boubakar CISSE
Mycobacteriology Unit
Laboratories and Biomedical Research Department
National Institute of Public Health
Bamako (Mali)
Phone: +223 75507091
Email: aissatacisse@yahoo.fr
June 12, 2024
                                                                  Dear Academic Editor of Plos One journal,
I am pleased to submit herewith our point-by-point responses to remarks raised by the academic editor and reviewers of our manuscript titled “Prevalence of non-tuberculous mycobacteria among people with acid-fast positive presumptive tuberculosis in Mali”.
Points raised by academic editor
1.	Please ensure that your manuscript meets PLOS ONE's style requirements, including those for file naming.
We have adapted the manuscript to the style of Plos One, with special attention to the titles and the presentation of the references. 
2.	Please provide additional details regarding participant consent. In the ethics statement in the Methods and online submission information, please ensure that you have specified what type you obtained (for instance, written or verbal, and if verbal, how it was documented and witnessed). If your study included minors, state whether you obtained consent from parents or guardians. If the need for consent was waived by the ethics committee, please include this information. Once you have amended this/these statement(s) in the Methods section of the manuscript, please add the same text to the “Ethics Statement” field of the submission form (via “Edit Submission”).
We have reviewed the ethics section, specifying the type of consent required.
3.	We note that the grant information you provided in the 'Funding Information' and 'Financial Disclosure' sections do not match
We have aligned the grant information in the two sections.
4.	Authors must share the “minimal data set” for their submission
We submitted the minimal data used in the study in the MATLAB and SCV file named “Minimal data set”.
5.	Any changes to the reference list should be mentioned in the rebuttal letter that accompanies your revised manuscript
•	7 references have been removed (1-5, 8 and 38 of initial manuscript);
•	34 references have been retained (11, 12, 14-40, 42, 44, 46, 47, 48 of revised manuscript)
•	16 new references have been added (1, 2, 3, 4, 5-10, 13, 41, 43, 45, 49, 50 of revised manuscript).
Points raised by Reviewers 
Reviewer #1:
1.	Introduction part is not enough and Rewrite the introduction part with the current information. Major revision is required to get published. Rewrite the contents in the methodology and discussion part as mentioned in the reviewed manuscript
We have rewritten the introduction (adding more information about disease burden and laboratory diagnostics, and reorganised the structure), and added some additional information to the methodology and discussion parts. In doing so, we hope to have properly addressed this remark.
Reviewer #2:  
1.	Your article raises several questions about TB diagnosis and provides additional evidence for why smear microscopy as a first line diagnostic tool needed to be replaced with GeneXpert. However, it is interesting that GeneXpert also missed some cases that your inhouse method detected. Could you explain how your method achieved this?
We have provided an explanation for the higher sensitivity of the IS6110 in-house qPCR method compared to Xpert in the discussion part. Line 264-268 in discussion of manuscript: “The observed high Ct values in our study indicates, however, a low quantity of MTB DNA in samples from these patients, that may have been positive upon repeated Xpert testing or by using the Xpert Ultra test, which is more sensitive by targeting both IS6110 and another repetitive element IS1081 for MTBc detection, in addition to the rpoB gene for assessing rifampicin susceptibility”.
2.	Consider changing the title to 'Prevalence of non-tuberculous mycobacteria among acid-fast positive presumptive TB cases in Mali" 
We have accepted to change the title of the manuscript to “Prevalence of non-tuberculous mycobacteria among people with acid-fast positive presumptive TB in Mali” (with a minor edit from “cases” to “people with”).
3.	Based on your results, your in-house qPCR for IS6110 was more sensitive than Xpert as it detected some additional MTBC cases from Xpert negative samples? How sure are you that these additional cases represented ongoing infection with MTBC and not previous exposure? 
This study was part of the national drug-resistant TB survey, comprising both new and previously treated TB cases. The concern of false-positive MTBc detection in sputum from previously treated patients is mostly an issue for the highly sensitive, newer generation of rapid molecular assays, such as a result by “MTB detected - trace” by Xpert Ultra. However, even smear-microscopy could, in theory, identify non-viable AFB. The survey algorithm was not designed to include culture; hence we could not assess the viability of the detected MTBc and/or bacilli detected by microscopy, nor whether this reflected the presence of an NTM in combination with an ongoing MTBc infection, or rather an NTM following a previous MTBc infection. We have included this limitation in the discussion. 
4.	During the survey, at which point were people treated for TB, after ZN staining or based on Xpert results? During the survey was TB culture (gold standard for TB diagnosis) done for the ZN positive samples to confirm ongoing infection?
“All enrolled participants received anti-TB treatment. New patients were started on first-line TB treatment on the basis of an AFB microscopy. For patients with previously treated TB, in accordance with national guidelines, treatment was initiated based on the result of Xpert MTB/RIF testing”. 

5.	If based on Xpert results, what happened to those who were Xpert-negative and later MTBC was detected by your inhouse method?
We have clarified this in the methodology part: “All enrolled participants received anti-TB treatment. New patients were started on first-line treatment based on the microscopy results. For previously treated patients, in accordance with national guidelines, re-treatment or RR-TB treatment was initiated based on Xpert's rifampicin-susceptibility result”. Hence, no anti-TB treatment had to be started based on the in-house qPCR results because the results were received 1 year later. 
6.	The conclusion that there is the need for alternative diagnostics beyond Xpert is valid, as your study found that some Xpert-negative and AFB-positive samples could actually still be MTBC positive by other methods. However, it would be important to state that such methods would need to be validated with TB culture which is the gold standard.
We added this reflection in the discussion (line 287-289) and described also the place of culture for the diagnosis of NTM and monitoring of the treatment in the discussion part: “Nevertheless, even smear microscopy can still show non-viable AFB, and introduction of alternative/additional diagnostic assays or flows would need to be validated against culture”.
7.	For general comments:
•	Line 154-157: The sentence is too long and confusing, kindly rephrase using shorter sentences for easy comprehension.
We rewrote the sentence.
•	Line 184: change from "male patients were more numerous” to "male patients were more".
We rephrased this sentence.
•	Line 214-216: "The proportion of NTM versus Nocardia spp note the clinical relevance of these isolated bacteria were discussed in that study". What is the relevance of the statement? Can you rephrase for clarity?
We removed the ambiguous sentence.
•	Line 252: Please check the arrangements of the references-some have been repeated and are outside the bracket.
We checked and corrected where needed.
•	Check spacing before references in brackets- In some instances, there is no space between the last word and the reference number. Please be consistent with this throughout the manuscript.
We checked and corrected where needed.
