# Supplementary material for: Prevalence of non-tuberculous mycobacteria among people with acid-fast positive presumptive tuberculosis in Mali
Source: PLoS One. 2024 Dec 18;19(12):e0301731. doi: 10.1371/journal.pone.0301731 (PMC11654988; doi:10.1371/journal.pone.0301731)
Supplement: Attachment — Submitted filename: Response to Reviewers.rtf [file pone.0301731.s004.rtf]

Aissata Boubakar CISSE
Mycobacteriology Unit
Laboratories and Biomedical Research Department
National Institute of Public Health
Bamako (Mali)
Phone: +223 75507091
Email: aissatacisse@yahoo.fr
August 08, 2024
                                                                  Dear Academic Editor of Plos One journal,
I am pleased to submit herewith responses to journal requirements and remark raised by the reviewer #1 of our manuscript titled “Prevalence of non-tuberculous mycobacteria among people with acid-fast positive presumptive tuberculosis in Mali”.
Journal Requirements
“Please review your reference list to ensure that it is complete and correct. If you have cited papers that have been retracted, please include the rationale for doing so in the manuscript text, or remove these references and replace them with relevant current references. Any changes to the reference list should be mentioned in the rebuttal letter that accompanies your revised manuscript. If you need to cite a retracted article, indicate the article's retracted status in the References list and also include a citation and full reference for the retraction notice.”
We have reviewed our list of references and have not made any changes.
Points raised by Reviewer #1 
“Manuscript focused mainly on NTM. If author had used few reference stain (ATCC), manuscript would have completed scientifically. Otherwise the manuscript required minor revision”
We have added some reference strains used for our sequence analysis. We described it in the manuscript on lines 153-157: “Sequencing was subcontracted to BaseClear (BE Leiden, The Netherlands). After entering the sequence into the Basic Local Alignment Search Tool (BLAST) of the National Centre for Biotechnology Information (NCBI), we compared it to reference strains sequences on NCBI such as ATCC 25791, ATCC BAA-614, ATCC 27282, ATCC 51457, 4773, ATCC 700504, ATCC 25276, ATCC 35752, ATCC 13950”.
Thank you for your attention and the time you will take to re-evaluate this work after we have considered the different comments.
