# Supplementary material for: Prevalence of non-tuberculous mycobacteria among people with acid-fast positive presumptive tuberculosis in Mali
Source: PLoS One. 2024 Dec 18;19(12):e0301731. doi: 10.1371/journal.pone.0301731 (PMC11654988; doi:10.1371/journal.pone.0301731)
Supplement: Attachment — Submitted filename: Response to Reviewers.rtf [file pone.0301731.s008.rtf]

Aissata Boubakar CISSE
Mycobacteriology Unit
Laboratories and Biomedical Research Department
National Institute of Public Health
Bamako (Mali)
Phone: +223 75507091
Email: aissatacisse@yahoo.fr
October 22, 2024
                                                                  Dear Academic Editor of Plos One journal,
I am pleased to submit herewith responses to journal requirements and remark raised by the reviewer #1 of our manuscript titled “Prevalence of non-tuberculous mycobacteria among people with acid-fast positive presumptive tuberculosis in Mali”.
Journal Requirements
“Please review your reference list to ensure that it is complete and correct. If you have cited papers that have been retracted, please include the rationale for doing so in the manuscript text, or remove these references and replace them with relevant current references. Any changes to the reference list should be mentioned in the rebuttal letter that accompanies your revised manuscript. If you need to cite a retracted article, indicate the article's retracted status in the References list and also include a citation and full reference for the retraction notice.”
We have reviewed our list of references and have not made any changes.
Points raised by Reviewer #1: This manuscript require minor revision before to get published as per the attachment of the revised manuscript.
We have accepted the minor corrections. But we proposed for sentence 205-206: “The microscopy laboratories provided AFB quantification result for 1414 of the 1418 patients” instead of “The participating laboratories were informed of the quantification of AFB for 1414 out of 1418 patients”.

Yours sincerely
